# Differential mortality risks associated with ambient temperature among migrants and non-migrants: a space-time-stratified case-crossover study of the 100 Million Brazilian Cohort (2000–2020)

**DOI:** 10.1016/j.lana.2026.101687

**Published:** 2026-10-27

**Authors:** Neda Firouraghi, Taisa R. Cortes, Ludmilla V. Jacobson, Danielson J.D. Neves, Ismael H. Silveira, Shakoor Hajat, Mauricio L. Barreto, Julia M. Pescarini

**Affiliations:** a Infectious Disease Epidemiology & International Health, Faculty of Epidemiology and Population Health, London School of Hygiene and Tropical Medicine, London, UK; b Centro de Integração de Dados e Conhecimentos para Saúde (Cidacs), Fundação Oswaldo Cruz, Salvador, Brazil; c Department of Statistics, Institute of Mathematics and Statistics, Fluminense Federal University, Niterói, Brazil; d National Institute for Space Research (INPE), Cachoeira Paulista, Brazil; e Institute of Collective Health, Federal University of Bahia, Salvador, Brazil; f Faculty of Public Health and Policy, Department of Public Health, Environments and Society, London School of Hygiene and Tropical Medicine, London, UK

**Keywords:** Extreme ambient temperature, Migration, Socially vulnerable population, Mortality, Case-crossover study, Distributed lag non-linear model (DLNM)

## Abstract

**Background:**

Climate change disproportionately affects socially minoritized populations, but migrants' vulnerability to extreme temperatures remains overlooked. We estimated short-term associations between extreme ambient temperatures and mortality among migrants and non-migrants across Brazil.

**Methods:**

We linked the 100 Million Brazilian Cohort to mortality and temperature data (2000–2020) and conducted a space-time-stratified case-crossover study analysing the differential effects of extreme temperature on mortality among internal migrants (i.e., Individuals whose state of birth was different from their residence at registration or death) and non-migrants. We used conditional quasi-Poisson regression with distributed lag non-linear model (DLNM) to estimate relative risks (RRs) and 95% confidence intervals (CIs) for all-cause non-accidental and cause-specific mortality associated with extreme-cold (1st percentile) and extreme-heat (99th percentile) compared to minimum-mortality-temperature (i.e., the temperature associated with the lowest mortality risk), over 0–20 days in both groups. We further stratified by age, literacy, urbanicity and housing conditions.

**Findings:**

We included 1,199,636 deaths among internal-migrants (mean age at death = 62.67 years (standard-deviation (SD) = 18.36), with 673,166 (56%) males) and 3,716,900 deaths among non-migrants (57.18 years (SD = 20.31), with 2,135,293 (57%) males). Over 0–20 days, we observed a consistent pattern of higher risks of all-cause, non-accidental mortality among internal-migrants than non-migrants following both extreme-heat (RR_Mig_(95%CI):1.05(1.01–1.10) vs. RR_Non-mig_:1.01(0.99–1.04)) and extreme-cold (RR_Mig_:1.27(1.22–1.32) vs. RR_Non-mig_:1.24(1.20–1.28)). Extreme cold-related circulatory mortality (mortality due to diseases of the circulatory system, based on the relevant International Classification of Diseases codes) (I00–I99) was higher among non-migrants (RR_Mig_:1.32(1.20–1.45) vs. RR_Non-mig_:1.41(1.31–1.51)) while heat-related respiratory mortality (J00–J99) was higher among internal-migrants (RR_Mig_:1.32(1.10–1.58) vs. RR_Non-mig_:1.06(1.00–1.17)), particularly those illiterate (RR_Mig_:1.84(1.25–2.71) vs. RR_Non-mig_:1.10(0.89–1.36)). Other subcategories showed greater 95%CI overlap.

**Interpretation:**

We found internal migrants are likely to experience higher heat-related respiratory mortality than non-migrants particularly low-educated migrants. Our results highlight the need to further consider migrants for targeted climate adaptation policies, with a focus on reducing social inequalities in the place of arrival.

**Funding:**

The Wellcome Trust.

**Research in context**

**Evidence before this study**

Extreme temperatures, increased in duration and frequency due to climate change, impacts individual health and increases mortality rates. These effects could be intensified among migrants, who usually live in the most deprived areas. We searched PubMed and Embase on 12 February 2026, without applying any date restrictions, for the existing evidence on the effects of extreme ambient temperatures on mortality among migrant populations using the following: (“ambient temperature” OR “climate change” OR “heat” OR “cold” OR “extreme weather”) AND (“immigration” OR “migration” OR “migrant∗” OR “immigrant∗” OR “displace∗” OR “refugee∗” OR “asylum seek∗” OR “mobilit∗”) AND “mortality”), filtered by humans. After removing duplicates, we screened 243 papers and identified 8 articles looking at the impact of extreme temperatures on mortality among migrants, of which four looked at the effects among international migrants and non-migrants in the country of arrival (i.e., in the US, France, Kuwait and Jordan); three only looked at migrants in informal settings or migrant workers (i.e., one in Qatar and two in Kenya but without also estimating the effects among non-migrants); and only one looked at both international and internal migrants from the rest of China in Hong Kong comparing that to non-migrants. In China, immigrants (both internal and international) experienced higher mortality during heatwaves than non-migrants. No studies have evaluated climate-related mortality among migrants in Latin America, including Brazil.

**Added value of this study**

Unlike previous studies, which focused mainly on international migrants or did not compare them with local non-migrant populations, our study directly compares internal migrants with non-migrants within a large cohort of socioeconomically disadvantaged populations including over 144 million individuals in 21 years. This study advances previous temperature–mortality analyses in general population of Brazil by examining migration status as a modifier of climate related mortality risk to investigate whether internal migrants experience disproportionate vulnerability to extreme-temperature exposure compared with non-migrants living under similar socioeconomic profiles. Aggregating the data at the level of immediate geographic regions (IGR) improved the statistical power of this study to analyses of cause-specific mortality by considering socioeconomic inequality and housing conditions. We identified migrants as a structurally vulnerable population facing elevated climate-related all-cause and heat-related respiratory mortality risk, particularly among lower-educated groups. These findings extend the climate-health literature beyond overall population burden estimates toward understanding social heterogeneity and inequities in temperature-related mortality in low- and middle-income settings.

**Implications of all the available evidence**

This study fills an important gap in the literature beyond high-income settings by providing large-scale evidence from a low- and middle-income country showing that internal migrants experience elevated temperature-related mortality, even within a socioeconomically disadvantaged cohort. The elevated risks among lower-educated migrants indicate that educational inequalities may shape climate-related health vulnerabilities after migration. These findings highlighted the need to incorporate internal migrants into climate adaptation and public health strategies.

## Introduction

Extreme ambient temperatures pose significant risks to physical and mental well-being, which are expected to intensify under climate change.^1^, ^2^ Although it is expected that extreme temperature disproportionately affects vulnerable populations such as minoritized people,^2^, ^3^ indigenous groups,^4^ people experiencing homelessness and migrants,^5^ quantitative evidence remains scarce. Importantly, the specific needs of displaced populations and migrants are largely overlooked and there are widespread calls from United Nations (UN) and the World Health Organization (WHO) to specifically measure migrants’ health and their vulnerability to climate change.^6^, ^7^, ^8^

Migrants are a heterogeneous group who migrate for diverse reasons and face varying challenges during and after relocation.^9^, ^10^ Migrants’ health can be affected through multiple pathways, including precarious living and working conditions, barriers to healthcare, social exclusion, discrimination and financial instability, which may lead to long-lasting impacts on physical and mental health.^9^, ^10^, ^11^ In addition, overcrowded housing, precarious employment, environmental hazards, and barriers to healthcare may contribute to a higher burden of diseases in some migrant communities.^9^, ^10^, ^11^, ^12^, ^13^ However, in many countries, groups of migrants remain excluded from national health programs for prevention and treatment.^14^

Brazil has a long history of internal migration,^15^ which have often served as a long-term adaptation strategy to environmental pressures, poverty, and inequality.^16^, ^17^ The 2022 Brazilian Census indicates that 29 million Brazilians (approximately 14% of the population) reside outside their State of birth^15^; the official Brazilian definition of internal migrants. Commercial farming and urban economic opportunities in centres, such as São Paulo and Rio de Janeiro, push people to rural-to-urban transition, which leads to limited housing and overcrowding.^16^, ^17^, ^18^ Migrants can become vulnerable due to poor living and working conditions, which are often worsened by sociodemographic characteristics.^19^ Previous research shows Brazil's internal migrants experience heterogeneous outcomes shaped by education and economic capacity.^11^

Brazil is a large country encompassing multiple climatic zones, biomes and with large socioeconomic inequalities which has faced increasingly frequent and intense extreme temperature events in recent years.^20^ Previous studies report increased mortality associated with extreme temperatures in Brazil, both in the general population^21^ and among low-income individuals.^2^ Cold-related mortality was higher in older adults and Black populations, while heat-related mortality was greater in urban areas.^2^ Migrants may be particularly at risk, as their living and working conditions and socioeconomic status may increase temperature exposure and reduce adaptive capacity.^11^, ^18^, ^21^ Without tailored adaptation measures, extreme temperature-related mortality is expected to escalate.^22^

Considering the knowledge gap on temperature-related mortality among migrants, especially in Latin America, and the large social and environmental vulnerabilities, we hypothesised that the association between ambient temperature and mortality would differ between internal migrants and non-migrants owing to differences in socioeconomic, living and working conditions. We therefore investigated the short-term associations between ambient temperature and all-cause and cause-specific mortality among internal migrants and non-migrants. In this study, we utilised the 100 Million Brazilian Cohort (*100M Cohort)*, a detailed socioeconomic and demographic data of the low-income families across Brazil over 21 years,^23^ linked with mortality and ambient temperature datasets. This cohort is particularly well suited to address our question, as it enables assessment of heterogeneity across sociodemographic characteristics and living conditions.

## Methods

### Study design

We conducted a space-time-stratified case-crossover design to evaluate the association between daily mean temperature and daily deaths in Brazil. This method is widely used for transient environmental exposures and inherently controls for time-invariant individual characteristics, using either individual-level or aggregated data.^24^

### Study population and definitions

We studied all deaths among individuals aged 18–110 years at the date of death within the studied period from 1st January 2000 to 31st December 2020 registered in the *100M Cohort*. We defined migration based on the place of residence at birth, residency recorded at *CadUnico* registration, and place of residence at death. International migrants were individuals born in another country. Because many births are registered in neighbouring municipalities rather than the actual municipality of residence, migration was defined at the state level instead of the municipal level. Internal migrants were operationally defined as individuals whose state of birth differed from the state recorded at CadÚnico registration or at death, indicating migration between the 27 Brazilian states (i.e., 26 States plus the Federal District). Non-migrants were those whose state of birth, registration and death were the same. This definition captures interstate migration recorded in the available administrative data but does not capture the timing, duration, frequency, or multiple migration events during an individual's lifetime, because complete residential histories were unavailable. Migrant status was hypothesised to be a key effect modifier of the relationship between ambient temperature and death. We excluded individuals with missing values for place of residency at birth, registry or death.

### Data sources

Three main data sources were used in this study. First, we used data from *100M Cohort*,^23^ a cohort of low-income families registered in the Unified registry for Social Programs (Cadastro Único -CadÚnico). The registry is required to access Brazilian federal social programs and includes individual-level sociodemographic and economic data of people with low family income. The cohort baseline was linked to mortality data from the Brazilian *Mortality Information System (SIM),* the official nationwide mortality surveillance system provides standardised information on date, place of residence and underlying cause of death coded according to the International Classification of Diseases, 10th Revision (ICD-10), from 2000 to 2020. The completeness and quality of mortality reporting have improved substantially over time and with coverage >95% in recent years.^25^ The non-deterministic linkage based on name, name of the mother, sex, date of birth, and municipality of residence was conducted by the Center for Data and Knowledge Integration for Health (CIDACS) using a record linkage method (CIDACS-RL) designed to conduct large-scale observational research with Brazilian administrative data.^26^ We also obtained daily minimum and maximum temperature data from the Brazilian Daily Weather Gridded Data (BR-DWGD) with a spatial resolution of 0.1° × 0.1° (∼10 km).^27^

### Exposure

Our main exposure was daily mean ambient temperature at the Immediate Geographic Region (IGR) level of residency at death. IGRs are clusters of municipalities organised around urban centres that concentrate everyday population activities related to work, health care, education, and other basic public services,^28^ and an official administrative division defined by the Brazilian Institute of Geography and Statistics (IBGE). IGRs were chosen to ensure sufficient daily death counts for stable estimation, and the related exposure was calculated using population-weighted daily mean temperature estimates of the municipalities that form an IGR.

To calculate the daily mean temperature, we used BR-DWGD,^29^, ^30^ which provides spatially continuous maximum and minimum temperature estimates across Brazil by interpolating observations from 1252 national meteorological conventional and automatic stations using Inverse Distance Weighting (IDW) and correcting for the elevation and lapse-rates to account for terrain-driven temperature differences, thereby reducing the impact of uneven station distribution. BR-DWGD construction deals with missing data in station observations through a qualitative test for homogeneity assessment that incorporates data from the five nearest surrounding stations.^30^ As BR-DWGD doesn't provide daily mean temperature, we approximated it by calculating the average of the daily maximum and minimum temperatures.

We used BR-DWGD dataset together with annual population density data from WorldPop^31^ with a spatial resolution of 0.01° × 0.01° (∼1 km), to calculate the municipal population-weighted mean temperature and then aggregated this exposure to the IGR level. To account for the different spatial resolutions of the temperature and population datasets, we resampled the WorldPop population density raster to the coarser resolution of BR-DWGD grid using a spatial averaging method. The municipal-level population-weighted mean temperature was then calculated as the weighted average of the temperature grids intersecting each municipality, where the weights combined both the grid's intersecting area and its resampled population density. This was done using the function “exact_extract” from the R package “exactextractr”.

This approach assumes that the IGR of residency at death approximates the location where individuals experienced ambient temperature during the short exposure window. Individual mobility within or between IGRs during the lag period could not be accounted for.

### Outcome

Outcomes included all-cause/non-accidental and cause-specific mortality coded using ICD-10. All-cause non-accidental mortality included deaths with underlying causes coded A00 to R99, excluding external causes (V01–Y98), to isolate physiological vulnerability and disease-related effects of thermal stress. For cause-specific mortality, we included disease of the circulatory system (I00–I99), respiratory system (J00–J99) and renal failure (N17–N19). All analyses were conducted at the IGR level. Deaths were assigned to the corresponding IGR based on the municipality of residence recorded on the death certificate. Daily mortality counts were then aggregated by IGR and linked to daily ambient temperature for the corresponding IGR.

### Ethics

The 100 Million Brazilian Cohort received approval from the ethics committees at Instituto Gonçalo Muniz of Oswaldo Cruz Foundation (Ref 6.745.063/2024). This study received approval from the ethics committees at Instituto Gonçalo Muniz of Oswaldo Cruz Foundation and the Brazilian National Commission of research Ethics (CONEP) (7.828.749/2025), and from The London School of Hygiene and Tropical Medicine (Ref 31453/RR/40149 in 2024).

### Statistical analysis

The associations between ambient temperature and all-cause and cause-specific mortality among migrants and non-migrants were investigated using conditional quasi-Poisson regression models with a distributed lag non-linear model (DLNM).^32^ Conditional quasi-Poisson models are appropriate for analysing a space-time-stratified case-crossover design with time-series data, in which daily and spatially aggregated case counts are analysed rather than individual data.^24^ Our defined stratum to integrate temporal and spatial dimensions were IGR and day. Case days are matched with control days that fall on the same day of the week, within the same month and year, and within the same geographic location (IGR). This matching strategy effectively controls for biases related to long-term time trends, seasonal patterns, day-of-week effects, time-invariant individual-level factors, and spatial characteristics. For example, if the case IGR-day was the last Wednesday of January 2020 (29-January-2020), within the specific IGR (e.g., IGR code:110001), it was compared with four previous Wednesdays of January 2020:22-January-2020, 15-January-2020, 08-January-2020, 01-January-2020; at the same IGR.

The temperature-mortality association was modelled using a lag period of 0–20 days to capture both the delayed effects of cold and the effect of heat exposure. A shorter lag period (0–6 days) was also examined as sensitivity analysis to assess the robustness of the findings to alternative lag specifications. Temperature was modelled using distributed lag non-linear model^32^ and cross-basis function with a natural cubic spline (ns) for both the exposure-response and lag-response dimensions. Two internal knots were placed at the 25th and 75th percentiles of the temperature distribution for the exposure-response function. For the lag dimension, three knots were placed at logarithmically spaced values across the lag period, guided by prior studies conducted in Brazil.^2^ We estimated relative risks (RRs) and 95% confidence intervals (CIs) for extreme cold-related mortality at the 1st percentile and extreme heat-related mortality at the 99th percentile of temperature distribution for each group. These thresholds are widely used in temperature-mortality studies to represent the tails of the exposure distribution and identify intense cold and heat exposures,^33^, ^34^, ^35^ and this approach is consistent with previous study using the 100 Million Brazilian Cohort.^2^ The minimum mortality temperature (MMT) was estimated separately for each fitted model/sub-group, and relative risks were calculated after centring the exposure-response function at the corresponding subgroup-specific MMT. When the MMT was outside the range defined by the heat and cold thresholds, the median value was used instead.

### Stratified analysis, robustness checks and sensitivity analysis

To better understand the associations between ambient temperature and mortality across migrant subgroups, we developed a conceptual framework of the potential mechanisms underlying health outcomes among migrants (Supplementary Figure S1). We further conducted stratified analysis by age, literacy, urbanicity, and housing conditions among internal migrants and non-migrants to assess differences in mortality by individual and structural characteristics. In addition, we identified the most common migration pathways between regions in Brazil and conducted stratified analyses for these patterns, including movements within the Northeast, within the Southeast, and from the Northeast to the Southeast. We further examined migration flows according to the population size of the origin and destination municipalities within each state. Municipalities were classified into four categories based on their resident population (<200,000, 200,000–499,999, 500,000–999,999, and ≥1,000,000 inhabitants). Migration flows were then defined according to the combination of origin and destination municipality population categories. To ensure a sufficient number of cases for stable DLNM estimation, we focused on two key migration patterns: movements within less populated municipalities (within municipalities <200,000 inhabitants) and movements from less/middle populated to more populated municipalities (<500,000 to ≥500,000 inhabitants). Sensitivity analyses were conducted to assess the robustness of the results to different model specifications and assumptions, by (1) including analysis with a shorter lag period (0–6 days), (2) redefining migration at the municipality level, (3) applying the median as the reference for RRs instead of using the MMT, (4) including genitourinary system mortality as specific cause of death, (5) using alternative knot placements (at the 10th and 90th percentiles) in the temperature cross-basis (6) stratifying by biome of the residency of death, and (7) including interaction terms between the temperature cross-basis and migration status and calculating the likelihood ratio test to formally assess effect modification by migration status (Supplementary File). Statistical analyses were done using R (version 4.1.3) with the *gnm* and *dlnm* packages. All analyses were conducted in the CIDACS/Fiocruz secure virtual machine environment.

### Role of the funding source

The funders had no role in the study design, analysis, interpretation, or writing of the manuscript.

## Results

We analysed 4,935,541 deaths reported on the *100M Cohort* between 2000 and 2020 (See Supplementary Figure S2). Of the total deaths, 1,199,636(24.3%) occurred among internal migrants, 19,005(0.4%) among international migrants, and 3,716,900(75.3%) among non-migrants (see Table 1). Internal migrants and non-migrants had similar racial composition, with only minor differences. Unemployment was high across all groups but slightly higher among internal migrants (608,843(50.75%)) than non-migrants (1,817,992(48.91%)). Literacy was slightly lower among internal migrants (752,347(62.71%)) than non-migrants (2,404,984(64.70%)). Rural residence was more common among non-migrants (851,907(22.92%)) than internal migrants (183,018 (15.26%)), while internal migrants were more likely to reside in urban areas. Most internal migrants (1,083,236(90.30%)) and non-migrants (3,422,347(92.08%)) lived in permanent private dwellings. Characteristics of international migrants are presented in Table 1.

**Table 1:** Sociodemographic characteristics, living conditions, and mortality causes among deceased internal, international, and non-migrants in the 100 Million Brazilian Cohort (2000–2020).

Characteristic	Internal migrant (N = 1,199,636)	International migrant (N = 19,005)	Non-migrant (N = 3,716,900)
Sociodemographic characteristics			
Age (mean years; SD)			
At the time of registry	56.17 (19.03)	62.75 (15.75)	49.82 (20.90)
At the time of death	62.67 (18.36)	66.88 (15.54)	57.18 (20.31)
Sex, n (%)			
Male	673,166 (56%)	12,384 (65%)	2,135,293 (57%)
Female	526,470 (44%)	6621 (35%)	1,581,607 (43%)
Race, n (%)			
White	416,041 (34.68%)	8785 (46.2%)	1,177,015 (31.67%)
Black	105,133 (8.77%)	1550 (8.2%)	378,864 (10.19%)
Asian/“Yellow”	4924 (0.41%)	563 (3.0%)	13,827 (0.37%)
Brown/“Pardo”	583,968 (48.68%)	7220 (38%)	1,823,550 (49.06%)
Indigenous	2424 (0.20%)	55 (0.3%)	16,220 (0.44%)
Missing	87,146 (7.26%)	832 (4.4%)	307,424 (8.27%)
Employment, n (%)			
Employee without a formal contract/Self-employed worker	120,032 (10%)	1192 (6.27%)	360,071 (9.69%)
Employee with a formal contract (e.g., military or public servant)	32,949 (2.75%)	278 (1.46%)	95,255 (2.56%)
Unpaid worker/Intern/Apprentice	539 (<0.1%)	33 (0.17%)	1719 (<0.1%)
Retired/Pensioner	219,487 (18.30%)	752 (3.96%)	663,560 (17.85%)
Rural worker	69,120 (5.76%)	177 (0.93%)	336,942 (9.07%)
Unemployed	608,843 (50.75%)	9261 (48.73%)	1,817,992 (48.91%)
Other	72,038 (6.00%)	154 (0.81%)	244,666 (6.58%)
Missing	76,628 (6.39%)	7158 (37.66%)	196,695 (5.29%)
Literacy, n (%)			
Yes	752,347 (62.71%)	14,643 (77.05%)	2,404,984 (64.70%)
No	390,774 (32.57%)	4247 (22.35%)	1,101,804 (29.64%)
Missing	56,515 (4.71%)	115 (0.61%)	210,112 (5.65%)
Living area and housing conditions			
Urbanicity, n (%)			
Urban	981,690 (81.83%)	16,739 (88.08%)	2,743,550 (73.81%)
Rural	183,018 (15.26%)	2150 (11.31%)	851,907 (22.92%)
Missing	34,928 (2.91%)	116 (0.61%)	121,443 (3.27%)
Domicile type, n (%)			
Permanent private	1,083,236 (90.30%)	12,975 (68.27%)	3,422,347 (92.08%)
Other	48,027 (4.00%)	664 (3.49%)	121,176 (3.26%)
Missing	68,373 (5.70%)	5366 (28.23%)	173,377 (4.66%)
Household building material, n (%)			
Brick/Masonry	882,222 (73.54%)	14,540 (76.51%)	2,685,144 (72.24%)
Other (e.g., wood, clad rammed earth, uncoated rammed earth)	261,917 (21.83%)	3738 (19.67%)	859,322 (23.12%)
Missing	55,497 (4.63%)	727 (3.83%)	172,434 (4.64%)
Household lighting, n (%)			
Electricity with own metre	961,318 (80.13%)	16,049 (84.45%)	2,950,173 (79.37%)
Electricity with community/Without metre	114,160 (9.52%)	1591 (8.37%)	303,412 (8.16%)
Other form (e.g., oil, kerosene, candle, gas)	68,656 (5.72%)	639 (3.36%)	290,943 (7.83%)
Missing	55,502 (4.63%)	726 (3.82%)	172,372 (4.64%)
Sewage, n (%)			
Public network	559,335 (46.63%)	11,514 (60.58%)	1,640,730 (44.14%)
Septic tank	183,726 (15.32%)	2134 (11.23%)	527,766 (14.20%)
Others (e.g., rudimentary tank, trench)	394,010 (32.84%)	4331 (22.79%)	1,356,595 (36.50%)
Missing	62,565 (5.22%)	1026 (5.40%)	191,809 (5.16%)
Water supply, n (%)			
Public Network	888,583 (74.07%)	15,282 (80.41%)	2,584,207 (69.53%)
Well/Spring	201,972 (16.84%)	2197 (11.56%)	700,591 (18.85%)
Other (e.g., water truck)	53,554 (4.46%)	800 (4.21%)	259,671 (6.99%)
Missing	55,527 (4.63%)	726 (3.82%)	172,431 (4.64%)
Cause of mortality, n (%)			
All cause, non-accidental	1,046,444 (87%)	17,609 (92.65%)	3,041,880 (82%)
Circulatory system	351,610 (33.60%)	5623 (31.93%)	936,032 (30.77%)
Respiratory disease	124,589 (11.91%)	2274 (12.91%)	330,090 (10.85%)
Renal failure	13,198 (1.26%)	208 (1.18%)	38,640 (1.27%)
Other non-accidental	557,047 (53.23%)	9504 (53.97%)	1,737,118 (57.11%)
All-cause, accidental	153,192 (13%)	1396 (7.35%)	675,020 (18%)

SD, standard deviation.

Values are presented as mean (SD) for continuous variables and number (%) for categorical variables.

Among internal migrants and non-migrants, mortality risk increased at both lower and higher temperatures (Fig. 1). Cold-related mortality was more pronounced over the long-lag period, whereas heat-related associations were stronger in the short-lag period for both non-migrants and internal migrants. When examining the estimated relative risks at the 1st and 99th percentile for extreme temperature-mortality associations among non-migrants and internal migrants (see Fig. 1, Supplementary Tables S1 and S2), we observed a consistent pattern that internal migrants tended to experience higher all-cause, non-accidental mortality risks than non-migrants after both extreme-heat (RR_Mig_(95%CI):1.05(1.01–1.10) vs. RR_Non-mig_:1.01(0.99–1.04)) and extreme-cold (RR_Mig_(95%CI):1.27(1.22–1.32) vs. RR_Non-mig_:1.24(1.20–1.28)) over 0–20 days. The risk of respiratory disease mortality among internal migrants was likely higher than that among non-migrants after extreme-heat (RR_Mig_(95%CI):1.32(1.10–1.58) vs. RR_Non-mig_:1.06(1.00–1.17)) and extreme-cold (RR_Mig_(95%CI):1.30(1.11–1.52) vs. RR_Non-mig_:1.28(1.15–1.43)) over 0–20 days. Although the point estimates suggest a higher heat-related respiratory mortality risk among internal migrants, the overlapping 95%CIs indicate that this between-group difference should be interpreted with caution. However, internal-migrants showed a tendency toward lower circulatory system mortality risk after extreme-heat (RR_Mig_(95%CI):0.93(0.84–1.03) vs. RR_Non-mig_:1.04(0.99–1.09)) and extreme-cold (RR_Mig_(95%CI):1.32(1.20–1.45) vs. RR_Non-mig_:1.41(1.31–1.51)) over 0–20 days. The association after extreme heat was not significant in either group. For renal failure mortality, due to limited numbers, we only observed evidence of excess mortality risk among non-migrants after extreme cold (RR:1.27; 95%CI:1.05–1.55) and extreme-heat (RR:1.23; 95%CI:1.04–1.45) over 0–6 days (Fig. 1, Supplementary Tables S1 and S2). Due to the limited number of international migrants registered in the cohort, evidence of associations was observed only for all-cause mortality after extreme cold over a lag of 0–20 days (RR:1.96; 95%CI:1.10–3.50) (Supplementary Figure S3, Supplementary Tables S1 and S2).

**Fig. 1:**
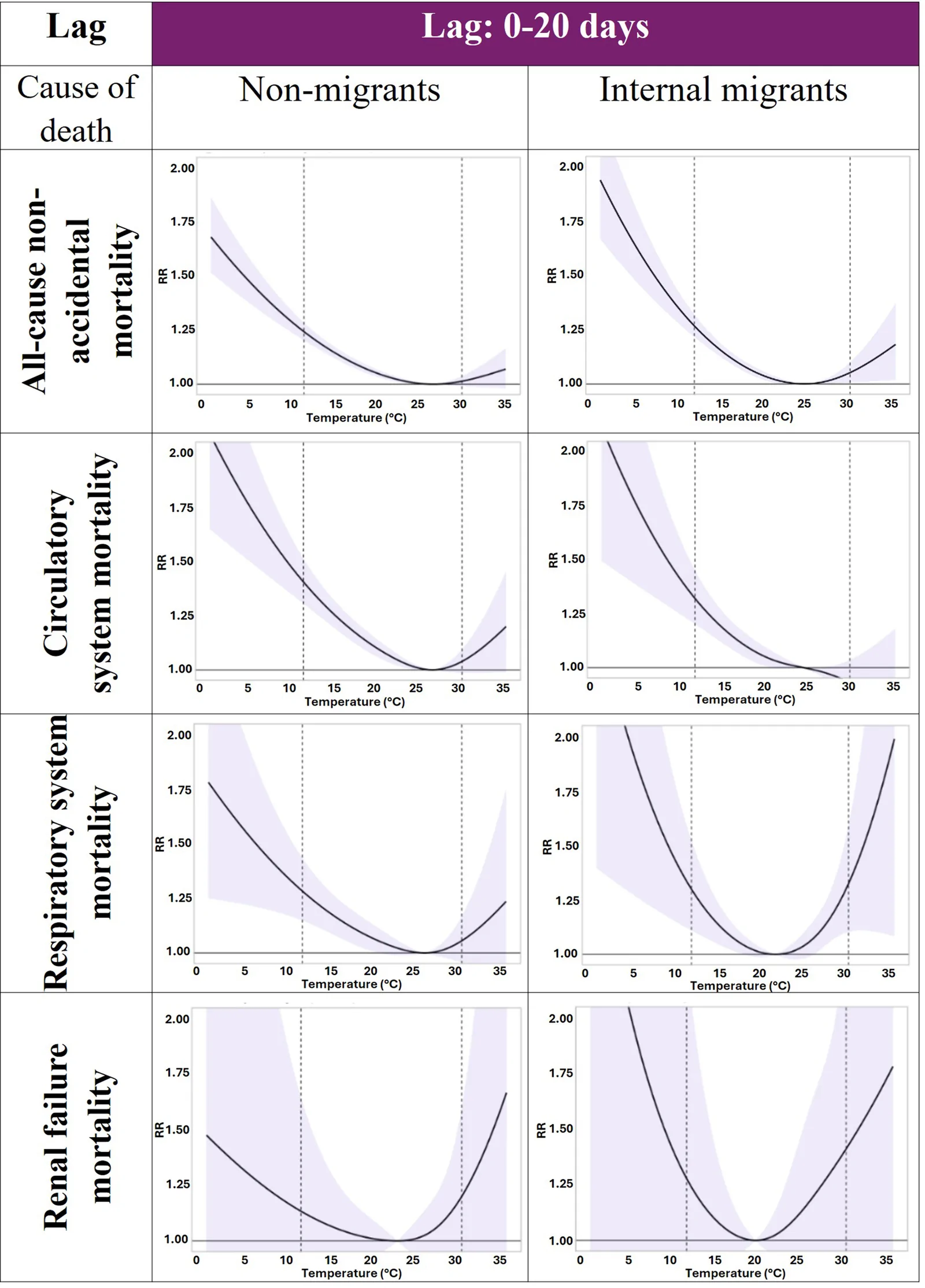
Exposure-response association between ambient temperature (°C) and all-cause and cause-specific mortality among non-migrants and internal migrants over 0–20 days in the 100 Million Brazilian Cohort (2000–2020). The shaded areas represent the 95% confidence intervals, and the dotted lines indicate the 1st (extreme-cold) and 99th (extreme-heat) percentiles of the weighted mean temperature distribution. Temperature (°C) is shown on the x-axis and relative risk (RR) on the y-axis. The solid line represents the estimated relative risk.

Over 0–6 days, the sensitivity analysis produced findings consistent with the main analysis. Internal migrants continued to show higher all-cause and respiratory mortality risks, whereas circulatory mortality risk remained lower than that observed among non-migrants (Supplementary Figure S4 and Supplementary Tables S1 and S2).

Cold temperatures were associated with delayed increases in mortality risk, while heat was associated with sharper and more immediate increases in risk (Supplementary Figure S5). Lag-response curves revealed contrasts between extreme-heat and cold. Heat-related mortality risks peaked immediately (lag 0–1 days) and declined rapidly. In contrast, cold exposure showed an initial protective association during the first few days, followed by a delayed increase in risk peaking at 2–5 days and almost remaining elevated for up to 20 days (Supplementary Figure S6).

Over the 0-20-day lag, extreme-cold remained associated with higher mortality risk, while extreme-heat showed no substantial association with all-cause or circulatory mortality among both non-migrants and migrants. When examining the migration routes, migrants who moved from the Northeast to the Southeast, the most common migration route in Brazil (see Supplementary Tables S3–S5), showed lower risk of all-cause mortality (RR:1.23; 95%CI:1.16–1.30) and circulatory mortality (RR:1.24; 95%CI:1.10–1.41) than migrants who moved within the Southeast (RR:1.33; 95%CI:1.21–1.45) and (RR:1.45; 95%CI:1.17–1.78), respectively) after extreme-cold (Fig. 2, Supplementary Table S2, Supplementary Figure S9).

**Fig. 2:**
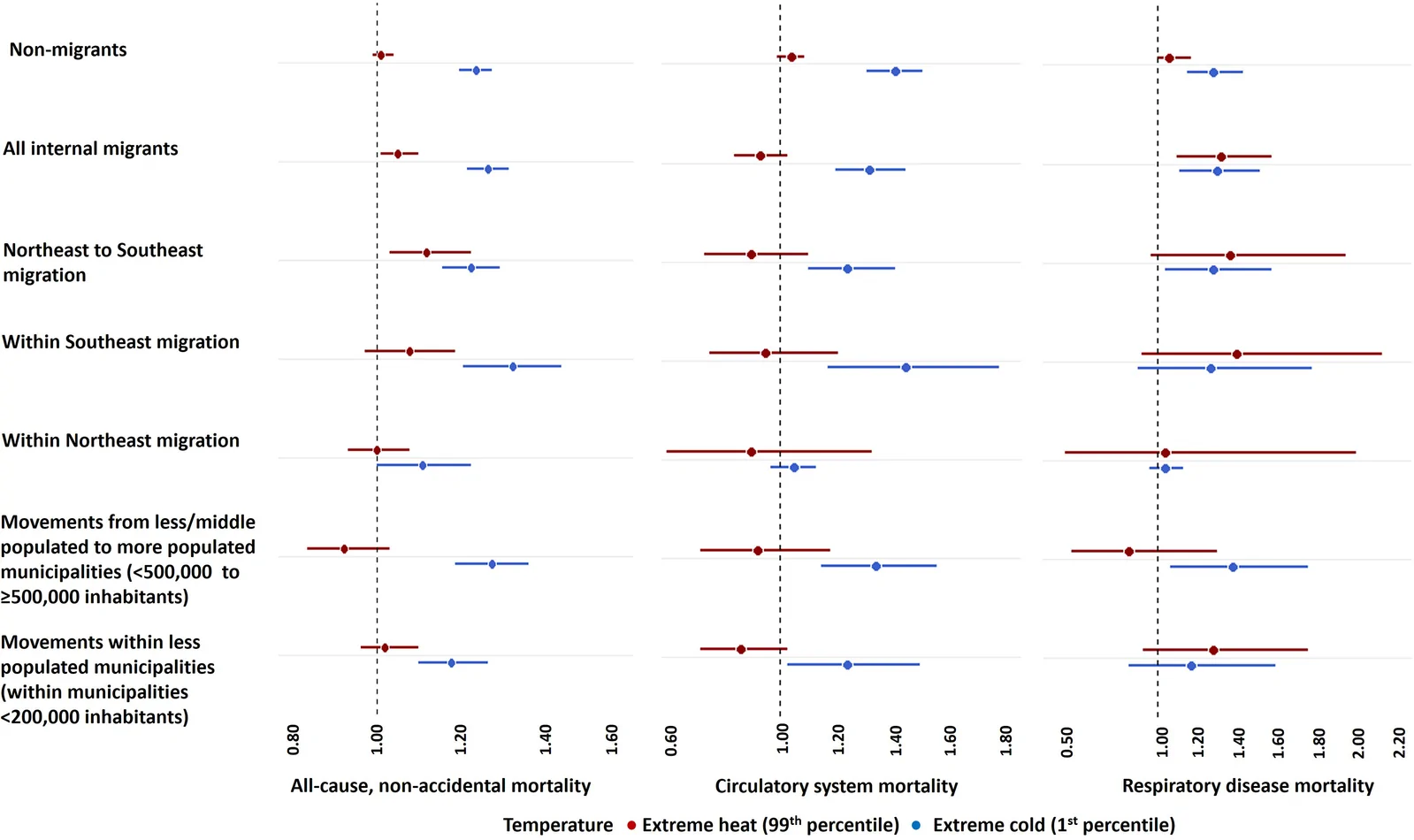
Relative risks (RR) and 95% confidence intervals (CI) of all-cause and cause-specific mortality associated with extreme ambient temperatures, cumulated over lag of 0–20 days, for non-migrants, all internal migrants, and key subgroups of internal migrants categorised by different migration routes. The horizontal axis shows the cumulative relative risk of mortality over lag days. Red colour indicates RR and 95% CI associated with extreme-heat, while blue colour indicates RR and 95% CI for extreme-cold.

Cold-related mortality tended to increase with migration to more populated municipalities, over 0–20 days. Over 0–6 days, migrants who moved to more populated areas had a higher risk of circulatory mortality after extreme-heat (Fig. 2, Supplementary Tables S1 and S2 and Supplementary Figures S7–S9).

Over 0–20 days, almost all subgroups of internal migrants had a higher risk of all-cause, non-accidental mortality compared to non-migrants. This difference was particularly pronounced among illiterate migrants (RR:1.29; 95%CI:1.20–1.40) compared with the corresponding group of non-migrants (RR:1.12; 95%CI:1.04–1.21) after extreme-cold (Fig. 3, Supplementary Table S2, Supplementary Figure S12). For circulatory mortality, extreme-cold was more strongly associated with mortality than extreme-heat at both short- and long-lags. Non-migrants were slightly more vulnerable than internal migrants for circulatory mortality, particularly non-migrants living in houses made by wood and rammed earth (RR:1.62; 95%CI:1.35–1.94) after extreme-cold, which was not associated with significant risk in internal migrants (Fig. 3, Supplementary Tables S1 and S2, Supplementary Figures S12 and S16). Heat-related respiratory disease mortality was stronger among internal-migrants than non-migrants. Almost all internal migrant subgroups showed higher risks of respiratory disease mortality after extreme-heat compared with non-migrants, particularly among illiterate individuals (RR_Mig_(95%CI):1.84(1.25–2.71) vs. RR_Non-mig_:1.10(0.89–1.36)) (Fig. 3, Supplementary Table S2, Supplementary Figure S13). There were limited numbers for renal failure mortality in the cohort, so it was not possible to extract reliable estimates in stratified analysis (Supplementary Tables S1 and S2, Supplementary Figures S8, S9 and S13). Supplementary Figures S10 and S11 show the temperature–mortality curves from the stratified sensitivity analyses over the 0-6-day lag period. Supplementary Tables S3–S7 present summaries and heat maps for various migration routes across Brazilian regions, and population subcategories.

**Fig. 3:**
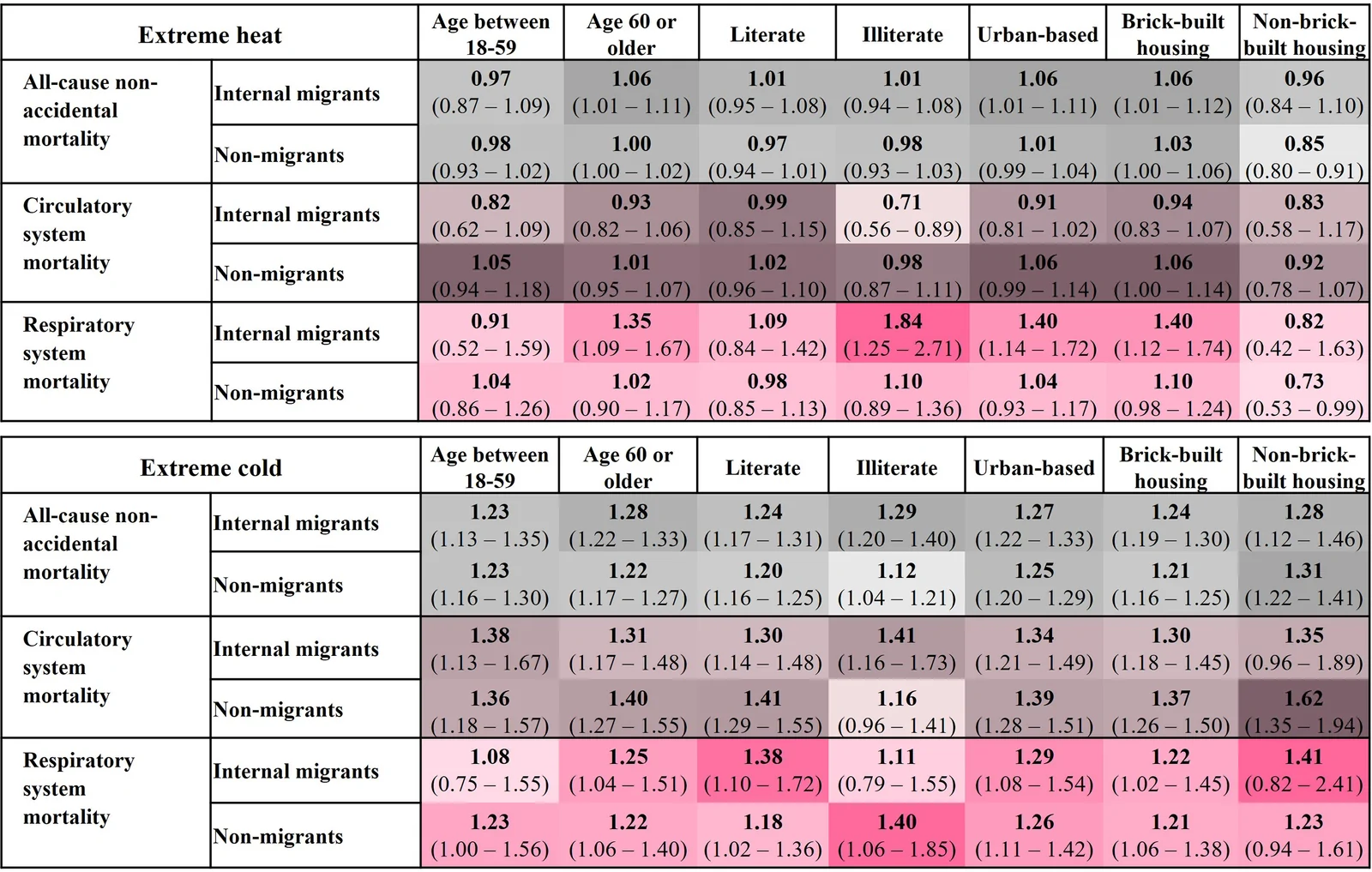
Heat map of cumulative relative risks (RR) and 95% confidence intervals (CI) of all-cause and cause-specific mortality associated with extreme-heat and extreme-cold, stratified by age (between 18 and 59, 60 or older), literacy (literate and illiterate), urbanicity and housing materials, over 0–20 days. Background shading is used to distinguish the three mortality outcomes (all-cause, circulatory, and respiratory mortality). Within each mortality outcome, bold colour indicates the higher RR between internal migrants and non-migrants.

Sensitivity analysis defining migration at the municipality level discovered findings consistent with the primary analysis with higher heat-related respiratory disease mortality risk in internal migrants (RR_Mig_(95%CI):1.38(1.30–1.47) vs. RR_Non-mig_:1.24(1.12–1.38), 0–6 days) and (RR_Mig_(95%CI):1.20(1.08–1.34) vs. RR_Non-mig_:1.00(0.93–1.08), 0–20 days) (Supplementary Tables S8–S9). We conducted another sensitivity analysis by using the median of each group as the reference point and discovered consistent findings of higher all-cause and heat-related respiratory mortality among internal migrants over 0–20 days (Supplementary Tables S10 and S11). We also analysed genitourinary system mortality and observed generally comparable heat-related mortality risks between internal migrants and non-migrants, although non-migrants exhibited slightly higher risks at the shorter lag period with reference to median (Supplementary Table S12). Additionally, we stratified the analyses by biome of the residency of death which showed consistent tendency to higher heat-related respiratory mortality in internal migrants (Supplementary Tables S1 and S2 and Supplementary Figures S14 and S15). In robustness checks of our model specifications, using an alternative knot placement (at the 10th and 90th percentiles) produced findings consistent with the primary analysis. Internal migrants continued to exhibit higher all-cause and heat-related respiratory mortality, but lower cold-related circulatory mortality than non-migrants over both the 0-6-day and 0-20-day lag periods (Supplementary Tables S13 and S14). By including migration as an interaction term, the point estimates showed consistent patterns with the main analysis, with higher extreme heat-related respiratory mortality among internal migrants compared with non-migrants, over both short and long lags. However, likelihood ratio tests were not indicative that mortality curves were modified by migration (Supplementary Tables S15 and S16).

## Discussion

In this study, we investigated the short-term associations between ambient temperature and all-cause and cause-specific mortality among migrants and non-migrants in Brazil which aligns with calls from WHO^6^ and the recent Belém health adaptation plan to include the voices of vulnerable populations.^8^ In our cohort, international and internal migrants were older than non-migrants, with a higher proportion living in urban areas. Both migrants and non-migrants represent socially vulnerable populations enrolled in CadÚnico, so large differences in relative risks would not necessarily be expected. However, migrants presented slightly higher temperature-related all-cause mortality in temperature extremes compared to non-migrants. Internal migrants had around 4% higher relative risk after extreme heat and 3% after extreme cold over 0–20 days lags than non-migrants (Extreme heat-related RR_Mig_(95%CI):1.05(1.01–1.10) & RR_Non-mig_(95%CI):1.01(0.99–1.04) and Extreme cold-related RR_Mig_(95%CI):1.27(1.22–1.32) & RR_Non-mig_(95%CI):1.24(1.20–1.28)). In addition, compared with non-migrants, internal migrants tended to have a 26% higher heat-related respiratory mortality risk but a 9% lower cold-related circulatory relative risk (Extreme heat-related respiratory RR_Mig_(95%CI):1.32(1.10–1.58) & RR_Non-mig_(95%CI):1.06(1.00–1.17) and Extreme cold-related circulatory RR_Mig_(95%CI):1.32(1.20–1.45) & RR_Non-mig_(95%CI):1.41(1.31–1.51)). However, between-group differences should be interpreted cautiously because confidence intervals overlapped substantially. Moreover, although our analysis is self-controlled, migrants and non-migrants may still differ in some demographic and socioeconomic characteristics.

Our findings are informative in environmental epidemiology, where exposures such as ambient temperature are nearly universal, individual effect sizes are often modest, and substantial heterogeneity exists in susceptibility. Our results showing that internal migrants might be potentially more vulnerable to extreme ambient temperatures, consistent with studies comparing international migrants with local-born populations. A 19-year study in Amman, Jordan emphasised the increased vulnerability of marginalised communities, particularly international migrants, to extreme weather conditions.^36^ Several studies have also reported increased vulnerability to extreme heat among migrant populations. Immigrants in Hong Kong, China,^37^ non-Kuwaitis in Kuwait^38^ and non-US citizens^39^ had a higher risk of heat-related mortality compared to non-migrants. In contrast, a study in France found minimal differences in temperature-mortality relationships between foreign-born and native-born populations.^40^ These studies did not address internal migrants. Studies in Nairobi's informal settlements reported weather-related mortality^41^ and years of life lost,^42^ as well as increased mortality among Nepali migrant workers in Qatar during hot periods.^43^ However, neither of these studies included comparisons with local non-migrant populations. By directly comparing internal migrants with non-migrants, our study suggests that migration status may modify vulnerability to temperature-related mortality.

Heat-related respiratory mortality tended to be higher among internal migrants, showing a 32% (RR_Mig_(95%CI):1.32(1.10–1.58)) increased risk at longer lags, whereas non-migrants exhibited a much weaker association (RR_Non-mig_(95%CI):1.06(1.00–1.17)). A review study found that climate change-related hazards such as flooding, drought, and extreme-heat contribute to respiratory diseases and premature mortality in African migrants.^44^ Heat exposure reduces lung function, causes airway injury, inflammation, and disruption of the microbiota.^45^ An umbrella review found strong evidence linking heat to increased respiratory mortality, particularly among socioeconomically vulnerable populations.^46^ We observed stronger associations among illiterate internal migrants, with an 84% higher respiratory mortality risk (RR_Mig_(95%CI):1.84(1.25–2.71)), while the magnitude of the association was smaller (10%) and not significant among non-migrants (RR_Non-mig_(95%CI):1.10(0.89–1.36)). Low educational attainment may directly increase vulnerability to extreme heat through lower health literacy, reduced risk awareness and limited adaptation of protective behaviours, potentially delaying healthcare-seeking and increase adverse health outcomes.^47^ Beyond these direct effects, low educational attainment is closely linked to socioeconomic disadvantage, which shapes migrants' living and working conditions after relocation. In Brazil, less educated internal migrants often reside in informal settlements with poor infrastructure and limited access to essential services, increasing exposure to overcrowding and various environmental hazards. They are also more likely to work in informal or unstable jobs with poor conditions and limited occupational protections.^12^ Migrants are more likely to reside in underdeveloped areas^11^, ^13^ where poor housing, overcrowding, limited cooling, and exposing to environmental hazards, dust, and pollution may amplify heat exposure and vulnerability.^48^, ^49^ These findings highlight targeted heat-adaptation strategies for vulnerable migrants, including improved housing and ventilation, access to cooling and shaded environments, occupational protections, early-warnings, and targeted primary healthcare outreach.

Although we expected internal migrants to be more susceptible to extreme temperatures for circulatory mortality, we did not find strong evidence for it. This may reflect emerging evidence from high-income countries of differential age-specific response to extreme temperature circulatory mortality.^50^ Another potential explanation is that, as our design did not control for competing risks of death, higher mortality at moderate temperatures for some specific causes could influence mortality risk at higher temperature by other causes, which can be differential for migrants and non-migrants. Higher respiratory mortality risks observed among migrants may highlight the different biological mechanisms and cumulative environmental or occupational exposures. We emphasise that these mechanisms could not be directly evaluated in the present study and therefore require further investigations.

Interestingly, heat-related respiratory risks were higher among residents of brick or masonry houses, housing material alone may not reflect thermal comfort or housing quality. In many Brazilian urban settings, poorly ventilated masonry dwellings with zinc or fibre-concrete roofs provide limited insulation, potentially increasing indoor temperatures during extreme heat events.^51^ Therefore, in urban areas where such housing predominates, the association may reflect dwelling thermal characteristics rather than better housing conditions. These suggestions are partially supported by the fact that our results indicate migration to more populated areas were associated with higher circulatory mortality risk from extreme heat in 0–6 days compared to moving to less populated areas. High population density may exacerbate heat exposure through heat retention and limited cooling; these geographic and climatic differences may also contribute. A multicounty study similarly found greater heat-related mortality in populated cities, associated with high air pollution and limited green infrastructure.^52^

Several limitations should be considered when interpreting these findings. International migrants were not captured adequately in the cohort, despite being a vulnerable group. Our migration definition was based on administrative records of state of birth, CadÚnico registration, and death, and therefore represents an operational measure of interstate migration. It does not capture the timing, duration, or multiple migration events over the life course, and some individuals may have been misclassified (like return migrants or those who migrated early in life). We adopted a state-level definition of migration to avoid capturing potential births that could have been registered in neighbouring municipalities rather than the true place of residence; therefore, some of internal municipal migration may have been misclassified. To address this, we conducted a sensitivity analysis using a municipality-level definition of migration. The small number of migrants in the dataset may have reduced statistical power, specifically in subgroup analyses. Potential exposure misclassification may arise from using municipality-level temperature data, which may not adequately capture individual-level exposure. Lack of information on individual-level mobility and access to cooling or heating devices may further limit estimate precision. National-level estimates may obscure important regional differences in temperature-mortality associations. Although population-weighted temperatures provide a better representation of average population exposure than simple spatial averages, some IGRs encompass substantial climatic variability. Consequently, assigning a single temperature estimate to all individuals within an IGR may have introduced non-differential exposure misclassification. Longer-lag periods in DLNMs applied within a space-time case-crossover design may introduce selection bias due to overlapping case and control exposure windows; however, sensitivity analysis using shorter lag (0–6 days) yielded findings consistent with the primary analysis, with internal migrants tending toward higher all-cause and respiratory mortality risks but lower circulatory mortality risks than non-migrants. Although our space-time-stratified case-crossover design inherently controls for long-term trends, seasonality, and day-of-week effects through its matching structure, residual serial correlation from short-term epidemic clustering or unmeasured day-specific factors cannot be fully excluded. In addition, residual confounding by unmeasured time-varying factors, such as respiratory infections, or other transient environmental exposures, may have influenced the estimated temperature–mortality associations. In addition, the study population represents socially vulnerable groups, which may limit the generalisability of the results. The magnitude of temperature-related mortality may have been more pronounced if compared with the general population of Brazil. Another limitation is that our cause-specific mortality analysis did not explicitly account for competing risks of death across different lags or temperature extremes, which can be different for different population subgroups, such as migrants. Therefore, future methodological research could inform new approaches for addressing competing risks within environmental epidemiology. In addition, formally assessments of temperature × migration interactions using the likelihood ratio test found mostly no evidence for effect modification by migration status. However, the test can only evaluate differences in the overall exposure-response function and not differences in the RR for temperature extremes. Therefore, a non-significant global interaction test does not necessarily exclude differences at specific exposure levels, including extreme cold and heat, which were the primary exposures of interest in this study. Finally, despite high linkage accuracy,^26^ it may include more complete records, introducing the possibility of selection bias in a case-crossover design.

### Conclusion

This study provides novel evidence suggesting that internal migrants in Brazil are more vulnerable to heat-related respiratory mortality, particularly among lower-educated people. Our findings underscore the need to prioritise migrants' health in heat protection strategies, including improvements in housing, working conditions and healthcare access, and targeted heat-health education and risk communication for lower-educated populations. Future studies should adopt qualitative approaches to better understand migrants' living and working conditions and their specific needs, to inform more effective migrant-sensitive climate adaptation policies.

## Contributors

**NF**: Conceptualisation, Data curation, Formal analysis, Methodology, Software, Visualisation, Writing original draft, and Review & editing.

**TRC:** Formal analysis, Methodology, Review & editing, and Access to and verification of the underlying data.

**LVJ:** Methodology, Review & editing.

**DJDN:** Data curation and software.

**IHS:** Methodology, Review & editing.

**ShH:** Methodology, Review & editing.

**MLB:** Project administration and resources, Review & editing.

**JMP:** Conceptualisation, Methodology, Funding acquisition, Project administration, Supervision, Review & editing, and Access to and verification of the underlying data.

## Data sharing statement

All data supporting this study were obtained from CIDACS. The data were licenced for exclusive use in the present study and, due to privacy regulations under Brazilian law and the requirements of the relevant Ethics Committee, are not publicly available. Upon request, controlled access to the data may be considered by Ethics Committee.

## AI use statement

ChatGPT (OpenAI) was used exclusively for language editing and grammar correction. No AI tools were used for data analysis, interpretation, or generation of scientific content.

## Declaration of interests

We declare no competing interests.

## References

[1] Walinski A., Sander J., Gerlinger G., Clemens V., Meyer-Lindenberg A., Heinz A. (2023). The effects of climate change on mental health. Dtsch Arztebl Int.

[2] Cortes T.R., Pescarini J.M., Ranzani O.T. (2025). Ambient temperature and non-accidental mortality: a nationwide space–time-stratified case-crossover study within the 100 million Brazilian cohort. Environ Int.

[3] Deivanayagam T.A., English S., Hickel J. (2023). Envisioning environmental equity: climate change, health, and racial justice. Lancet.

[4] Leal Filho W., Matandirotya N.R., Lütz J.M. (2021). Impacts of climate change to African indigenous communities and examples of adaptation responses. Nat Commun.

[5] Liu J., Ho H.C., Tong S. (2025). Heatwaves and immigrant mortality: an assessment of vulnerability and temporal variation. Environ Sci Technol.

[6] World Health Organization Health system strengthening interventions to improve the health of displaced and migrant populations in the context of climate change. https://environmentalmigration.iom.int/sites/g/files/tmzbdl1411/files/documents/2025-09/9789240112452-eng.pdf.

[7] United Nations Act now: migrant inclusion in climate action is an obligation, not an option. https://migrationnetwork.un.org/statements/act-now-migrant-inclusion-climate-action-obligation-not-option.

[8] WHO-COP30-Amazonia (2025). The Belém health action plan for the adaptation of the health sector to climate change. https://cdn.who.int/media/docs/default-source/climate-change/en---belem-action-plan.pdf.

[9] Brance K., Chatzimpyros V., Bentall R.P. (2024). Social identity, mental health and the experience of migration. Br J Soc Psychol.

[10] Jia J., Nie X.-F., Ke L., Liu B., Wang W.-R. (2022). Mental health and its influencing factors among immigrants with chronic diseases in China. J Immigr Minor Health.

[11] Ribeiro A.C.B.M., Tai S.H.T. (2023). Migrant networks, regional development and internal migration flows in Brazil. EconomiA.

[12] The UN Refugee Agency ILO Livelihoods for migrants & refugees. https://www.ilo.org/publications/livelihoods-migrants-and-refugees-brazil.

[13] Tacoli C., Mcgranahan G., Satterthwaite D. (2015). World Migration Report Urbanization, Rural-Urban Migration and Urban Poverty Background Paper Background Paper Migrants and Cities: New Partnerships to Manage Mobility.

[14] World Health Organization (2022). Refugee and migrant health. https://www.who.int/news-room/fact-sheets/detail/refugee-and-migrant-health.

[15] IBGE (Instituto Brasileiro de Geografia e Estatística) Censo Demográfico 2022, Fecundidade e migração - Resultados preliminares da amostra, [IBGE - Brazilian Institute of Geography and Statistics, Census 2022, Fertility and migration – Preliminary results from the sample]. https://biblioteca.ibge.gov.br/index.php/biblioteca-catalogo?view=detalhes&id=2102187.

[16] da Cunha J.M.P. (2019). Paths of Inequality in Brazil.

[17] Sahota G.S. (1968). An economic analysis of internal migration in Brazil. J Polit Econ.

[18] de Souza Ferreira Filho J.B., Horridge M., Madden J., Shibusawa H., Higano Y. (2020). Environmental Economics and Computable General Equilibrium Analysis. New Frontiers in Regional Science: Asian Perspectives, vol 41.

[19] United Nations Human Rights Migrants in vulnerable situations | OHCHR. https://www.ohchr.org/en/migration/migrants-vulnerable-situations.

[20] Instituto Nacional de Meteorologia - INMET [National Institute of Meteorology]. https://portal.inmet.gov.br/servicos/bdmep-dados-hist%C3%B3ricos.

[21] de Moraes S.L., Almendra R., Barrozo L.V. (2022). Impact of heat waves and cold spells on cause-specific mortality in the city of São Paulo, Brazil. Int J Hyg Environ Health.

[22] Collazo S., Barriopedro D., García-Herrera R., Beguería S. (2025).

[23] Barreto M.L., Ichihara M.Y., Pescarini J.M. (2022). Cohort profile: the 100 million Brazilian cohort. Int J Epidemiol.

[24] Wu Y., Li S., Guo Y. (2021). Space-time-stratified case-crossover design in environmental epidemiology study. Health Data Sci.

[25] Rebouças P., Alves F.J., Ferreira A. (2025). [Quality evaluation of Brazilian Mortality Information System (SIM): a scoping review]. Cien Saude Colet.

[26] Barbosa G.C.G., Ali M.S., Araujo B. (2020). CIDACS-RL: a novel indexing search and scoring-based record linkage system for huge datasets with high accuracy and scalability. BMC Med Inform Decis Mak.

[27] Xavier A.C., Scanlon B.R., King C.W., Alves A.I. (2022). New improved Brazilian daily weather gridded data (1961–2020). Int J Climatol.

[28] Instituto Brasileiro de Geografia e Estatística (2017). Instituto Brasileiro de Geografia e Estatística. Divisão regional do Brasil em regiões geográficas imediatas e regiões geográficas intermediárias: 2017 | IBGE [IBGE - Brazilian Institute of Geography and Statistics, Regional Division of Brazil in immediate and intermediate geographical regions: 2017]. https://biblioteca.ibge.gov.br/visualizacao/livros/liv100600.pdf.

[29] Xavier A.C., Scanlon B.R., King C.W., Alves A.I. (2022). New improved Brazilian daily weather gridded data (1961–2020). Int J Climatol.

[30] Xavier A.C., King C.W., Scanlon B.R. (2016). Daily gridded meteorological variables in Brazil (1980–2013). Int J Climatol.

[31] WorldPop Population density. https://hub.worldpop.org/geodata/summary?id=39835.

[32] Gasparrini A. (2011). Distributed lag linear and non-linear models in R: the package dlnm. J Stat Softw.

[33] Fu S.H., Gasparrini A., Rodriguez P.S., Jha P. (2018). Mortality attributable to hot and cold ambient temperatures in India: a nationally representative case-crossover study. PLoS Med.

[34] Alahmad B., Khraishah H., Royé D. (2023). Associations between extreme temperatures and cardiovascular cause-specific mortality: results from 27 countries. Circulation.

[35] Alahmad B., Shakarchi A.F., Khraishah H. (2020). Extreme temperatures and mortality in Kuwait: who is vulnerable?. Sci Total Environ.

[36] Kysely J. (2004). Mortality and displaced mortality during heat waves in the Czech Republic. Int J Biometeorol.

[37] Liu J., Ho H.C., Tong S. (2025). Heatwaves and immigrant mortality: an assessment of vulnerability and temporal variation. Environ Sci Technol.

[38] Revich B., Shaposhnikov D. (2022). The influence of heat and cold waves on mortality in Russian subarctic cities with varying climates. Int J Biometeorol.

[39] Taylor E.V., Vaidyanathan A., Flanders W.D., Murphy M., Spencer M., Noe R.S. (2018). Differences in heat-related mortality by citizenship status: United States, 2005–2014. Am J Public Health.

[40] Mercereau L., Todd N., Rey G., Valleron A.-J. (2017). Comparison of the temperature-mortality relationship in foreign born and native born died in France between 2000 and 2009. Int J Biometeorol.

[41] Egondi T., Kyobutungi C., Kovats S., Muindi K., Ettarh R., Rocklöv J. (2012). Time-series analysis of weather and mortality patterns in Nairobi's informal settlements. Glob Health Action.

[42] Egondi T., Kyobutungi C., Rocklöv J. (2015). Temperature variation and heat wave and cold spell impacts on years of life lost among the urban poor population of Nairobi, Kenya. Int J Environ Res Public Health.

[43] Pradhan B., Kjellstrom T., Atar D. (2019). Heat stress impacts on cardiac mortality in Nepali migrant workers in Qatar. Cardiology.

[44] Sanni O., Salami B., Oluwasina F., Ojo F., Kennedy M. (2022). Climate change and African migrant health. Int J Environ Res Public Health.

[45] Zhu Y., Jiang Y., Du X. (2025). Heat exposure changes lung function, biomarkers of airway injury, and airway microbiota: a randomized, crossover trial. Environ Health.

[46] Zhu Z., Ji B., Tian J., Yin P. (2025). Heat exposure and respiratory diseases health outcomes: an umbrella review. Sci Total Environ.

[47] VanderMolen K., Hatchett B. (2024).

[48] Taylor J., Symonds P., Wilkinson P. (2018). Estimating the influence of housing energy efficiency and overheating adaptations on heat-related mortality in the West Midlands, UK. Atmosphere.

[49] Cole R., Simpson C.H., Ferguson L. (2024). A population-level framework to estimate unequal exposure to indoor heat and air pollution. Build Cities.

[50] Achebak H., Devolder D., Ballester J. (2019). Trends in temperature-related age-specific and sex-specific mortality from cardiovascular diseases in Spain: a national time-series analysis. Lancet Planet Health.

[51] Garcia D.R., Mendes V.F., Moraes C.F., Cabral M.M.Q., Silva I.B.A.R., Mendes J.C. (2026). Climate change, thermal comfort, and low-income residences: insights from post-occupancy evaluations of concrete wall buildings in Brazil. Sustain Energy Technol Assessments.

[52] Sera F., Armstrong B., Tobias A. (2019). How urban characteristics affect vulnerability to heat and cold: a multi-country analysis. Int J Epidemiol.

