# Supplementary material for: Differential mortality risks associated with ambient temperature among migrants and non-migrants: a space-time-stratified case-crossover study of the 100 Million Brazilian Cohort (2000–2020)
Source: Lancet Reg Health Am. 2026 Oct 27;66:101687. doi: 10.1016/j.lana.2026.101687 (PMC13734820; doi:10.1016/j.lana.2026.101687)
Supplement: Supplementary file 1 [file mmc1.pdf]

Supplementary material

Title: ***Differential Mortality Risks Associated with Ambient Temperature Among Migrants and Non-migrants: A Space-Time-Stratified Case-Crossover Study of the 100 Million Brazilian Cohort (2000-2020***

List of Contents

- **Supplementary Figure 1.** The conceptual framework of migration consequences in the destination place
- **Supplementary Figure 2.** Flowchart of study participants selection
- **Supplementary Figure 3**. Relative risks of all-cause and cause-specific mortality associated with extreme ambient temperature cumulated over 6-day and 20-day lag periods, among international migrants.
- **Supplementary Table 1.** Relative risks (RR) and 95% confidence intervals (CI) for all-cause and cause-specific mortality associated with extreme ambient temperatures, cumulated over 6 days.
- **Supplementary Table 2.** Relative risks and 95% confidence intervals of all-cause and cause-specific mortality associated with extreme ambient temperatures, cumulated over 20 days.
- **Supplementary Figure 4.** Exposure–response association between ambient temperature (°C) and all-cause and cause-specific mortality among non-migrants and internal migrants, over 0-6 days
- **Supplementary Figure 5.** Exposure–lag–response curves for all cause and cause specific mortality among non-migrants and internal migrants across lag 0-20 days.
- **Supplementary Figure 6.** Lag–response curves for all-cause and cause-specific mortality among non-migrants and internal migrants across lag 0–20 days
- **Supplementary Figure 7.** Relative risks (RR) and 95% confidence intervals (CI) of all-cause and cause-specific mortality associated with extreme ambient temperatures, cumulated over lag of 0–6 days, for non-migrants, all internal migrants, and subgroups of internal migrants categorized by different migration routes.
- **Supplementary Figure 8.** Relative risks of all-cause and cause-specific mortality associated with extreme ambient temperature cumulated over 6-day, among non-migrants and different sub-categories of migrants
- **Supplementary Figure 9.** Relative risks of all-cause and cause-specific mortality associated with extreme ambient temperature cumulated over 20-day, among non-migrants and different sub-categories of migrants.
- **Supplementary Figure 10.** Relative risks of **all-cause non-accidental mortality and circulatory system mortality** associated with extreme ambient temperature cumulated over 0-6-day lag periods for different individual characteristics and housing conditions.
- **Supplementary Figure 11.** Relative risks of **respiratory system mortality** and **renal failure mortality** associated with extreme ambient temperature cumulated over 6-day lag periods for different individual characteristics and housing conditions.
- **Supplementary Figure 12.** Relative risks of **all-cause non-accidental mortality and circulatory system mortality** associated with extreme ambient temperature cumulated over 0-20-day lag periods for different individual characteristics and housing conditions.
- **Supplementary Figure 13.** Relative risks of **respiratory system mortality** and **renal failure mortality** associated with extreme ambient temperature cumulated over 0-20-day lag periods for different individual characteristics and housing conditions.
- **Supplementary Figure 14.** Relative risks of **all-cause and cause-specific mortality** associated with extreme ambient temperature cumulated over 0-6-day lag periods by biome of the place of death.
- **Supplementary Figure 15.** Relative risks of **all-cause and cause-specific mortality** associated with extreme ambient temperature cumulated over 0-20-day lag periods by biome of the place of death.
- **Supplementary Figure 16.** Heat map of relative risks (RR) and 95% confidence intervals (CI) of all-cause and cause-specific mortality associated with extreme-heat and extreme-cold, cumulated over 0-6 days in stratified modelling for age (between 18 and 59, 60 or older), literacy (literate and illiterate), urbanicity and housing materials.
- **Supplementary Table 3.** Summary of Origin and Destination Regions for Internal Migrants
- **Supplementary Table 4.** Heat map of the region matrix of origin–destination pairs for internal migrants
- **Supplementary Table 5.** Heat map of the region's matrix of origin–destination pairs across different migration routes
- **Supplementary Table 6.** Heat map of the population matrix of origin–destination pairs for internal migrants
- **Supplementary Table 7.** Heat map of the population matrix of origin–destination pairs across different migration time
- **Supplementary Table 8.** Relative risks and 95% confidence intervals of all-cause and cause-specific mortality associated with extreme ambient temperatures, cumulated over 0-6 days.
- **Supplementary Table 9.** Relative risks and 95% confidence intervals of all-cause and cause-specific mortality associated with extreme ambient temperatures, cumulated over 0-20 days.
- **Supplementary Table 10**. Relative risks and 95% confidence intervals of all-cause and cause-specific mortality associated with extreme ambient temperatures, cumulated over 0-6 days.
- **Supplementary Table 11**. Relative risks and 95% confidence intervals of all-cause and cause-specific mortality associated with extreme ambient temperatures, cumulated over 0-20 days.

- **Supplementary Table 12.** Relative risks and 95% confidence intervals of genitourinary system mortality associated with extreme ambient temperatures, cumulated over lag periods, comparing to both minimum mortality temperature and median.
- **Supplementary Table 13.** Relative risks and 95% confidence intervals of all-cause and cause-specific mortality associated with extreme ambient temperatures, cumulated over 0-6 days, using a different knot placement (p10 and p90) in the temperature cross-basis.
- **Supplementary Table 14.** Relative risks and 95% confidence intervals of all-cause and cause-specific mortality associated with extreme ambient temperatures, cumulated over 0-20 days, using a different knot placement (p10 and p90) in the temperature cross-basis.
- **Supplementary Table 15.** Relative risks and 95% confidence intervals of all-cause and cause-specific mortality associated with extreme ambient temperatures, cumulated over 0-6 days, by migration status, using interaction terms between temperature cross-basis and migration status
- **Supplementary Table 16.** Relative risks and 95% confidence intervals of all-cause and cause-specific mortality associated with extreme ambient temperatures, cumulated over 0-20 days, by migration status, using interaction terms between temperature cross-basis and migration status.

Supplementary Figure 1 shows a conceptual framework which provides a simplified representation of migration outcomes, illustrating the possible pathways linking migration to its consequences and the resulting increased burden of morbidity and mortality. The arrows denoting potential mechanisms underlying health outcomes among migrants.

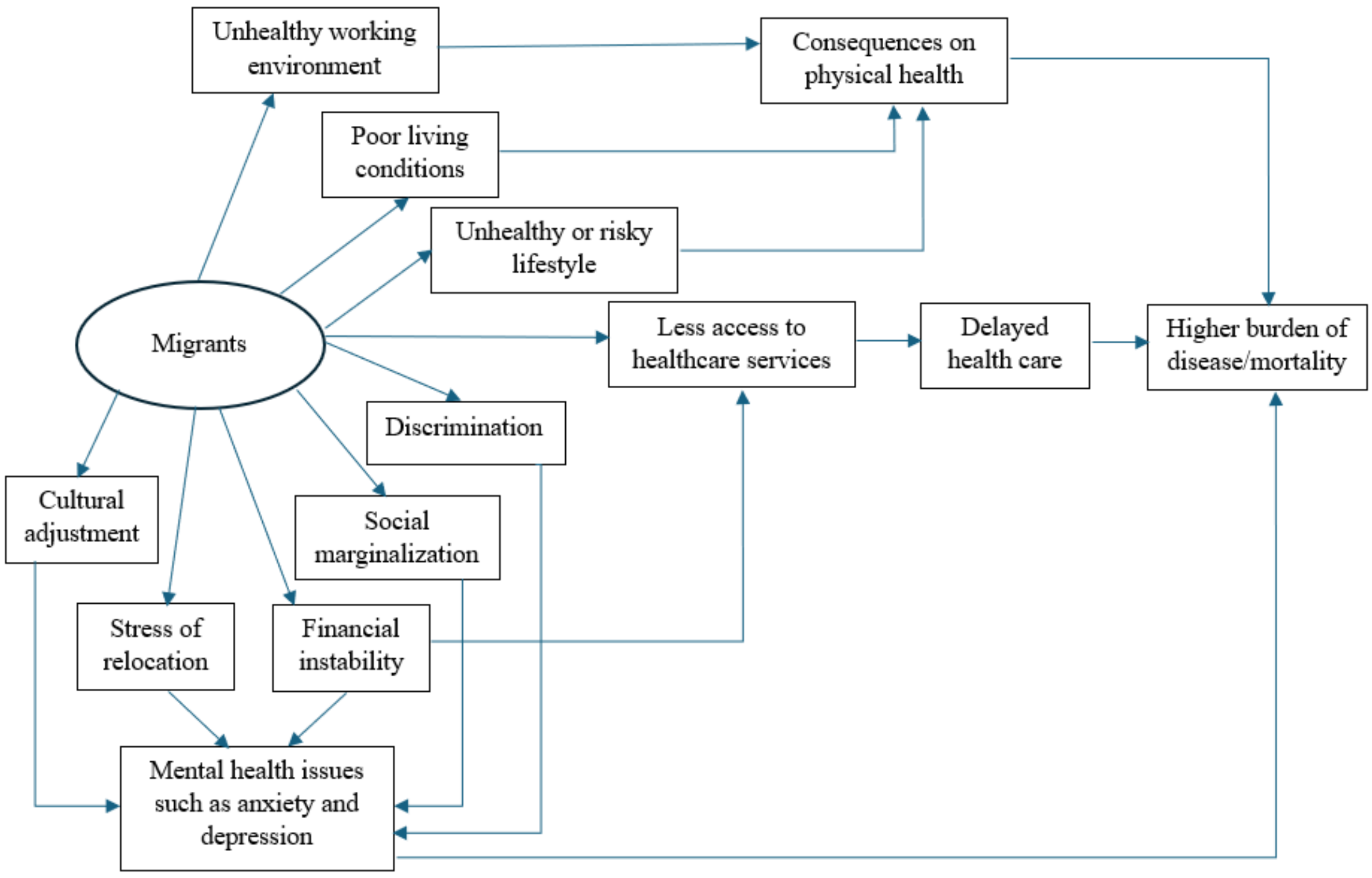

Supplementary Figure 1. The conceptual framework of migration consequences in the destination place

We analysed 4,935,541 deaths that occurred among the 100 Million Brazilian Cohort between 2000 and 2020 (Supplementary Figure 2). We excluded cases with missing values for birthplace, registration place, or death place (130,773,654 cases), individuals under 18 years (291,336), over 110 years at death (496), and those with invalid municipality-of-death codes after merging with IGR codes (5,522).

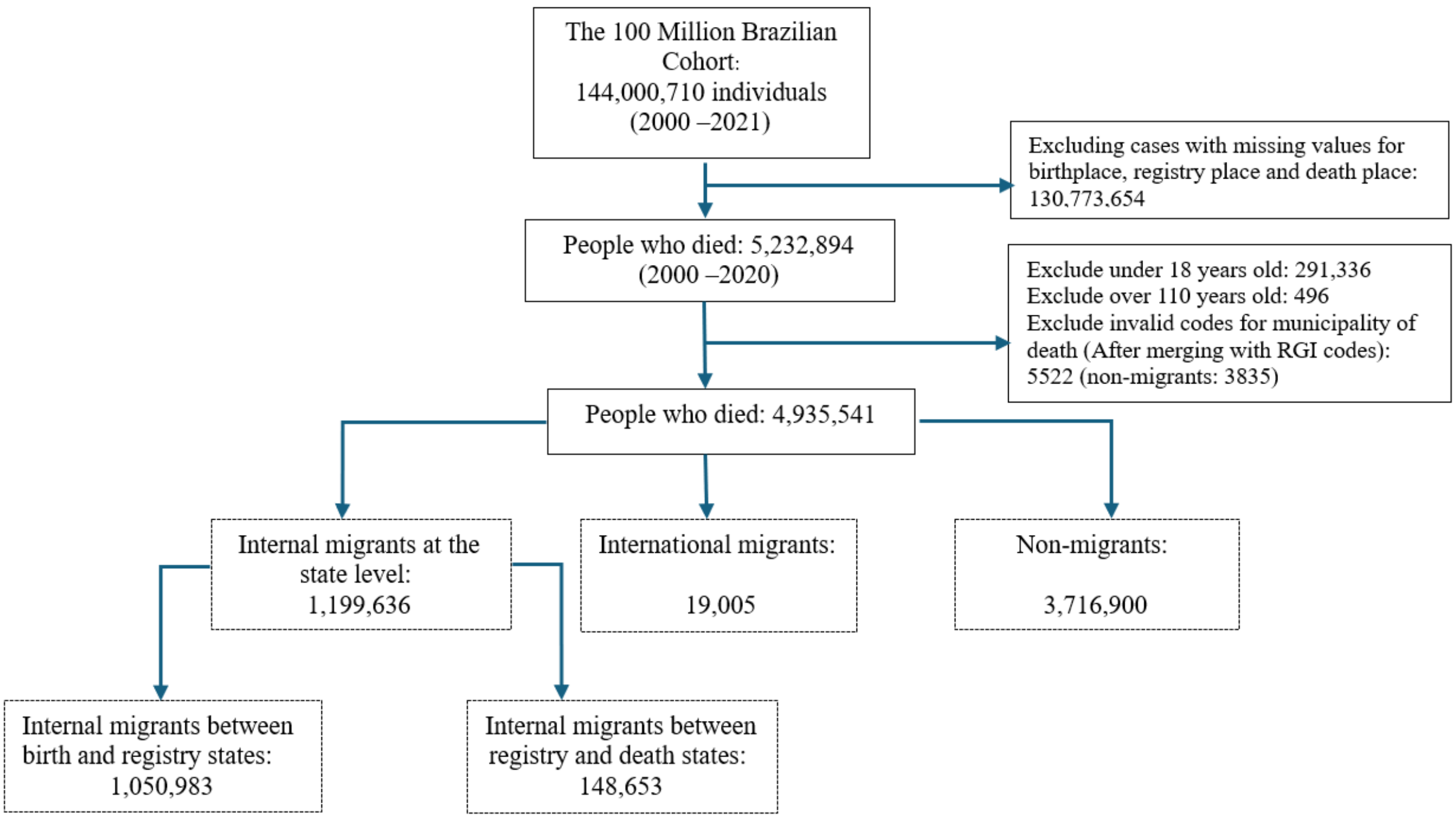

Supplementary Figure 2. Flowchart of study participants selection

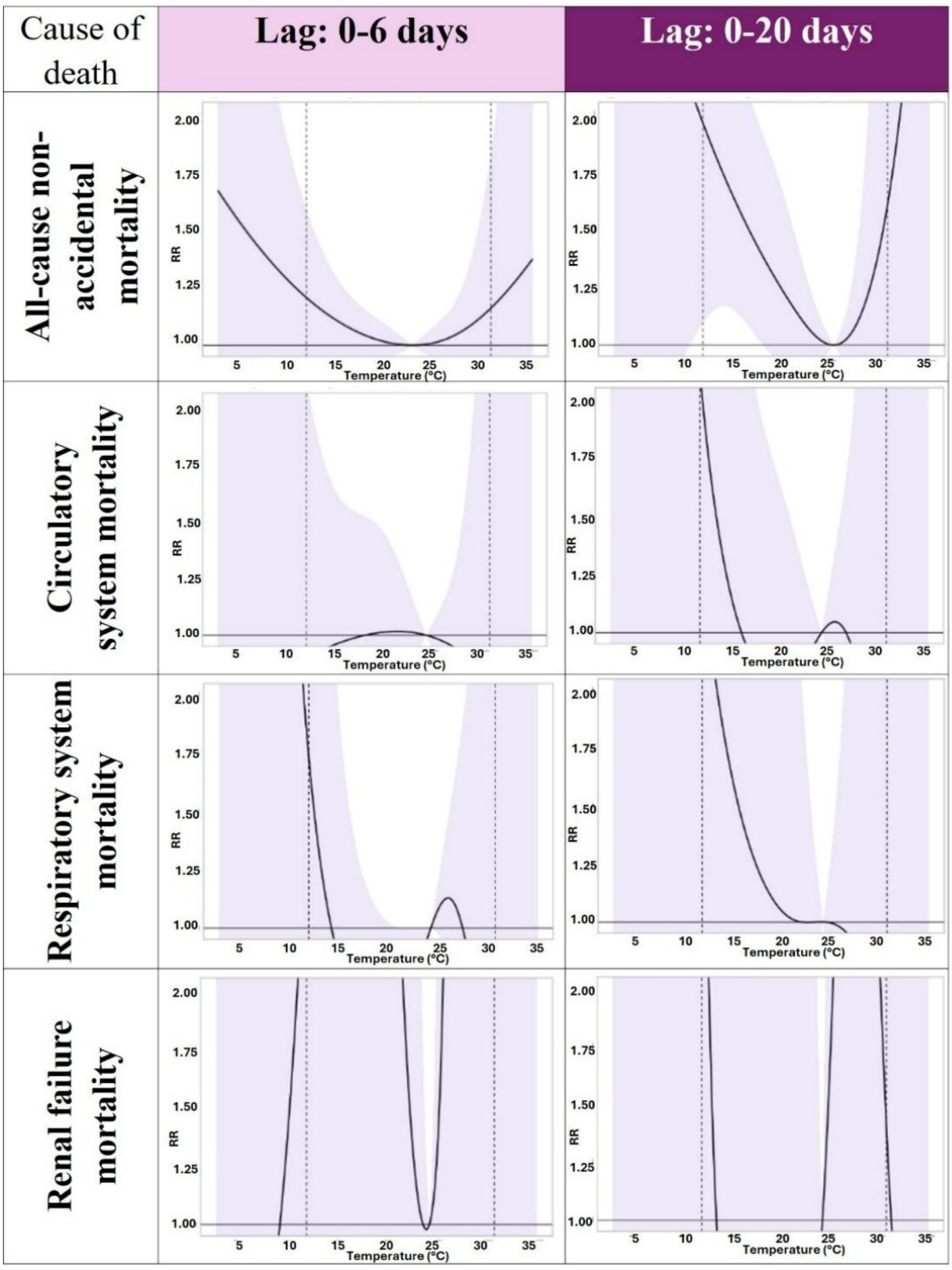

Supplementary Figure 3. Relative risks of **all-cause and cause-specific mortality** associated with extreme ambient temperature cumulated over 6-day and 20-day lag periods, among international migrants. Dotted vertical lines show the 0.01 and 0.99 temperature percentiles.

Supplementary Table 1. Relative risks (RR) and 95% confidence intervals (CI) for all-cause and cause-specific mortality associated with extreme ambient temperatures, cumulated over 6 days, across different migration subgroups.

Cause of death	Model/ Population (Lag: 0-6 days)	Migration Status	MMT	Median	$RR_{Cold\ (1st\ percentile)}$ (95% CI)	$RR_{Heat\ (99th\ percentile)}$ (95% CI)
**All-cause, non-accidental mortality**	**All**	Internal migrants	23.7	25.4	1.11(1.09 – 1.13)	1.15 (1.12 – 1.17)
		International migrants	22.6		1.21 (0.92 – 1.58)	1.16 (0.74 – 1.82)
		Non-migrants	24.2	25.2	1.10 (1.08 – 1.12)	1.12 (1.10 – 1.14)
	**Literate**	Internal migrants	23.3	25.3	1.07 (1.04 – 1.11)	1.11(1.08 – 1.15)
		Non-migrants	24	25	1.07 (1.05 – 1.10)	1.09 (1.07 – 1.11)
	**Illiterate**	Internal migrants	24.3	25.6	1.14 (1.10 – 1.18)	1.12 (1.07 – 1.17)
		Non-migrants	25.2	25.6	1.11 (1.06 – 1.15)	1.07 (1.04 – 1.10)
	**Urban-based**	Internal migrants	23.4	25.3	1.10 (1.07 – 1.12)	1.15 (1.12 – 1.18)
		Non-migrants	23.8	25	1.09 (1.07 – 1.11)	1.13 (1.11 – 1.15)
	**Brick or masonry housing material**	Internal migrants	23.5	25.2	1.09 (1.07 – 1.11)	1.17 (1.14 – 1.20)
		Non-migrants	24.5	25	1.09 (1.07 – 1.11)	1.14 (1.12 – 1.15)
	**Other housing material (wood, rammed earth, others)**	Internal migrants	35	26*	1.09 (1.02 – 1.16)	0.99 (0.93 – 1.07)
		Non-migrants	35.7	25.7*	1.12 (1.08 – 1.17)	0.96 (0.93 – 0.99)
	**Age between 18-59**	Internal migrants	23	25.4	1.10 (1.05 – 1.14)	1.01 (0.95 – 1.08)
		Non-migrants	25.5	25.3	1.12 (1.08 – 1.15)	1.02 (1.00 – 1.05)
	**Age 60 or older**	Internal migrants	23.7	25.4	1.09 (1.07 – 1.12)	1.16 (1.13 – 1.19)
		Non-migrants	23.7	25.1	1.08 (1.06 – 1.10)	1.14 (1.12 – 1.16)
	**Moving from less populated area to less populated area (Less than 200,000 to Less than 200,00)**	Internal migrants	24.4	25.5	1.08 (1.04 – 1.12)	1.10 (1.06 – 1.14)
	**Moving from less populated area to more populated area (Less than 500,000 to More than 500,00)**	Internal migrants	23.1	24.7	1.12 (1.08 – 1.16)	1.15 (1.08 – 1.22)
	**Biome of death place: Amazon Forest**	Internal migrants	10.5	27.6*	1.04 (0.97 -71.10)	0.95 (0.89 – 1.01)
		Non-migrants	33.4	27.9*	1.13 (1.07 – 1.20)	0.92 (0.88 – 0.97)
	**Biome of death place: Caatinga**	Internal migrants	16	26.7*	1.05 (0.89 – 1.25)	1.26 (1.13 – 1.40)
		Non-migrants	26.6	26.6	1.09 (1.05 – 1.14)	1.08 (1.05 – 1.12)
	**Biome of death place: Cerrado**	Internal migrants	25.4	25.8	1.09 (1.03 – 1.15)	1.23 (1.17 – 1.30)
		Non-migrants	23.5	25.6	1.07 (1.03 – 1.10)	1.19 (1.15 – 1.24)
	**Biome of death place: Atlantic Forest**	Internal migrants	23.3	23.5	1.14 (1.10 – 1.17)	1.13 (1.10 – 1.16)
		Non-migrants	23.6	23.4	1.11 (1.08 – 1.13)	1.10 (1.08 – 1.12)
	**Moving from Northeast region to Northeast region**	Internal migrants	5	26.9*	1.01 (1.00 – 1.02)	1.15 (1.04 – 1.27)
	**Moving from Southeast region to Southeast region**	Internal migrants	24.2	23.2	1.12 (1.07 – 1.17)	1.10 (1.04 – 1.16)
	**Moving from Northeast region to Southeast region**	Internal migrants	22.7	23.9	1.08 (1.05 – 1.12)	1.15 (1.09 – 1.20)
**Circulatory system mortality**	**All**	Internal migrants	24.8	25.4	1.16 (1.11 – 1.22)	1.11(1.05 – 1.18)
		Non-migrants	25	25.2	1.21 (1.16 – 1.26)	1.11 (1.07 – 1.14)
	**Literate**	Internal migrants	24.8	25.3	1.13 (1.06 – 1.21)	1.11 (1.02 – 1.20)
		Non-migrants	24.9	25	1.22 (1.16 – 1.28)	1.09 (1.04 – 1.14)
	**Illiterate**	Internal migrants	35.7	25.6*	1.21 (1.09 – 1.33)	1.01 (0.89 – 1.14)
		Non-migrants	26	25.6	1.23 (1.11 – 1.36)	1.06 (1.00 – 1.13)
	**Urban-based**	Internal migrants	24.6	25.3	1.15 (1.10 – 1.22)	1.12 (1.05 – 1.19)
		Non-migrants	24.8	25	1.22 (1.17 – 1.27)	1.12 (1.07 – 1.16)
	**Brick or masonry housing material**	Internal migrants	24	25.2	1.14 (1.08 – 1.20)	1.11 (1.03 – 1.19)
		Non-migrants	25.2	25	1.22 (1.17 – 1.28)	1.13 (1.08 – 1.17)
	**Other housing material (wood, rammed earth, others)**	Internal migrants	35	26*	1.21 (1.02 – 1.42)	0.95 (0.79 – 1.15)
		Non-migrants	26.8	25.7	1.25 (1.12 – 1.38)	1.01 (0.92 – 1.10)
	**Age between 18-59**	Internal migrants	26	25.4	1.24 (1.12 – 1.38)	1.02 (0.88 – 1.18)
		Non-migrants	27.2	25.3	1.27 (1.18 – 1.37)	1.02 (1.00 – 1.09)
	**Age 60 or older**	Internal migrants	24.5	25.4	1.13 (1.07 – 1.19)	1.09 (1.02 – 1.17)
		Non-migrants	24.4	25.1	1.22 (1.16 – 1.28)	1.13 (1.08 – 1.18)
	**Moving from less populated area to less populated area (Less than 200,000 to Less than 200,00)**	Internal migrants	35	25.5*	1.13 (1.03 – 1.24)	1.00 (0.91 – 1.10)
	**Moving from less populated area to more populated area (Less than 500,000 to More than 500,00)**	Internal migrants	24.6	24.7	1.18 (1.09 – 1.28)	1.21 (1.05 – 1.39)
	**Biome of death place: Amazon Forest**	Internal migrants	10.5	27.6*	1.03 (0.89 – 1.20)	1.05 (0.90 – 1.24)
		Non-migrants	11.7	27.9*	1.05 (0.92 – 1.21)	1.06 (0.94 – 1.19)
	**Biome of death place: Caatinga**	Internal migrants	26.8	26.7	1.52 (0.97 – 2.38)	1.19 (0.91 – 1.57)
		Non-migrants	27.4	26.6	1.08 (0.99 – 1.19)	1.03 (0.96 – 1.10)
	**Biome of death place: Cerrado**	Internal migrants	26.7	25.8	1.09 (0.96 – 1.23)	1.15 (1.01 – 1.31)
		Non-migrants	26.1	25.6	1.15 (1.05 – 1.25)	1.16 (1.06 – 1.26)
	**Biome of death place: Atlantic Forest**	Internal migrants	24.2	23.5	1.20 (1.13 – 1.28)	1.08 (1.02 – 1.14)
		Non-migrants	24.1	23.4	1.21 (1.15 – 1.28)	1.09 (1.05 – 1.13)
	**Moving from Northeast region to Northeast region**	Internal migrants	27.7	26.9	1.02 (0.99 – 1.06)	1.21 (0.97 – 1.50)
	**Moving from Southeast region to Southeast region**	Internal migrants	25.3	23.2	1.19 (1.07 – 1.33)	1.07 (0.94 – 1.20)
	**Moving from Northeast region to Southeast region**	Internal migrants	23.5	23.9	1.15 (1.08 – 1.22)	1.05 (0.94 – 1.18)
**Respiratory disease mortality**	**All**	Internal migrants	19.4	25.4	1.09 (1.01 – 1.16)	1.37 (1.24 – 1.52)
		Non-migrants	19.4	25.2	1.04 (0.99 – 1.10)	1.29 (1.21 – 1.37)
	**Literate**	Internal migrants	18.6	25.3	1.10 (1.01 – 1.21)	1.33 (1.15 – 1.54)
		Non-migrants	14.6	25	1.00 (0.97 – 1.04)	1.27 (1.16 – 1.39)
	**Illiterate**	Internal migrants	3.2	25.6*	1.00 (0.85 – 1.17)	1.52 (1.23 – 1.87)
		Non-migrants	19.4	25.6	1.02 (0.91 – 1.15)	1.23 (1.07 – 1.40)
	**Urban-based**	Internal migrants	18.7	25.3	1.06 (1.00 – 1.14)	1.35 (1.21 – 1.51)

		Non-migrants	18.9	25	1.03 (0.98 – 1.09)	1.25 (1.17 – 1.34)
	**Brick or masonry housing material**	Internal migrants	19.2	25.2	1.06 (0.98 – 1.14)	1.47 (1.30 – 1.65)
		Non-migrants	18.6	25	1.02 (0.96 – 1.08)	1.30 (1.21 – 1.40)
	**Other housing material (wood, rammed earth, others)**	Internal migrants	35	26*	1.07 (0.82 – 1.41)	0.96 (0.68 – 1.35)
		Non-migrants	20	25.7	1.07 (0.95 – 1.21)	1.06 (0.88 – 1.29)
	**Age between 18-59**	Internal migrants	18.7	25.4	1.10 (1.00 – 1.28)	1.28 (1.00 – 1.74)
		Non-migrants	19.8	25.3	1.05 (0.94 – 1.17)	1.21 (1.06 – 1.37)
	**Age 60 or older**	Internal migrants	18.9	25.4	1.04 (1.00 – 1.11)	1.37 (1.23 – 1.54)
		Non-migrants	18.9	25.1	1.06 (0.99 – 1.13)	1.29 (1.20 – 1.40)
	**Moving from less populated area to less populated area (Less than 200,000 to Less than 200,00)**	Internal migrants	19.8	25.5	1.04 (0.91 – 1.18)	1.49 (1.26 – 1.76)
	**Moving from less populated area to more populated area (Less than 500,000 to More than 500,00)**	Internal migrants	19.3	24.7	1.17 (1.04 – 1.31)	1.30 (1.04 – 1.62)
	**Biome of death place: Amazon Forest**	Internal migrants	33.4	27.6*	0.93 (0.72 – 1.21)	0.87 (0.64 – 1.19)
		Non-migrants	11.7	27.9*	1.15 (0.92 – 1.43)	0.83 (0.68 – 1.01)
	**Biome of death place: Caatinga**	Internal migrants	26.5	26.7	1.53 (0.63 – 3.69)	1.67 (0.98 – 2.88)
		Non-migrants	14.8	26.6*	0.93 (0.78 – 1.11)	1.28 (1.12 – 1.46)
	**Biome of death place: Cerrado**	Internal migrants	5.9	25.8*	0.92 (0.75 – 1.13)	1.54 (1.24 – 1.93)
		Non-migrants	16	25.6*	0.93 (0.81 – 1.08)	1.38 (1.16 – 1.63)
	**Biome of death place: Atlantic Forest**	Internal migrants	19.3	23.5	1.14 (1.04 – 1.26)	1.34 (1.22 – 1.47)
		Non-migrants	20.8	23.4	1.09 (1.01 – 1.17)	1.19 (1.12 – 1.26)
	**Moving from Northeast region to Northeast region**	Internal migrants	5	26.9*	0.90 (0.84 – 0.97)	1.00 (0.70 – 1.43)
	**Moving from Southeast region to Southeast region**	Internal migrants	20.1	23.2	1.12 (0.95 – 1.31)	1.14 (0.91 – 1.42)
	**Moving from Northeast region to Southeast region**	Internal migrants	19.9	23.9	1.11 (1.01 – 1.22)	1.39 (1.16 – 1.68)
**Renal failure mortality**	**All**	Internal migrants	2.7	25.4*	0.94 (0.70 – 1.27)	1.13 (0.85 – 1.51)
		Non-migrants	23.4	25.2	1.27 (1.05 – 1.55)	1.23 (1.04 – 1.45)
	**Literate**	Internal migrants	11.3	25.3*	0.93 (0.62 – 1.40)	1.01 (0.67 – 1.51)
		Non-migrants	23.7	25	1.14 (0.89 – 1.46)	1.30 (1.05 – 1.62)
	**Illiterate**	Internal migrants	3.2	25.6*	0.78 (0.39 – 1.56)	1.08 (0.56 – 2.08)
		Non-migrants	35.7	25.6*	1.00 (0.55 – 1.79)	0.88 (0.60 – 1.28)
	**Urban-based**	Internal migrants	2.7	25.3*	0.88 (0.63 – 1.23)	1.03 (0.74 – 1.42)
		Non-migrants	23.1	25	1.26 (1.01 – 1.58)	1.29 (1.06 – 1.57)
	**Brick or masonry housing material**	Internal migrants	2.9	25.2*	0.75 (0.53 – 1.06)	0.99 (0.69 – 1.41)
		Non-migrants	23.9	25	1.31 (1.03 – 1.66)	1.24 (1.03 – 1.51)
	**Other housing material (wood, rammed earth, others)**	Internal migrants	25.8	26	2.81 (0.94 – 8.39)	1.57 (0.64 – 3.85)
		Non-migrants	24.9	25.7	1.48 (0.89 – 2.48)	1.18 (0.73 – 1.92)
	**Age between 18-59**	Internal migrants	2.8	25.4*	0.79 (0.40 – 1.55)	1.00 (0.50 – 1.99)
		Non-migrants	23.5	25.3	1.26 (1.04 – 1.53)	1.23 (1.04 – 1.44)
	**Age 60 or older**	Internal migrants	19.2	25.4	1.09 (0.79 – 1.49)	1.32 (0.91 – 1.92)
		Non-migrants	24.2	25.1	1.08 (0.82 – 1.42)	1.35 (1.08 – 1.70)
	**Moving from less populated area to less populated area (Less than 200,000 to Less than 200,00)**	Internal migrants	2.7	25.5*	0.95 (0.55 – 1.63)	0.93 (0.58 – 1.50)
	**Moving from less populated area to more populated area (Less than 500,000 to More than 500,00)**	Internal migrants	3.2	24.7*	0.73 (0.40 – 1.30)	0.92 (0.46 – 1.85)
	**Biome of death place: Amazon Forest**	Internal migrants	27.2	27.6	1.04 (0.59 – 1.84)	1.29 (0.61 – 2.70)
		Non-migrants	33.4	27.9*	0.87 (0.51 – 1.50)	0.95 (0.55 – 1.64)
	**Biome of death place: Caatinga**	Internal migrants	16	26.7*	0.31 (0.03 – 3.70)	2.02 (0.39 – 10.46)
		Non-migrants	14.8	26.6*	0.74 (0.48 – 1.16)	0.95 (0.65 – 1.40)
	**Biome of death place: Cerrado**	Internal migrants	35.7	25.8*	1.18 (0.57 – 2.42)	0.78 (0.41 – 1.48)
		Non-migrants	21.3	25.6	1.15 (0.78 – 1.70)	1.41 (0.90 – 2.19)
	**Biome of death place: Atlantic Forest**	Internal migrants	2.7	23.5*	0.89 (0.61 – 1.31)	1.23 (0.90 – 1.67)
		Non-migrants	23.8	23.4	1.50 (1.14 – 1.98)	1.17 (0.99 – 1.39)
	**Moving from Northeast region to Northeast region**	Internal migrants	5	26.9*	1.10 (0.82 – 1.47)	0.75 (0.25 – 2.24)
	**Moving from Southeast region to Southeast region**	Internal migrants	6.9	23.2*	0.62 (0.31 – 1.21)	1.09 (0.53 – 2.26)
	**Moving from Northeast region to Southeast region**	Internal migrants	5.6	23.9*	0.93 (0.59 – 1.46)	0.97 (0.54 – 1.73)

Note: * indicates median temperatures, which were used instead of the minimum mortality temperature (MMT) to estimate the association in cases where the MMT was not plausible; these values did not fall between the extreme heat and cold thresholds.

Supplementary Table 2. Relative risks and 95% confidence intervals of all-cause and cause-specific mortality associated with extreme ambient temperatures, cumulated over 20 days, across different migration subgroups.

**Cause of death**	Model/ Population (Lag: 0-20 days)	Migration Status	MMT	Median	$RR_{Cold\ (1^{st}\ percentile)}$ (95% CI)	$RR_{Heat\ (99^{th}\ percentile)}$ (95% CI)
**All-cause, non-accidental mortality**	**All**	Internal migrants	25.5	25.4	1.27 (1.22 – 1.32)	1.05 (1.01 – 1.10)
		International migrants	25.1		1.96 (1.10 – 3.50)	1.61 (0.57 – 4.56)
		Non-migrants	27.1	25.2	1.24 (1.20 – 1.28)	1.01 (0.99 – 1.04)
	**Literate**	Internal migrants	24.3	25.3	1.24 (1.17 – 1.31)	1.01 (0.95 – 1.08)
		Non-migrants	35.7	25*	1.20 (1.16 – 1.25)	0.97 (0.94 – 1.01)
	**Illiterate**	Internal migrants	27.9	25.6	1.29 (1.20 – 1.40)	1.01 (0.94 – 1.08)
		Non-migrants	35.7	25.6*	1.12 (1.04 – 1.21)	0.98 (0.93 – 1.03)
	**Urban-based**	Internal migrants	25.7	25.3	1.27 (1.22 – 1.33)	1.06 (1.01 – 1.11)
		Non-migrants	27.2	25	1.25 (1.20 – 1.29)	1.01 (0.99 – 1.04)
	**Brick or masonry housing material**	Internal migrants	25.2	25.2	1.24 (1.19 – 1.30)	1.06 (1.01 – 1.12)
		Non-migrants	26.6	25	1.21 (1.16 – 1.25)	1.03 (1.00 – 1.06)
	**Other housing material (wood, rammed earth, others)**	Internal migrants	25.8	26	1.28 (1.12 – 1.46)	0.96 (0.84 – 1.10)
		Non-migrants	35.7	25.7*	1.31 (1.22 – 1.41)	0.85 (0.80 – 0.91)
	**Age between 18-59**	Internal migrants	35.7	25.4*	1.23 (1.13 – 1.35)	0.97 (0.87 – 1.09)
		Non-migrants	35.7	25.3*	1.23 (1.16 – 1.30)	0.98 (0.93 – 1.02)
	**Age 60 or older**	Internal migrants	26.1	25.4	1.28 (1.22 – 1.33)	1.06 (1.01 – 1.11)
		Non-migrants	28.5	25.1	1.22 (1.17 – 1.27)	1.00 (1.00 – 1.02)
	**Moving from less populated area to less populated area (Less than 200,000 to Less than 200,00)**	Internal migrants	24.9	25.5	1.18 (1.10 – 1.27)	1.02 (0.96 – 1.10)
	**Moving from less populated area to more populated area (Less than 500,000 to More than 500,00)**	Internal migrants	35.7	24.7*	1.28 (1.19 – 1.37)	0.92 (0.83 – 1.03)
	**Biome of death place: Amazon Forest**	Internal migrants	33.4	27.6*	1.04 (0.91 – 1.19)	0.79 (0.71 – 0.88)
		Non-migrants	31	27.9*	1.37 (1.21 – 1.56)	0.86 (0.79 – 0.92)
	**Biome of death place: Caatinga**	Internal migrants	16	26.7*	0.96 (0.73 – 1.28)	1.11 (0.92 – 1.34)
		Non-migrants	28.7	26.6	1.17 (1.10 – 1.24)	1.01 (0.97 – 1.05)
	**Biome of death place: Cerrado**	Internal migrants	25.3	25.8	1.17 (1.06 – 1.29)	1.19 (1.08 – 1.32)
		Non-migrants	27.1	25.6	1.07 (1.00 – 1.15)	1.05 (0.99 – 1.12)
	**Biome of death place: Atlantic Forest**	Internal migrants	25	23.5	1.32 (1.25 – 1.40)	1.08 (1.03 – 1.13)
		Non-migrants	25.1	23.4	1.22 (1.17 – 1.28)	1.05 (1.03 – 1.08)
	**Moving from Northeast region to Northeast region**	Internal migrants	5	26.9*	1.11 (1.00 – 1.23)	1.00 (0.93 – 1.08)
	**Moving from Southeast region to Southeast region**	Internal migrants	24.5	23.2	1.33 (1.21 – 1.45)	1.08 (0.97 – 1.19)
	**Moving from Northeast region to Southeast region**	Internal migrants	25.1	23.9	1.23 (1.16 – 1.30)	1.12 (1.03 – 1.23)
**Circulatory system mortality**	**All**	Internal migrants	35.7	25.4*	1.32 (1.20 – 1.45)	0.93 (0.84 – 1.03)
		Non-migrants	27	25.2	1.41(1.31 – 1.51)	1.04 (0.99 – 1.09)
	**Literate**	Internal migrants	35.7	25.3*	1.30 (1.14 – 1.48)	0.99 (0.85 – 1.15)
		Non-migrants	27.1	25	1.41 (1.29 – 1.55)	1.02 (0.96 – 1.10)
	**Illiterate**	Internal migrants	35.7	25.6*	1.41 (1.16 – 1.73)	0.71 (0.56 – 0.89)
		Non-migrants	35.7	25.6*	1.16 (0.96 – 1.41)	0.98 (0.87 – 1.11)
	**Urban-based**	Internal migrants	35.7	25.3*	1.34 (1.21 – 1.49)	0.91 (0.81 – 1.02)
		Non-migrants	26.5	25	1.39 (1.28 – 1.51)	1.06 (0.99 – 1.14)
	**Brick or masonry housing material**	Internal migrants	35.7	25.2*	1.30 (1.18 – 1.45)	0.94 (0.83 – 1.07)
		Non-migrants	26.5	25	1.37 (1.26 – 1.50)	1.06 (1.00 – 1.14)
	**Other housing material (wood, rammed earth, others)**	Internal migrants	25.8	26	1.35 (0.96 – 1.89)	0.83 (0.58 – 1.17)
		Non-migrants	35.7	25.7*	1.62 (1.35 – 1.94)	0.92 (0.78 – 1.07)
	**Age between 18-59**	Internal migrants	35.7	25.4*	1.38 (1.13 – 1.67)	0.82 (0.62 – 1.09)
		Non-migrants	26.2	25.3	1.36 (1.18 – 1.57)	1.05 (0.94 – 1.18)
	**Age 60 or older**	Internal migrants	35.7	25.4*	1.31 (1.17 – 1.48)	0.93 (0.82 – 1.06)
		Non-migrants	28	25.1	1.40 (1.27 – 1.55)	1.01 (0.95 – 1.07)
	**Moving from less populated area to less populated area (Less than 200,000 to Less than 200,00)**	Internal migrants	35	25.5*	1.24 (1.03 – 1.50)	0.86 (0.72 – 1.03)
	**Moving from less populated area to more populated area (Less than 500,000 to More than 500,00)**	Internal migrants	35.7	24.7*	1.34 (1.15 – 1.56)	0.92 (0.72 – 1.18)
	**Biome of death place: Amazon Forest**	Internal migrants	33.4	27.6*	1.01 (0.73 – 1.40)	0.89 (0.69 – 1.15)
		Non-migrants	29	27.9	1.29 (0.95 – 1.76)	1.07 (0.90 – 1.27)
	**Biome of death place: Caatinga**	Internal migrants	16	26.7*	1.18 (0.57 – 2.46)	0.99 (0.61 – 1.63)
		Non-migrants	34.6	26.6*	1.20 (1.05 – 1.37)	0.93 (0.83 – 1.05)
	**Biome of death place: Cerrado**	Internal migrants	24.7	25.8	1.31 (1.04 – 1.66)	1.12 (0.9 – 1.42)
		Non-migrants	28.8	25.6	1.30 (1.10 – 1.54)	1.01 (0.91 – 1.12)
	**Biome of death place: Atlantic Forest**	Internal migrants	34.8	23.5*	1.39 (1.22 – 1.58)	0.88 (0.79 – 0.98)
		Non-migrants	25.3	23.4	1.34 (1.21 – 1.48)	1.07 (1.02 – 1.14)
	**Moving from Northeast region to Northeast region**	Internal migrants	34.3	26.9*	1.05 (0.97 – 1.13)	0.90 (0.60 – 1.33)
	**Moving from Southeast region to Southeast region**	Internal migrants	34.6	23.2*	1.45 (1.17 – 1.78)	0.95 (0.75 – 1.21)
	**Moving from Northeast region to Southeast region**	Internal migrants	34.8	23.9*	1.24 (1.10 – 1.41)	0.90 (0.73 – 1.10)
**Respiratory disease mortality**	**All**	Internal migrants	22.5	25.4	1.30 (1.11 – 1.52)	1.32 (1.10 – 1.58)
		Non-migrants	26.1	25.2	1.28 (1.15 – 1.43)	1.06 (1.00 – 1.17)
	**Literate**	Internal migrants	21.9	25.3	1.38 (1.10 – 1.72)	1.09 (0.84 – 1.42)
		Non-migrants	35.7	25*	1.18 (1.02 – 1.36)	0.98 (0.85 – 1.13)
	**Illiterate**	Internal migrants	21.2	25.6	1.11 (0.79 – 1.55)	1.84 (1.25 – 2.71)
		Non-migrants	26.2	25.6	1.40 (1.06 – 1.85)	1.10 (0.89 – 1.36)
	**Urban-based**	Internal migrants	23	25.3	1.29 (1.08 – 1.54)	1.40 (1.14 – 1.72)

		Non-migrants	26.6	25	1.26 (1.11 – 1.42)	1.04 (0.93 – 1.17)
	**Brick or masonry housing material**	Internal migrants	22.8	25.2	1.22 (1.02 – 1.45)	1.40 (1.12 – 1.74)
		Non-migrants	25.5	25	1.21 (1.06 – 1.38)	1.10 (0.98 – 1.24)
	**Other housing material (wood, rammed earth, others)**	Internal migrants	25.8	26	1.41 (0.82 – 2.41)	0.82 (0.42 – 1.63)
		Non-migrants	35.7	25.7*	1.23 (0.94 – 1.61)	0.73 (0.53 – 0.99)
	**Age between 18-59**	Internal migrants	35.7	25.4*	1.08 (0.75 – 1.55)	0.91 (0.52 – 1.59)
		Non-migrants	27.3	25.3	1.23 (1.00 – 1.56)	1.04 (0.86 – 1.26)
	**Age 60 or older**	Internal migrants	23	25.4	1.25 (1.04 – 1.51)	1.35 (1.09 – 1.67)
		Non-migrants	23.1	25.1	1.22 (1.06 – 1.40)	1.02 (0.90 – 1.17)
	**Moving from less populated area to less populated area (Less than 200,000 to Less than 200,00)**	Internal migrants	21.1	25.5	1.17 (0.86 – 1.60)	1.28 (0.93 – 1.76)
	**Moving from less populated area to more populated area (Less than 500,000 to More than 500,00)**	Internal migrants	35.7	24.7*	1.38 (1.07 – 1.76)	0.86 (0.57 – 1.30)
	**Biome of death place: Amazon Forest**	Internal migrants	10.5	27.6*	0.82 (0.46 – 1.45)	0.76 (0.46 – 1.25)
		Non-migrants	11.7	27.9*	1.62 (1.03 – 2.55)	0.75 (0.54 – 1.03)
	**Biome of death place: Caatinga**	Internal migrants	27.1	26.7	1.26 (0.29 – 5.48)	2.32 (0.87 – 6.20)
		Non-migrants	28	26.6	1.23 (0.94 – 1.62)	1.15 (0.94 – 1.41)
	**Biome of death place: Cerrado**	Internal migrants	5.9	25.8*	0.96 (0.66 – 1.41)	1.53 (1.01 – 2.31)
		Non-migrants	6.5	25.6*	1.19 (0.92 – 1.55)	1.19 (0.89 – 1.60)
	**Biome of death place: Atlantic Forest**	Internal migrants	23.4	23.5	1.37 (1.11 – 1.69)	1.42 (1.19 – 1.71)
		Non-migrants	24.7	23.4	1.34 (1.15 – 1.56)	1.11 (1.00 – 1.23)
	**Moving from Northeast region to Northeast region**	Internal migrants	5	26.9*	1.04 (0.96 – 1.13)	1.04 (0.54 – 2.00)
	**Moving from Southeast region to Southeast region**	Internal migrants	23.5	23.2	1.27 (0.90 – 1.78)	1.40 (0.92 – 2.13)
	**Moving from Northeast region to Southeast region**	Internal migrants	23.2	23.9	1.28 (1.04 – 1.58)	1.37 (0.97 – 1.95)
**Renal failure mortality**	**All**	Internal migrants	20.7	25.4	1.28 (0.72 – 2.26)	1.41 (0.83 – 2.39)
		Non-migrants	23	25.2	1.13 (0.78 – 1.64)	1.20 (0.90 – 1.60)
	**Literate**	Internal migrants	14.6	25.3	1.00 (0.76 – 1.32)	1.98 (0.78 – 5.00)
		Non-migrants	17.7	25	1.01 (0.69 – 1.47)	1.58 (1.05 – 2.38)
	**Illiterate**	Internal migrants	22.7	25.6	4.76 (1.07 – 21.09)	1.68 (0.47 – 5.97)
		Non-migrants	35.7	25.6*	1.01 (0.34 – 2.95)	0.70 (0.37 – 1.32)
	**Urban-based**	Internal migrants	20.4	25.3	1.22 (0.63 – 2.35)	1.55 (0.84 – 2.82)
		Non-migrants	22.3	25	1.09 (0.72 – 1.67)	1.32 (0.93 – 1.87)
	**Brick or masonry housing material**	Internal migrants	2.9	25.2*	0.66 (0.34 – 1.29)	1.17 (0.61 – 2.24)
		Non-migrants	22.9	25	1.26 (0.80 – 1.97)	1.29 (0.92 – 1.81)
	**Other housing material (wood, rammed earth, others)**	Internal migrants	25.8	26	2.49 (0.27 – 22.74)	1.08 (0.18 – 6.62)
		Non-migrants	0.8	25.7*	1.04 (0.40 – 2.6 5)	1.10 (0.50 – 2.40)
	**Age between 18-59**	Internal migrants	2.8	25.4*	0.48 (0.13 – 1.84)	1.09 (0.31 – 3.84)
		Non-migrants	23.2	25.3	1.13 (0.79 – 1.61)	1.20 (0.90 – 1.60)
	**Age 60 or older**	Internal migrants	20.7	25.4	1.70 (0.81 – 3.56)	1.57 (0.80 – 3.07)
		Non-migrants	2.4	25.1*	0.85 (0.50 – 1.44)	1.32 (0.88 – 1.98)
	**Moving from less populated area to less populated area (Less than 200,000 to Less than 200,00)**	Internal migrants	35	25.5*	1.20 (0.41 – 3.56)	0.74 (0.30 – 1.85)
	**Moving from less populated area to more populated area (Less than 500,000 to More than 500,00)**	Internal migrants	11.5	24.7*	0.60 (0.20 – 1.82)	1.31 (0.38 – 4.52)
	**Biome of death place: Amazon Forest**	Internal migrants	10.5	27.6*	0.74 (0.18 – 3.04)	1.85 (0.53 – 6.43)
		Non-migrants	33.4	27.9*	0.89 (0.27 – 2.95)	0.72 (0.31 – 1.70)
	**Biome of death place: Caatinga**	Internal migrants	16	26.7*	0.36 (0.00 – 22.95)	0.73 (0.04 – 12.96)
		Non-migrants	14.8	26.6*	0.66 (0.33 – 1.32)	0.82 (0.43 – 1.54)
	**Biome of death place: Cerrado**	Internal migrants	35.7	25.8*	0.67 (0.16 – 2.84)	0.68 (0.22 – 2.09)
		Non-migrants	35.7	25.6*	1.42 (0.61 – 3.29)	1.03 (0.50 – 2.07)
	**Biome of death place: Atlantic Forest**	Internal migrants	21.3	23.5	1.62 (0.71 – 3.70)	1.60 (0.92 – 2.79)
		Non-migrants	24.3	23.4	1.23 (0.72 – 2.09)	1.39 (1.03 – 1.88)
	**Moving from Northeast region to Northeast region**	Internal migrants	34.3	26.9*	1.07 (0.71 – 1.61)	3.48 (0.52 – 23.33)
	**Moving from Southeast region to Southeast region**	Internal migrants	18.6	23.2	1.48 (0.45 – 4.89)	2.38 (0.69 – 8.24)
	**Moving from Northeast region to Southeast region**	Internal migrants	21.2	23.9	1.38 (0.58 – 3.29)	1.43 (0.51 – 3.98)

Note: * indicates median temperatures, which were used instead of the minimum mortality temperature (MMT) to estimate the association in cases where the MMT was not plausible; these values did not fall between the extreme heat and cold thresholds.

Over 0-6 days, we observed the consistent patterns of higher all-cause [RR_{Mig}(95%CI):1.15(1.12–1.17) vs. $RR_{Non\text{-}mig}$:1.12(1.10–1.14); after extreme-heat], [RR_{Mig}(95%CI):1.11(1.09–1.13) vs. $RR_{Non\text{-}mig}$:1.10(1.08–1.12); after extreme-cold] and respiratory disease mortality [RR_{Mig}(95%CI):1.37(1.24–1.52) vs. $RR_{Non\text{-}mig}$:1.29(1.21–1.37); after extreme-heat], [RR_{Mig}(95%CI):1.09(1.01–1.16) vs. $RR_{Non\text{-}mig}$:1.04(0.99–1.10); after extreme-cold], and lower circulatory mortality among internal-migrants compared to non-migrants [RR_{Mig}(95%CI):1.16(1.11–1.22) vs. $RR_{Non\text{-}mig}$:1.21(1.16–1.26); after extreme-cold] (Supplementary Table1-2). Across migration-flow categories over 0-20 days, the stronger association for cold-related all-cause mortality was observed among individuals who migrated from municipalities with fewer than 500,000 to those with ≥500,000 inhabitants (RR 1.28, 1.19–1.37). Heat-related all-cause mortality was not significantly associated with any migration-flow category. Similar patterns were observed for circulatory mortality. For respiratory mortality, cold-related risks also tended to increase with migration to populated

municipalities (with RR 1.38, 1.07–1.76), whereas the association for migration between municipalities with fewer than 200,000 inhabitants was not significant (RR 1.17, 0.86–1.60). No significant associations were observed between extreme heat and respiratory mortality across migration-flow categories over 0-20 days (Supplementary Table 2). Over 0-6 days, migrants who moved to more populated areas had an elevated risk of cardiovascular mortality after extreme-heat (RR:1.21; 95%CI:1.05–1.39), whilst it was not significant in people who moved to less populated area (RR:1.00; 95%CI:0.91–1.10) (Supplementary Table1 and Supplementary Figure7-8).

<table>
<tr><th>Lag</th><th colspan="2">Lag: 0-6 days</th></tr>
<tr><td>Cause of death</td><td>Non-migrants</td><td>Internal migrants</td></tr>
<tr><td>All-cause non-accidental mortality</td><td>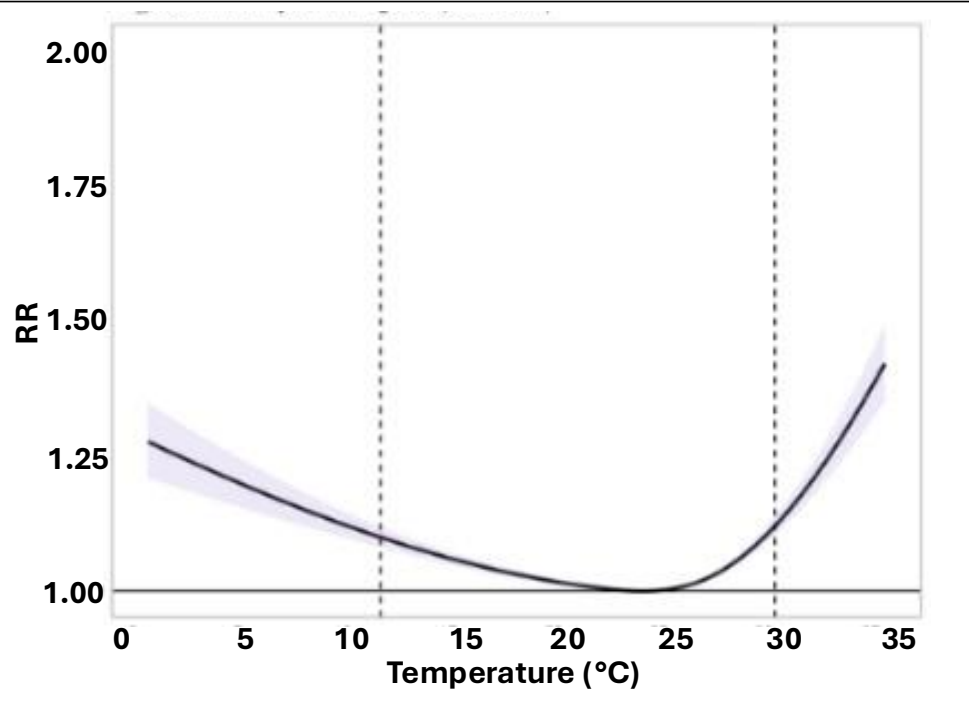
</td><td>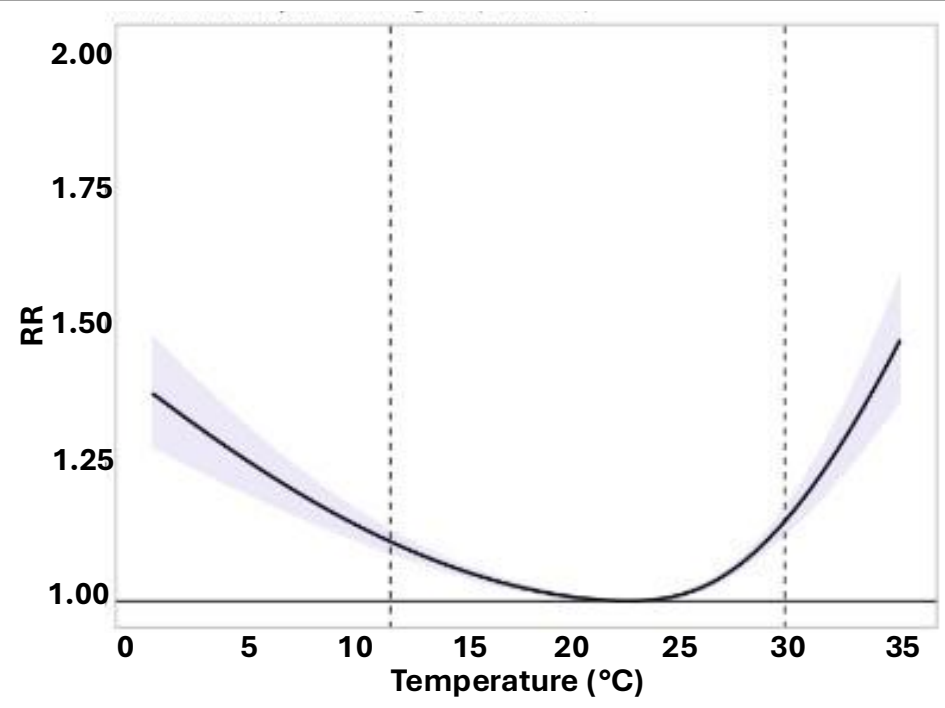
</td></tr>
<tr><td>Circulatory system mortality</td><td>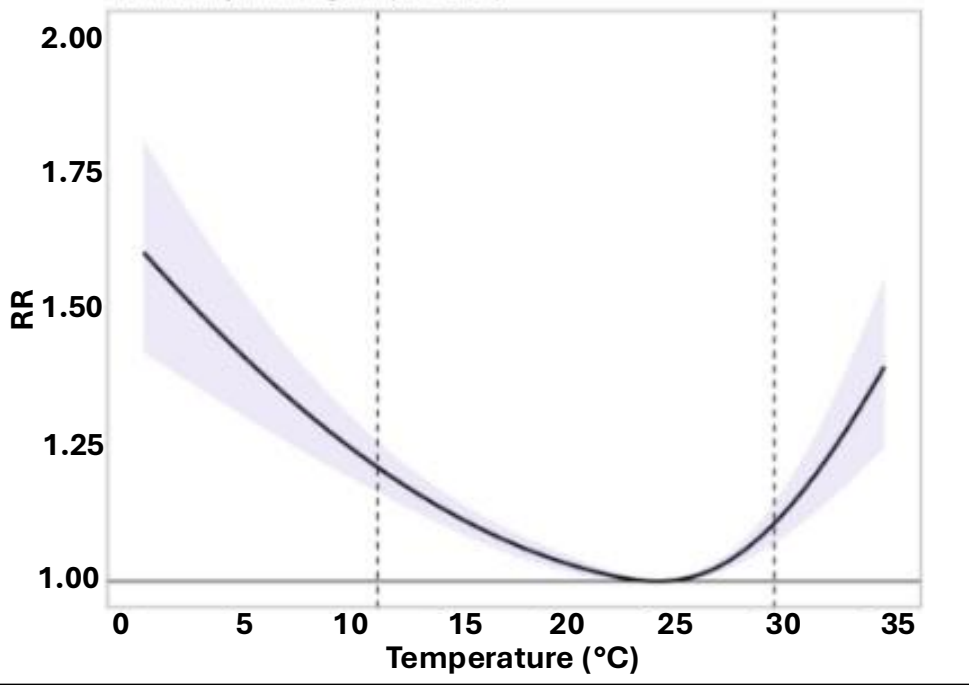
</td><td>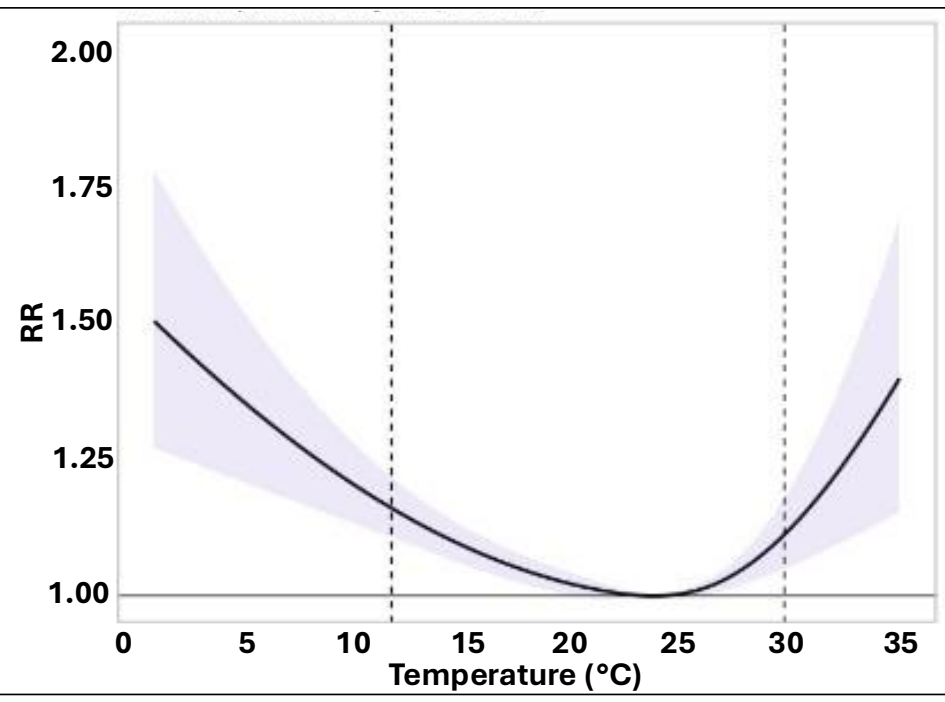
</td></tr>
<tr><td>Respiratory system mortality</td><td>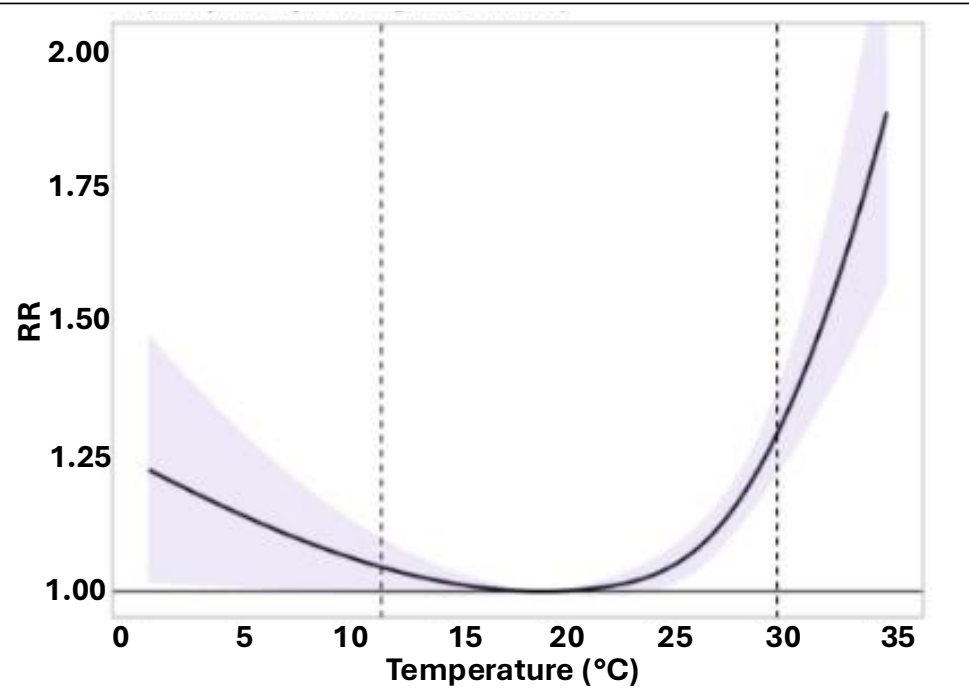
</td><td>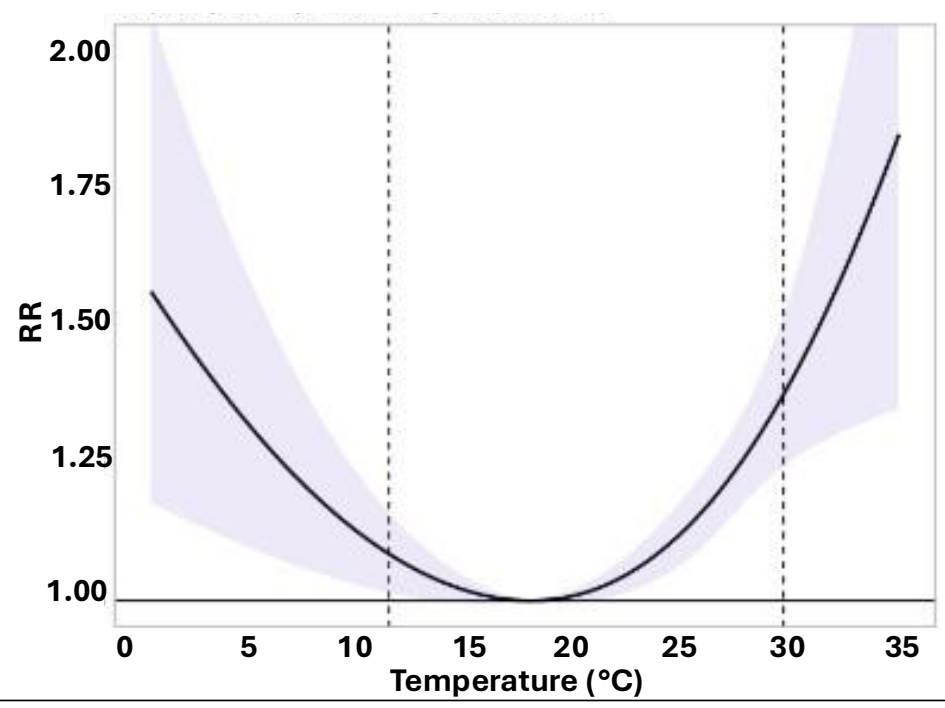
</td></tr>
<tr><td>Renal failure mortality</td><td>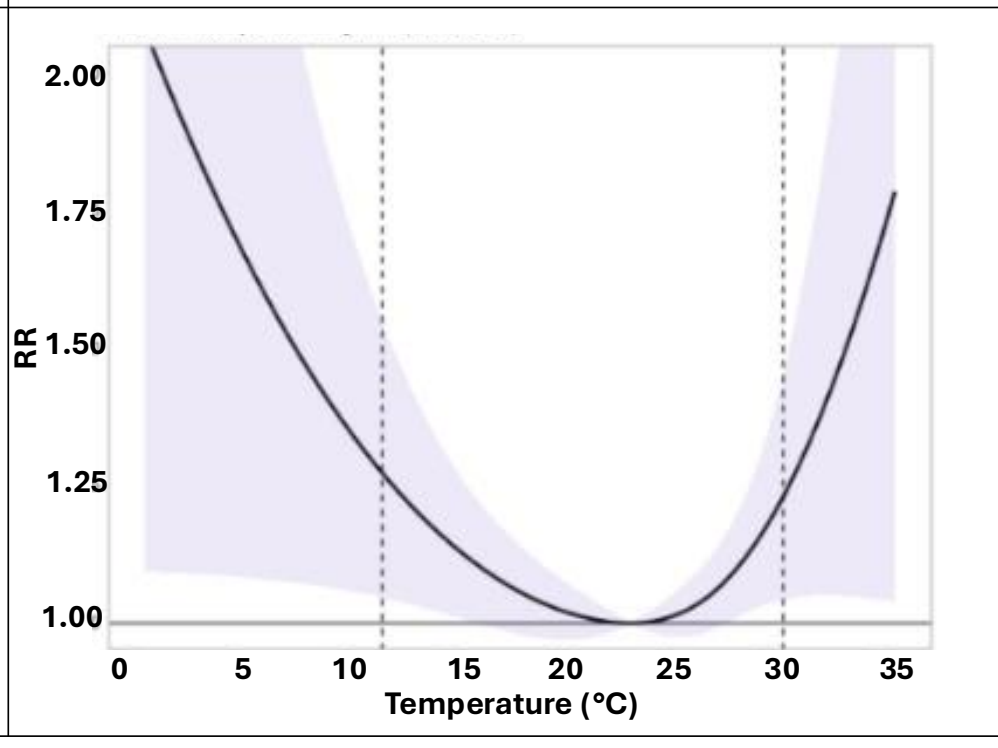
</td><td>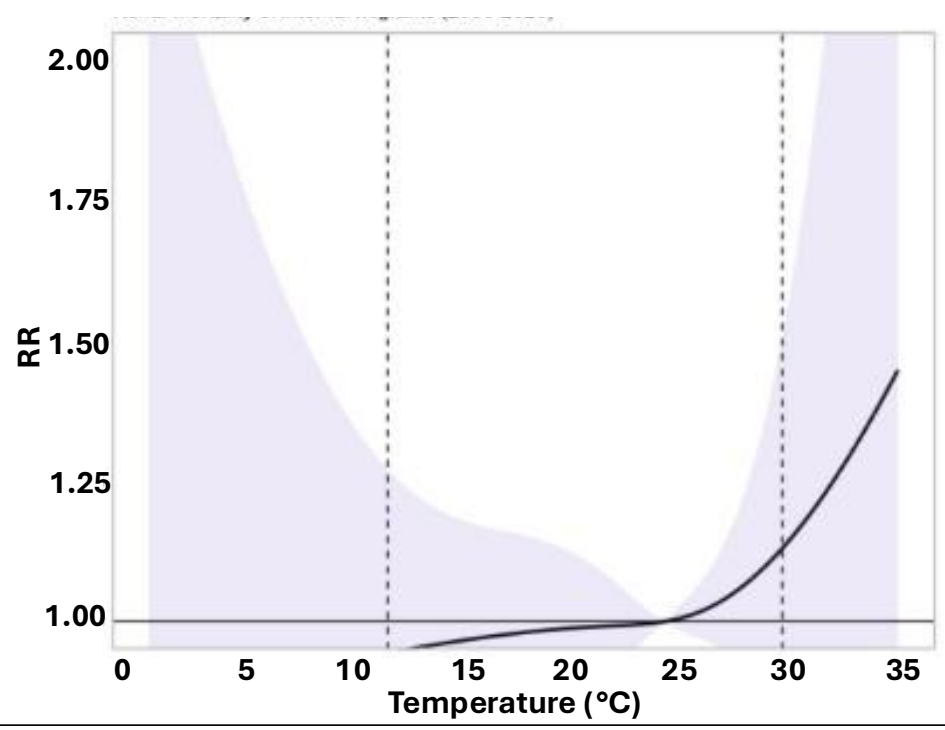
</td></tr>
</table>

Supplementary Figure 4. Exposure–response association between ambient temperature (°C) and all-cause and cause-specific mortality among non-migrants and internal migrants, over 0-6 days, in the 100 Million Brazilian Cohort (2000–2020). The shaded areas represent the 95% confidence intervals, and the dotted lines indicate the 1st (extreme-cold) and 99th (extreme-heat) percentiles of the weighted mean temperature distribution. Temperature (°C) is shown on the x-axis and relative risk (RR) on the y-axis. The solid line represents the estimated relative risk.

Exposure–lag–response surfaces revealed non-linear associations between ambient temperature and mortality in both non-migrants and internal migrants (Supplementary Figure 5).

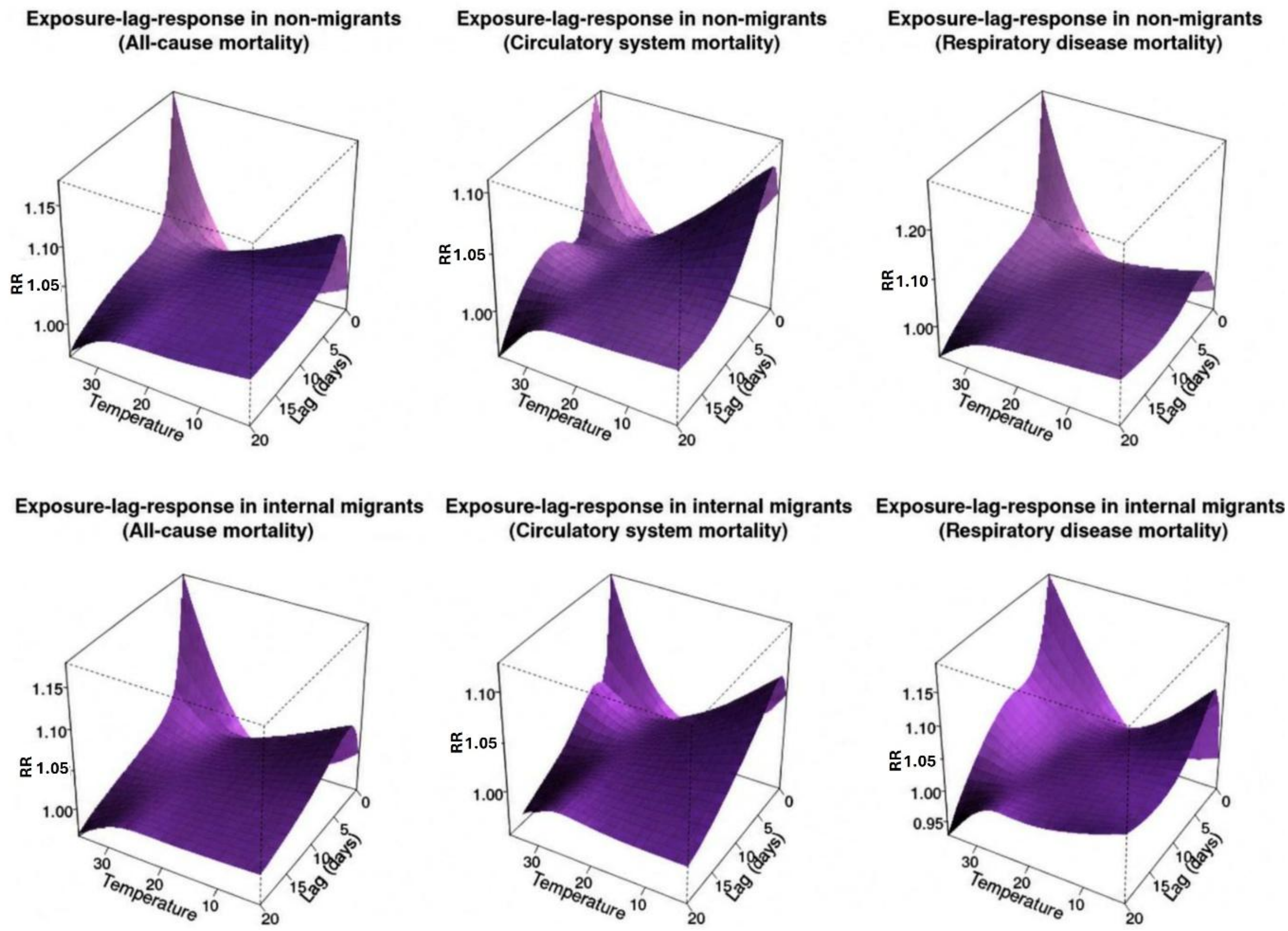

Supplementary Figure 5. Exposure–lag–response curves for all cause and cause specific mortality among non-migrants and internal migrants across lag 0-20 days.

Lag-response curves (Supplementary Figure 6) show the relative risk of mortality associated with exposure to an extreme heat and extreme cold temperature compared with the reference temperature (MMT), for lags 0-20 days. The curves show the estimated relative risk for each lag day following exposure and should not be interpreted as overall cumulative effects. Cumulative relative risks over the full 0-20-day lag period are presented in Supplementary Table 2 which is calculated as the sum of the lag-specific log-RRs across the entire 0-20 lag window. These are not directly comparable in direction, because the cumulative estimate integrates both the acute positive effect at short lags (e.g., 0-3 days) and the smaller reduction at longer lags (e.g., 10-20 days). Because the magnitude of the acute short-lag risk increase exceeds that of the longer-lag risk reduction, the cumulative effect remains positive, as shown in Supplementary Table 2.

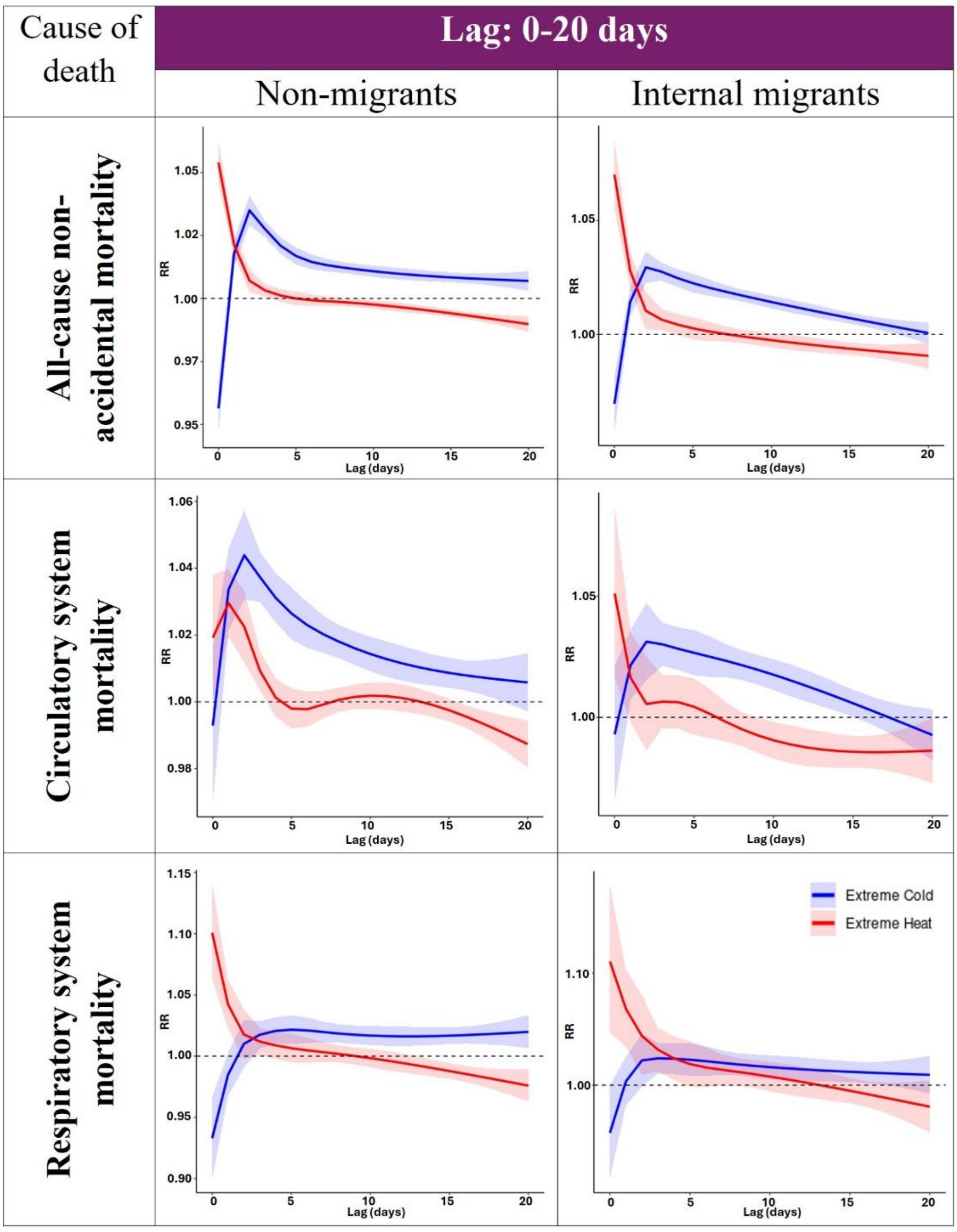

Supplementary Figure 6. Lag-response curves for all-cause and cause-specific mortality among non-migrants and internal migrants across lag 0-20 days.

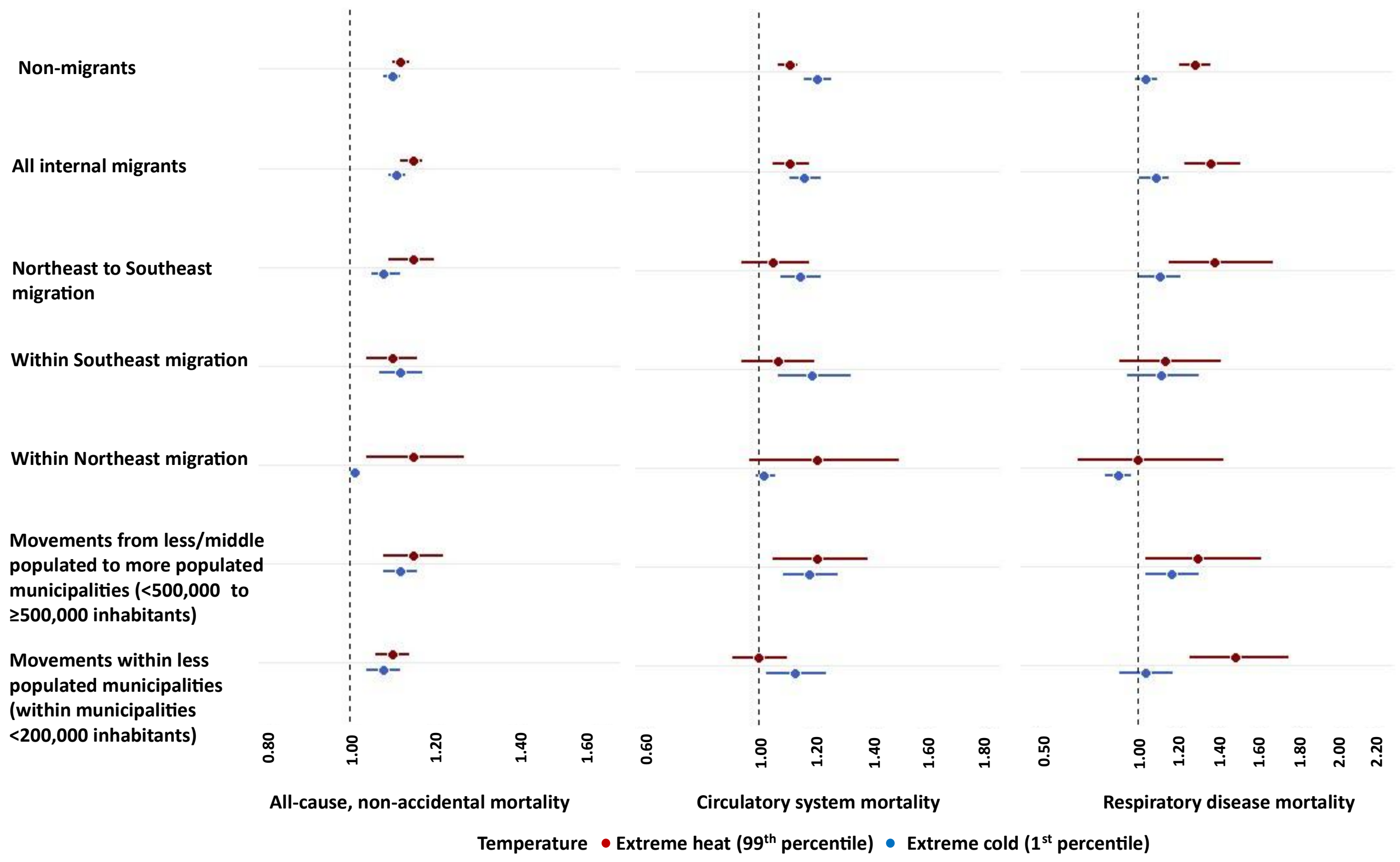

Supplementary Figure 7. Relative risks (RR) and 95% confidence intervals (CI) of all-cause and cause-specific mortality associated with extreme ambient temperatures, cumulated over lag of 0-6 days, for non-migrants, all internal migrants, and subgroups of internal migrants categorized by different migration routes. The horizontal axis shows the cumulative relative risk of mortality over lag days. Red color indicates RR and 95% CI associated with extreme heat, while blue color indicates RR and 95% CI for extreme cold.

Cause of death	Non-migrants	Internal migrants	Moving from less populated area to less populated area (Less than 200,000 to Less than 200,000)	Moving from less populated area to more populated area (Less than 500,000 to More than 500,00)	Internal migrants moved from Northeast region to Southeast region	Moving from Northeast region to Northeast region	Moving from Southeast region to Southeast region
All-cause non-accidental mortality	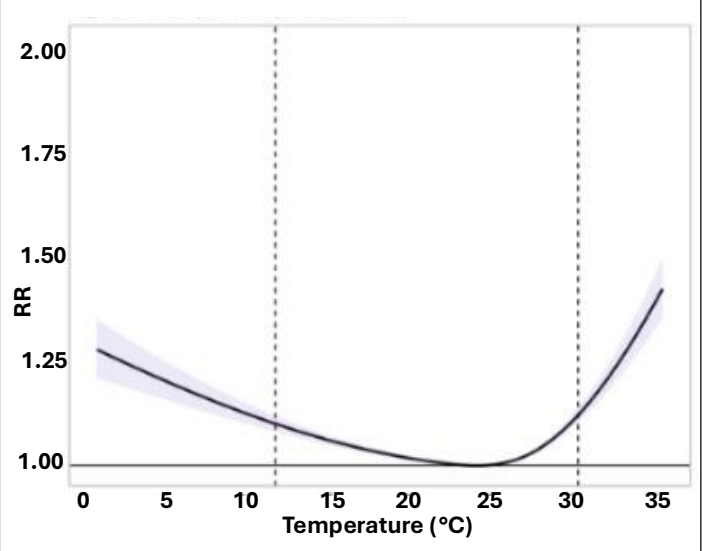	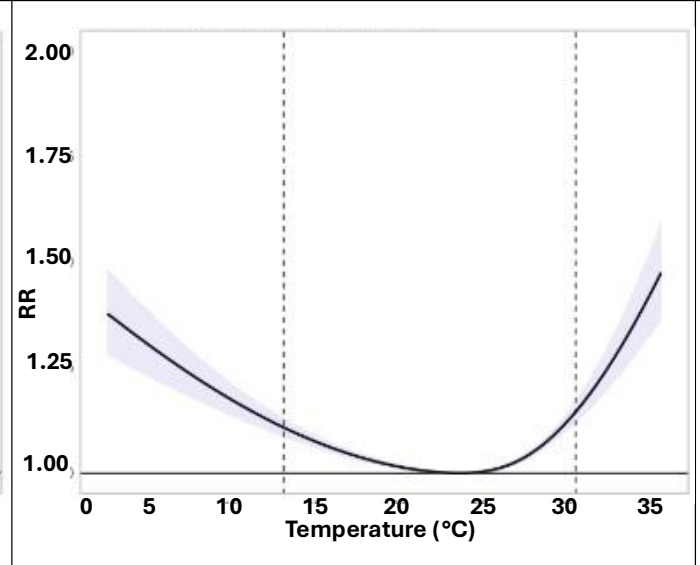	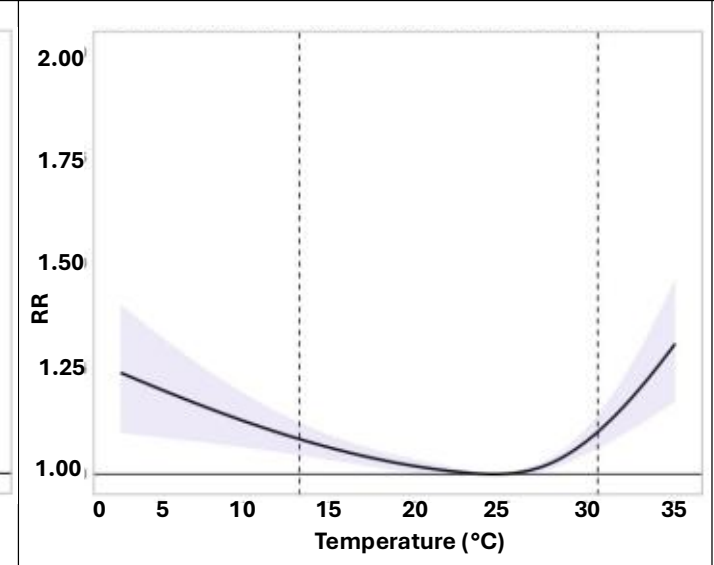	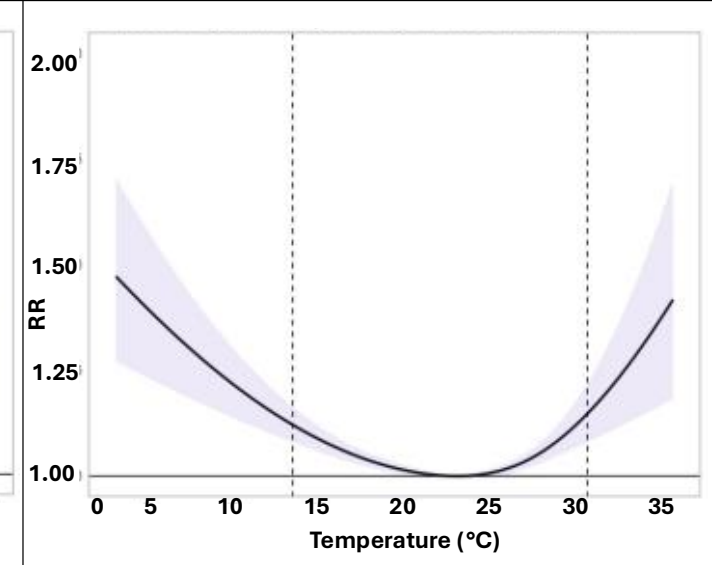	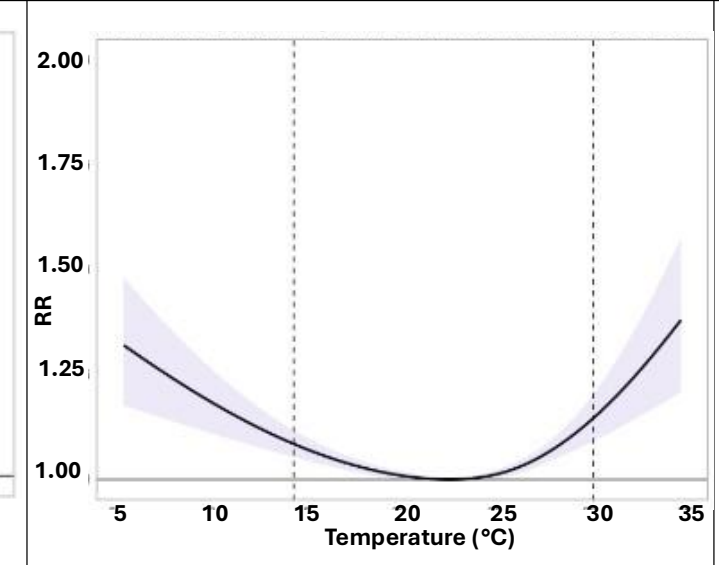	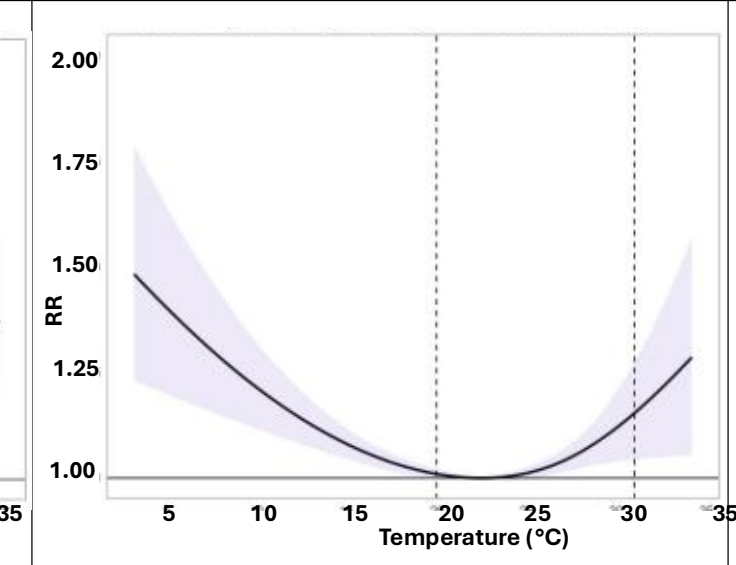	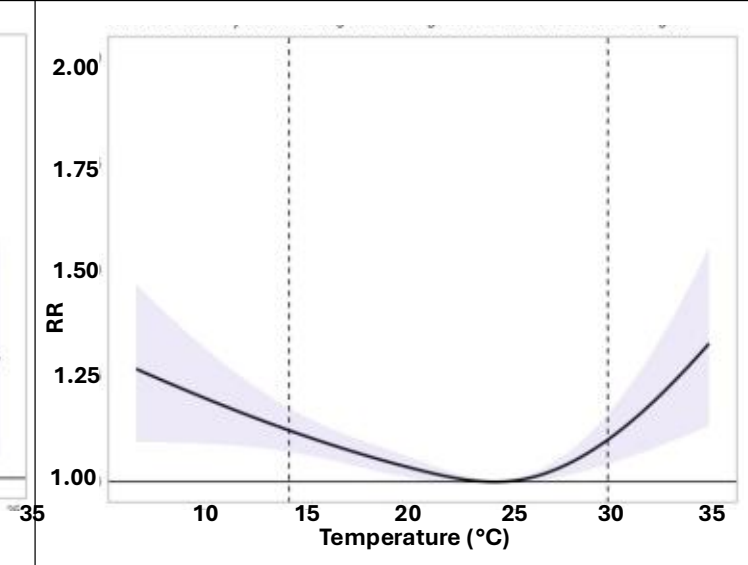
Circulatory system mortality	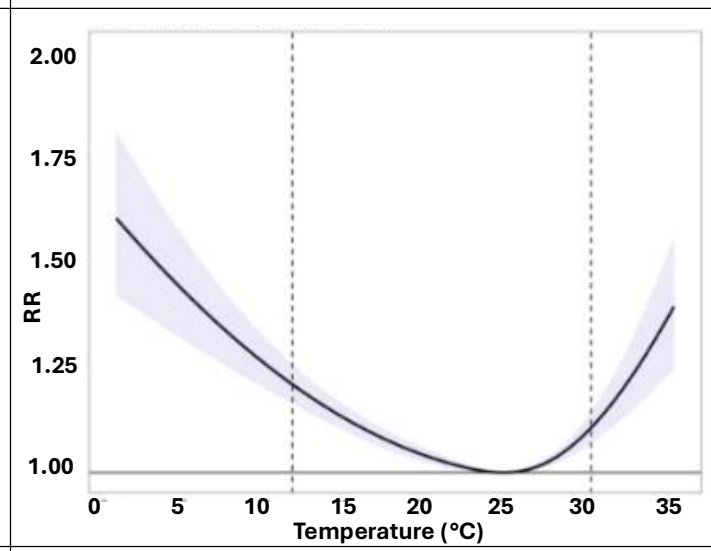	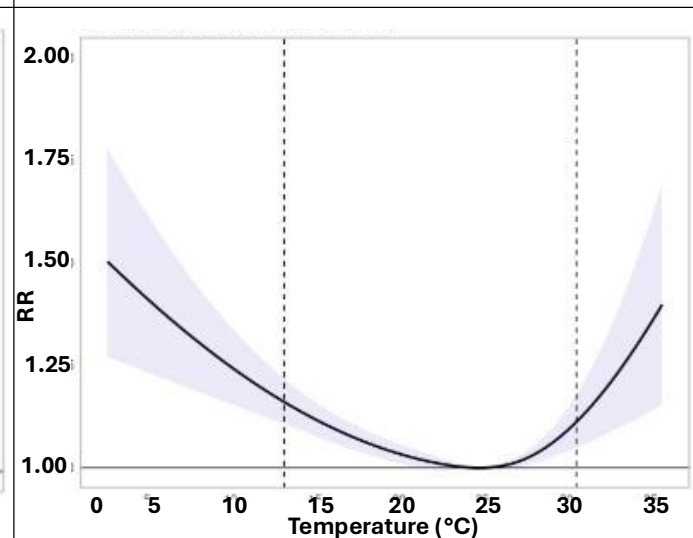	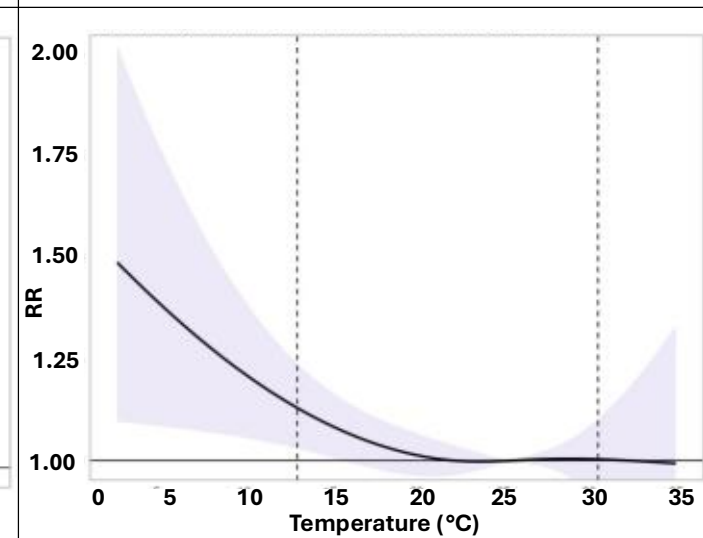	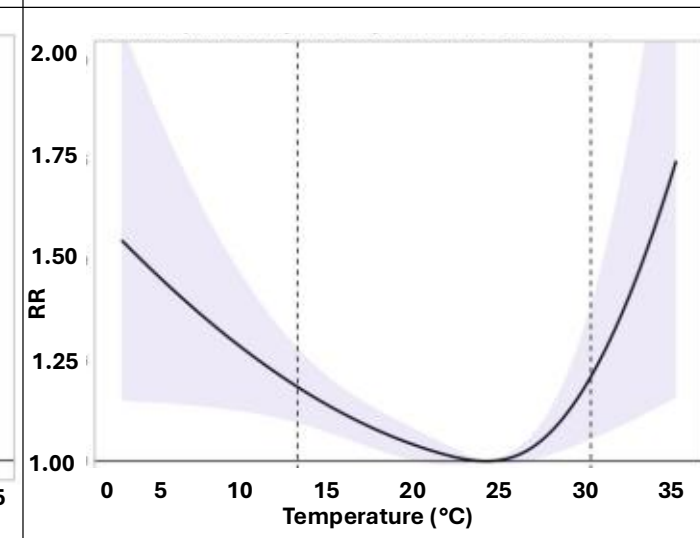	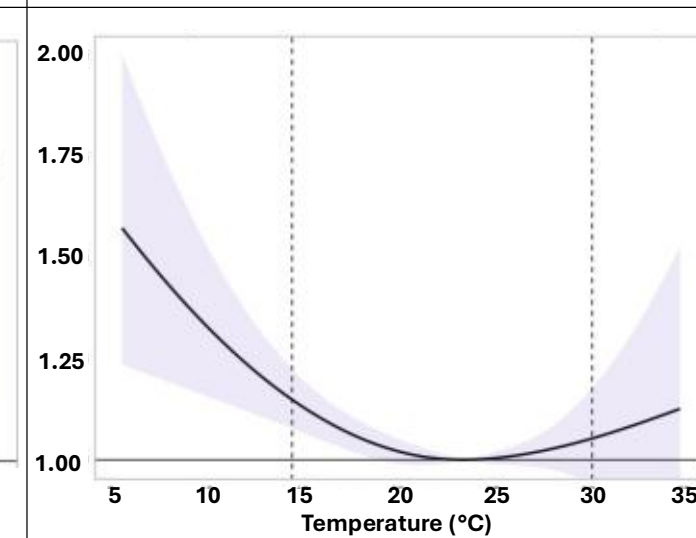	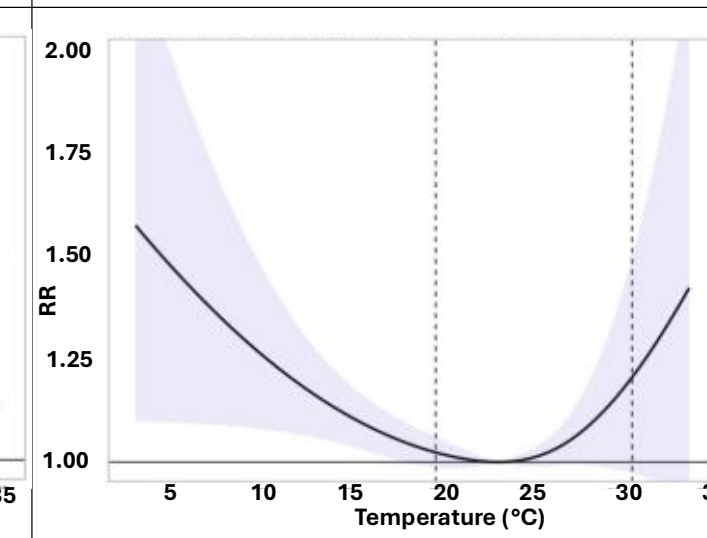	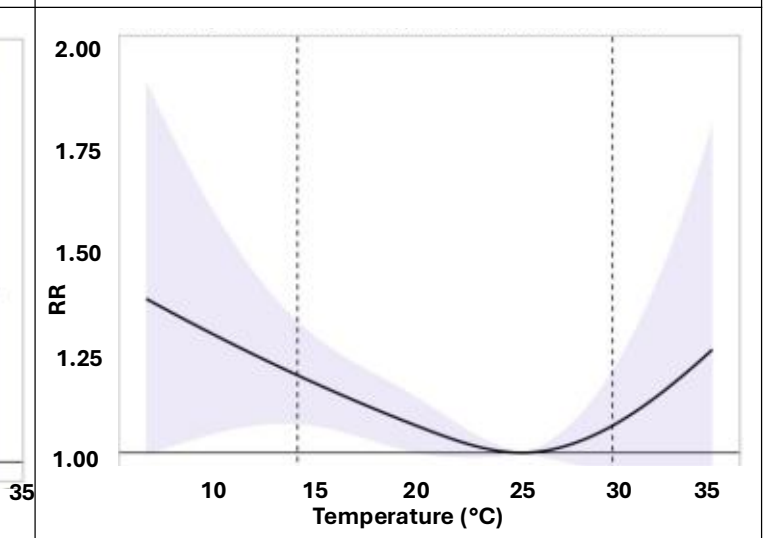
Respiratory system mortality	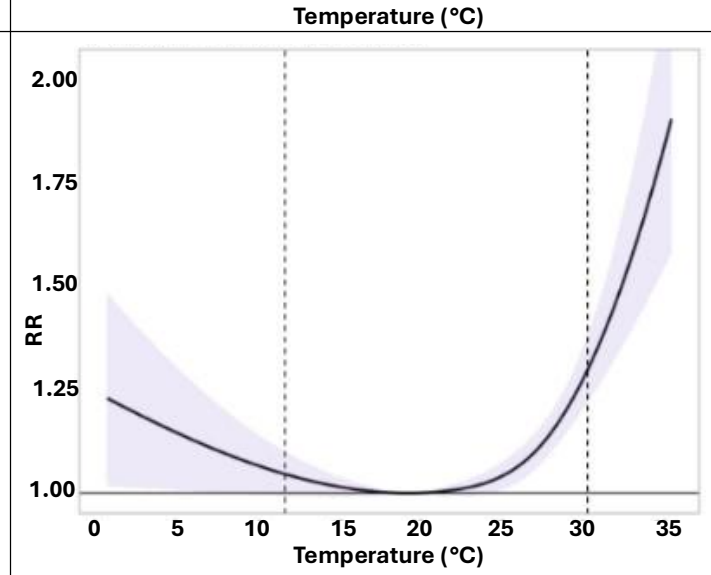	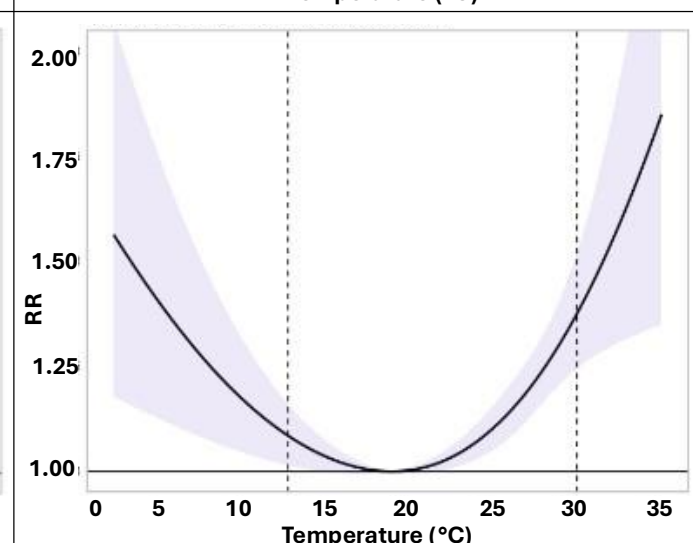	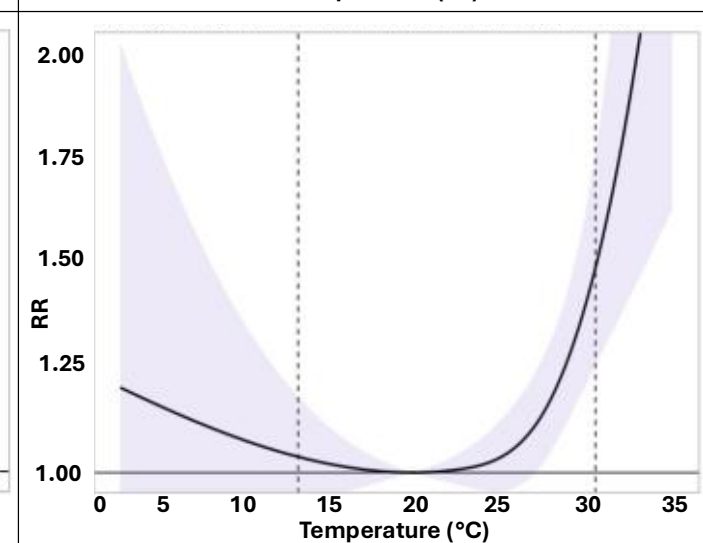	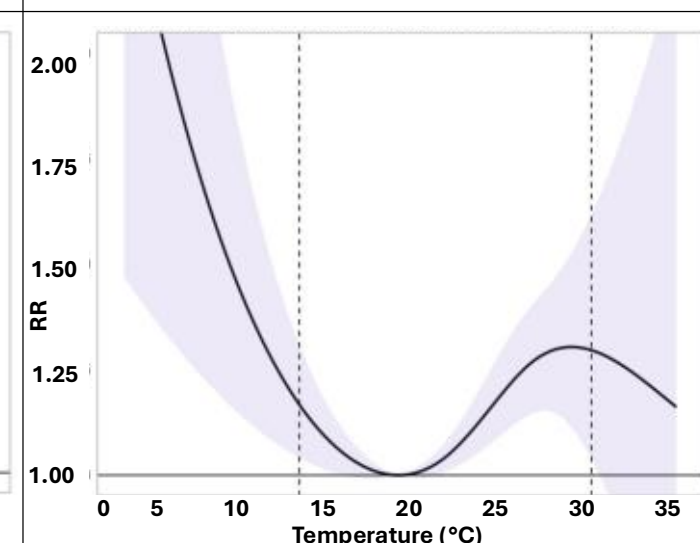	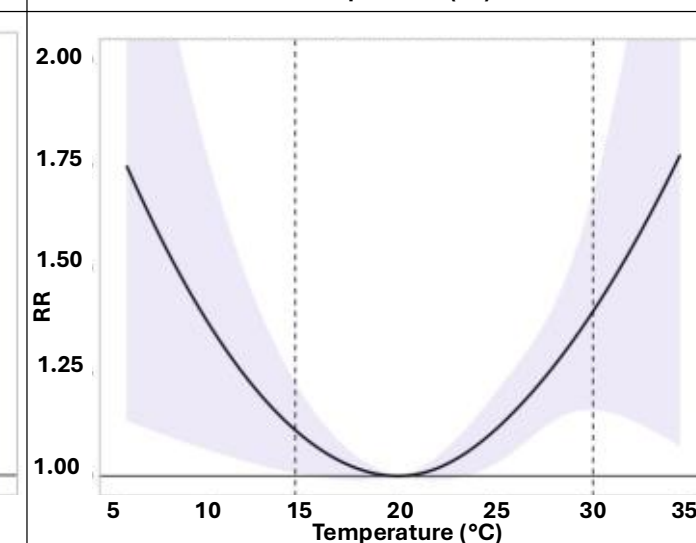	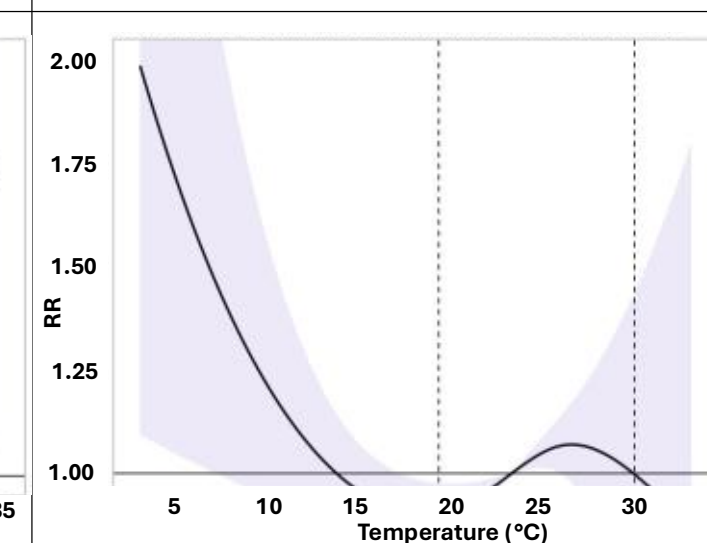	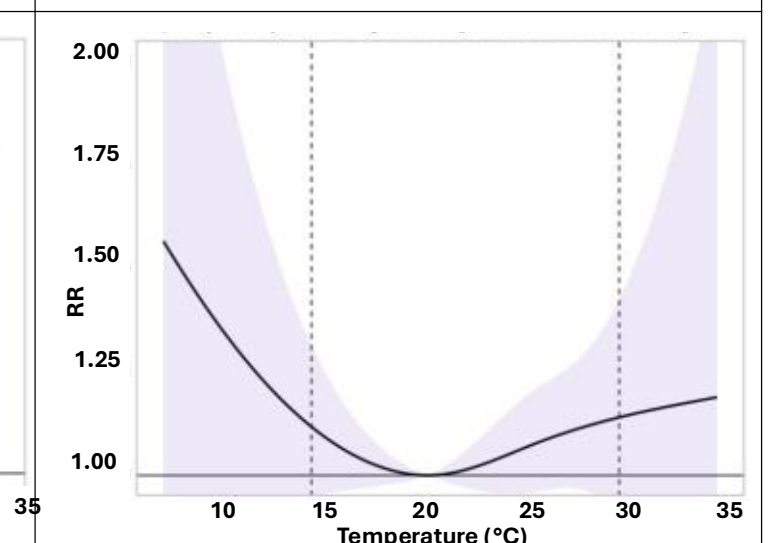
Renal failure mortality	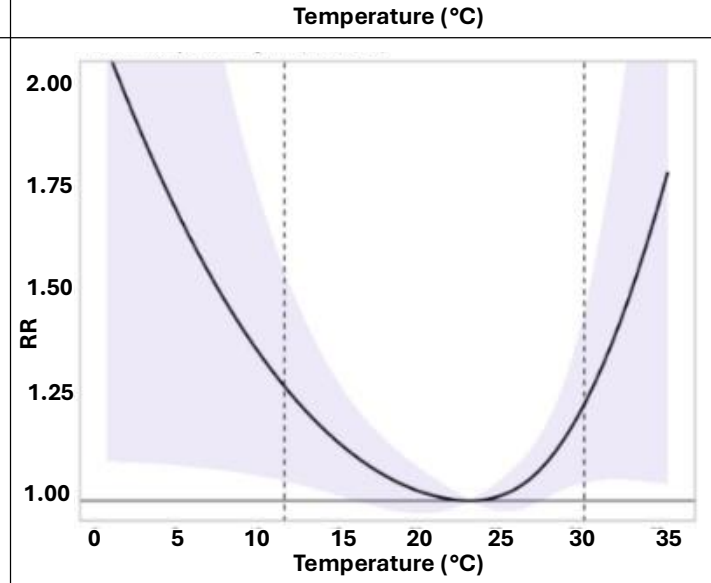	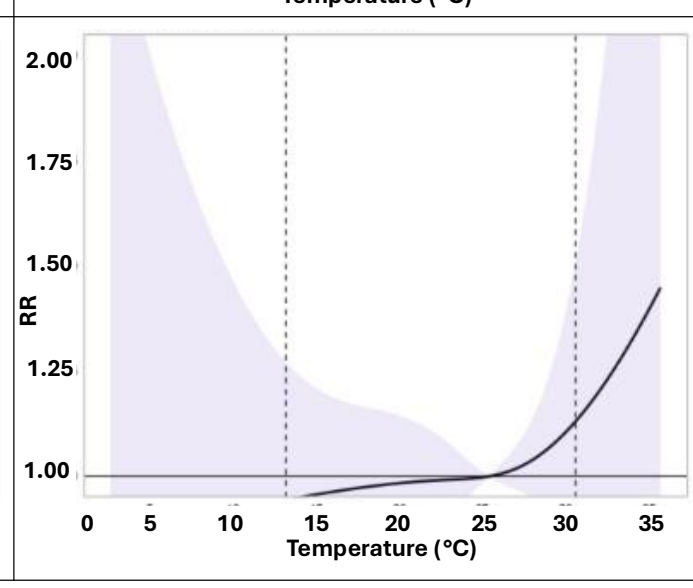	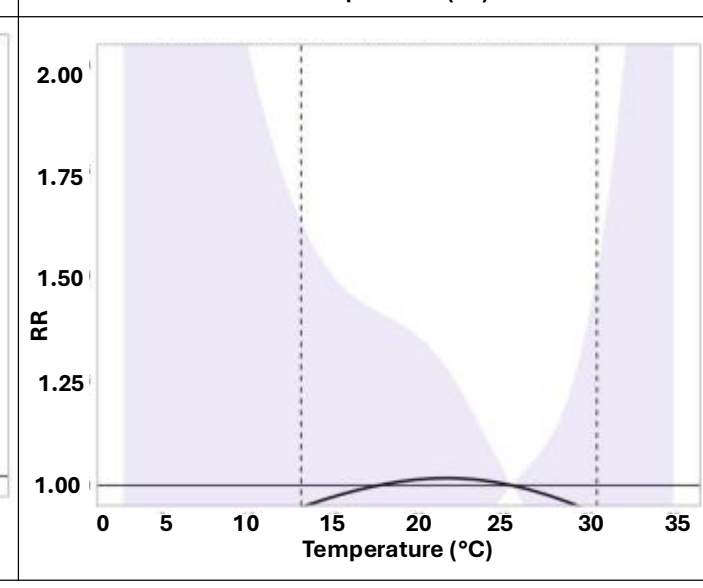	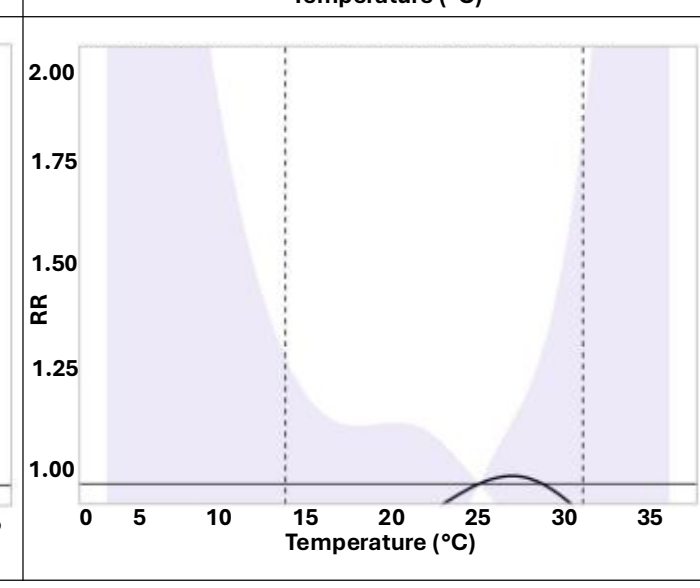	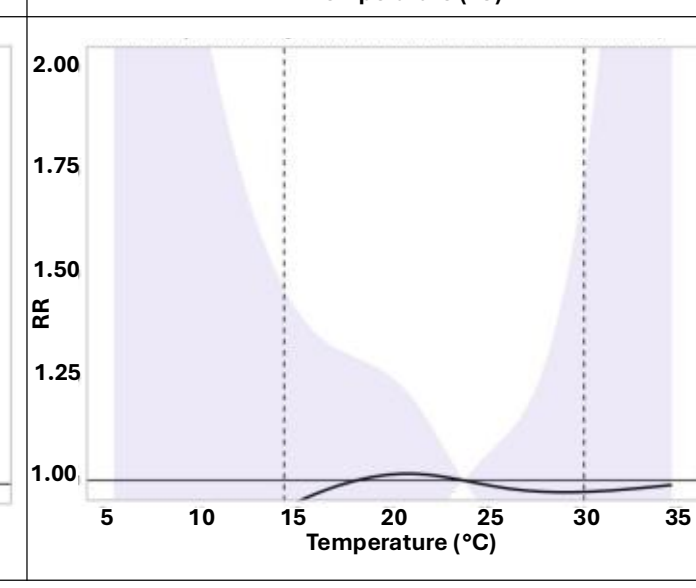	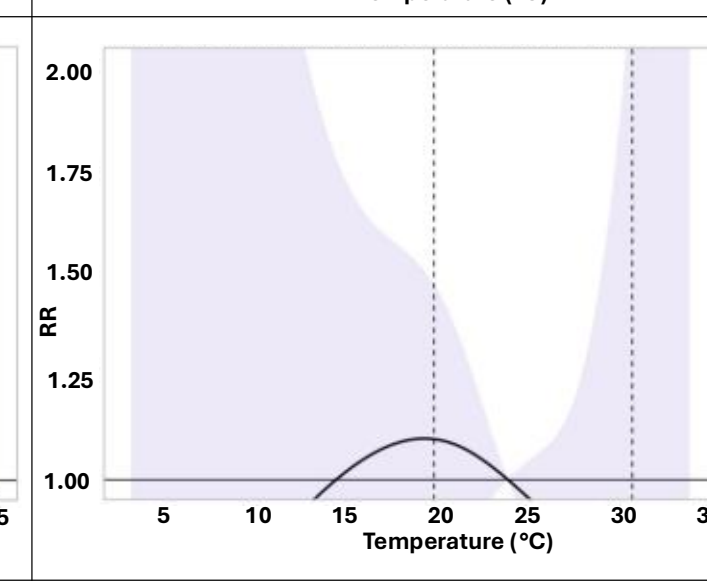	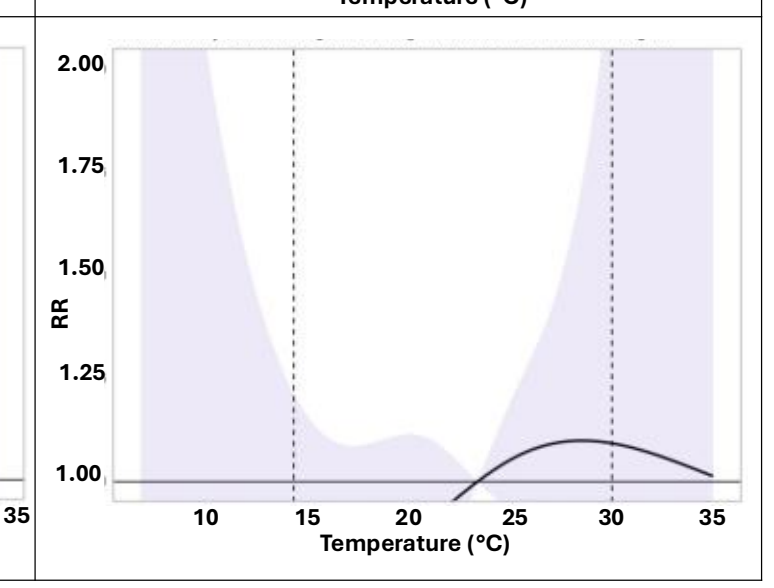

Supplementary Figure 8. Relative risks of **all-cause and cause-specific mortality** associated with extreme ambient temperature cumulated over **6-day**, among non-migrants and different sub-categories of migrants. Dotted vertical lines show the 0.01 and 0.99 temperature percentiles.

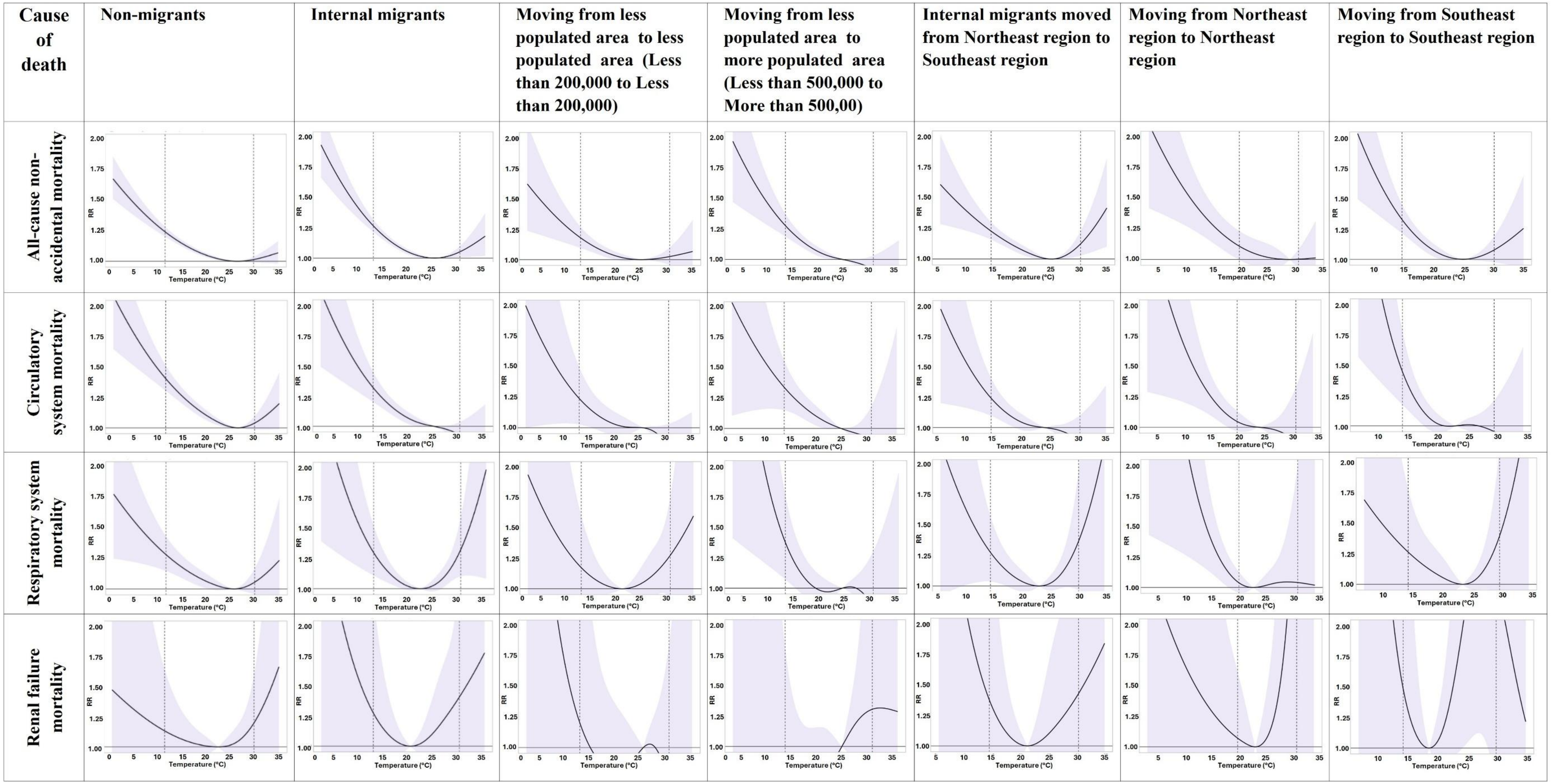

Supplementary Figure 9. Relative risks of **all-cause and cause-specific mortality** associated with extreme ambient temperature cumulated over **20-day**, among non-migrants and different sub-categories of migrants. Dotted vertical lines show the 0.01 and 0.99 temperature percentiles.

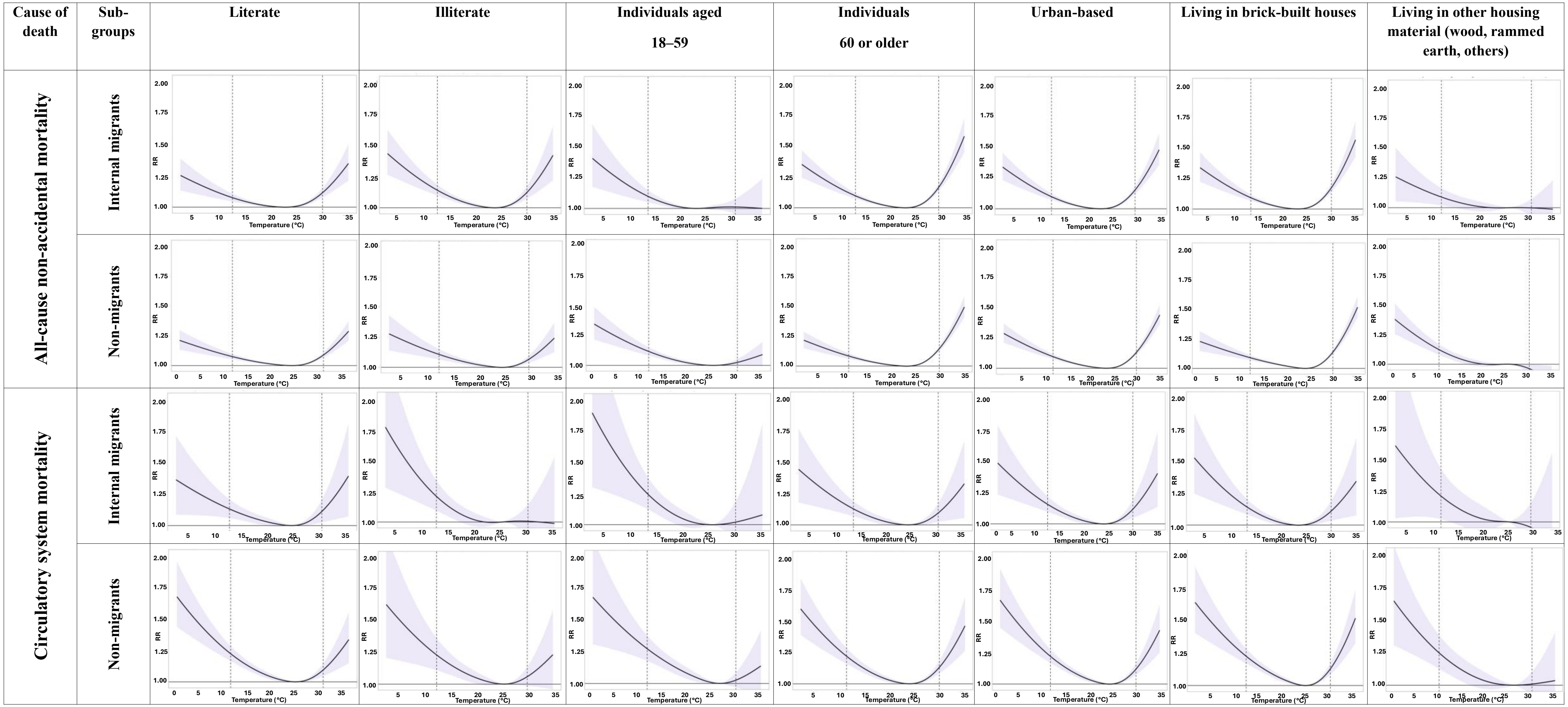

Supplementary Figure 10. Relative risks of **all-cause non-accidental mortality and circulatory system mortality** associated with extreme ambient temperature cumulated over 0-6-day lag periods for different individual characteristics and housing conditions. Dotted vertical lines show the 0.01 and 0.99 temperature percentiles.

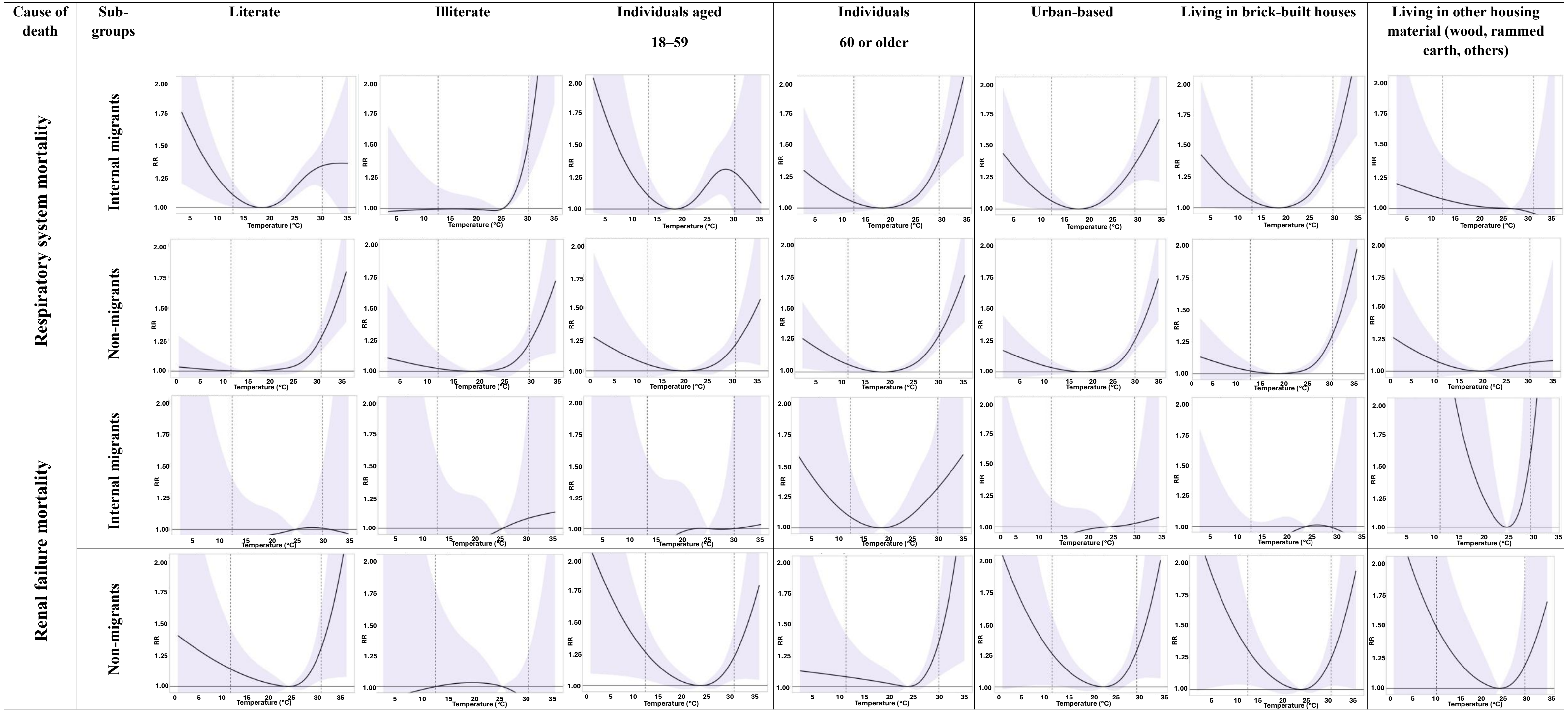

Supplementary Figure 11. Relative risks of **respiratory system mortality** and **renal failure mortality** associated with extreme ambient temperature cumulated over 0-6-day lag periods for different individual characteristics and housing conditions. Dotted vertical lines show the 0.01 and 0.99 temperature percentiles.

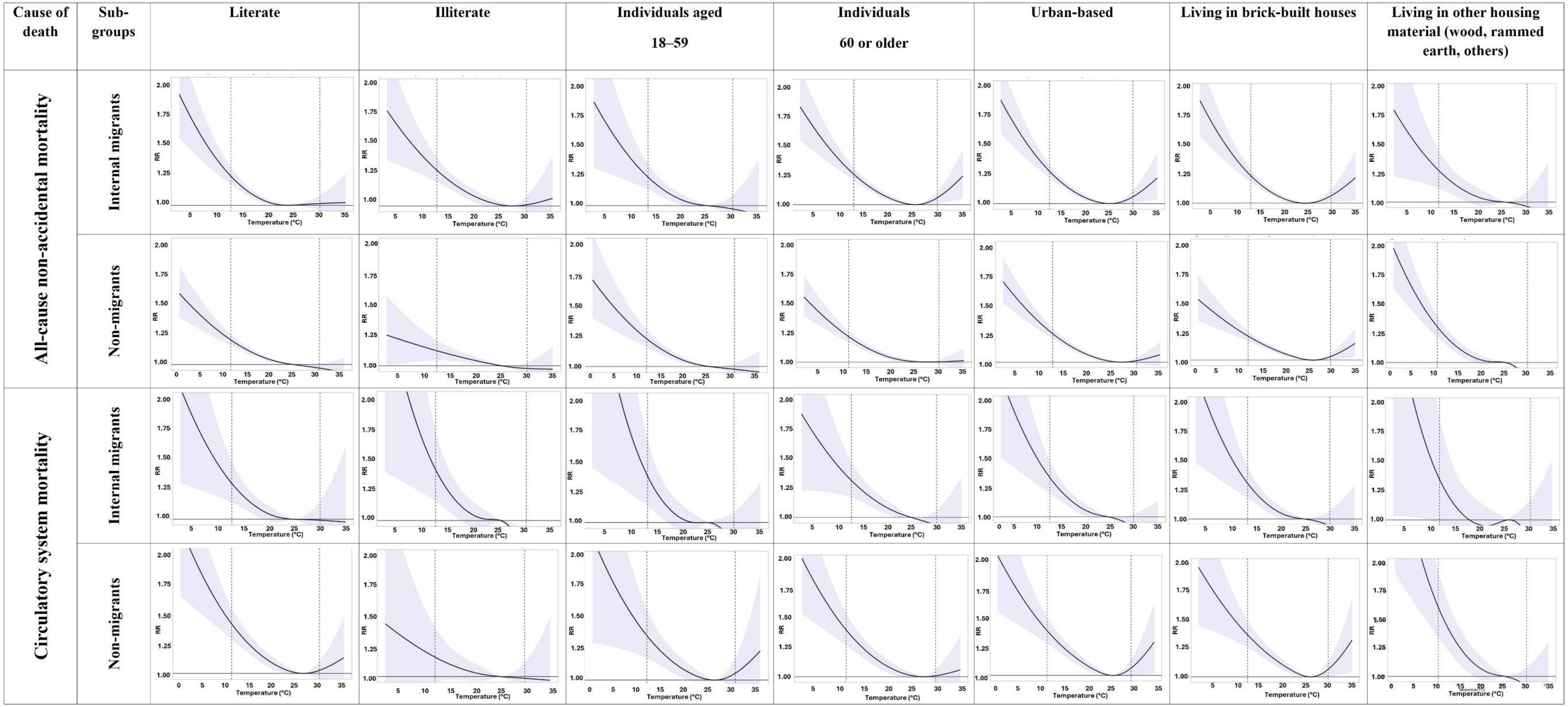

Supplementary Figure 12. Relative risks of **all-cause non-accidental mortality and circulatory system mortality** associated with extreme ambient temperature cumulated over 0-20-day lag periods for different individual characteristics and housing conditions. Dotted vertical lines show the 0.01 and 0.99 temperature percentiles.

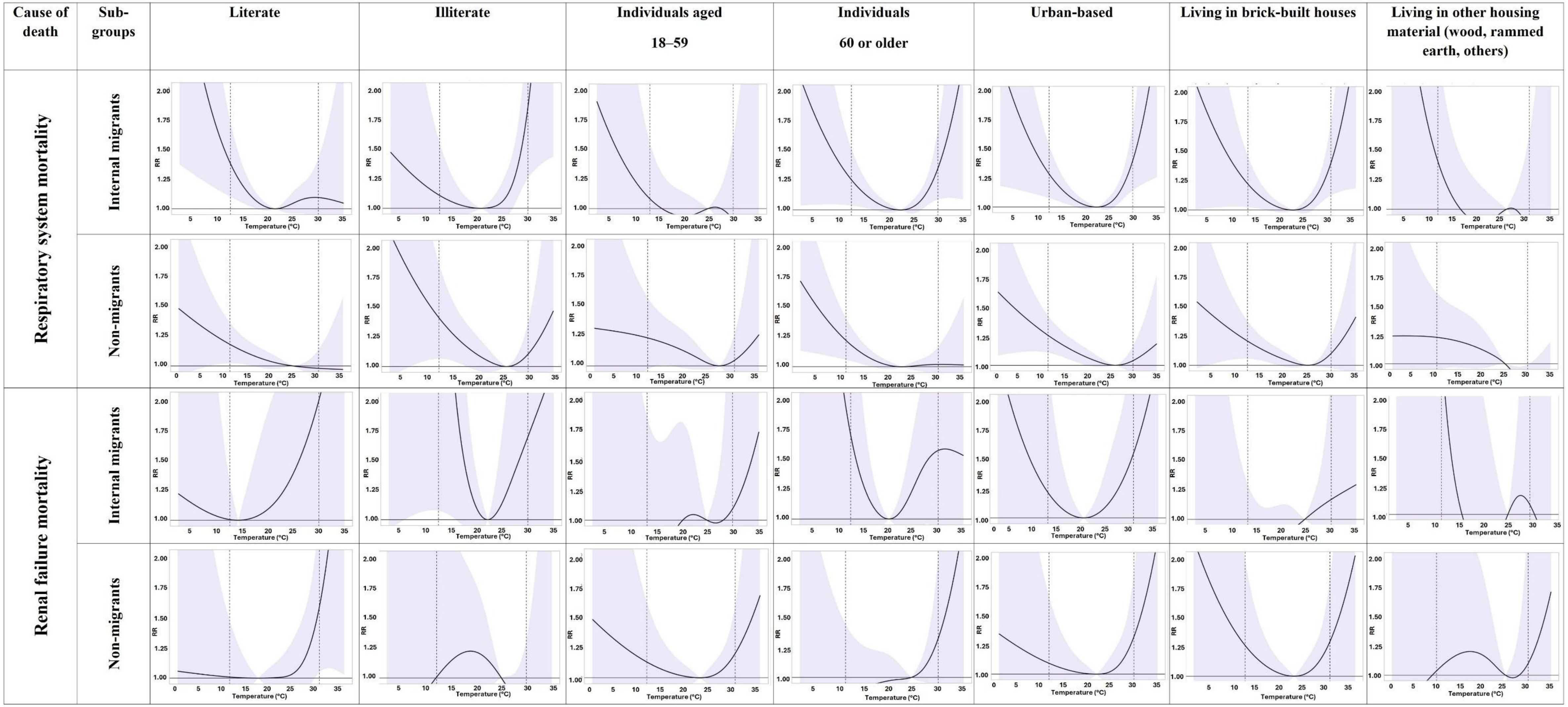

Supplementary Figure 13. Relative risks of **respiratory system mortality** and **renal failure mortality** associated with extreme ambient temperature cumulated over 0-20-day lag periods for different individual characteristics and housing conditions. Dotted vertical lines show the 0.01 and 0.99 temperature percentiles.

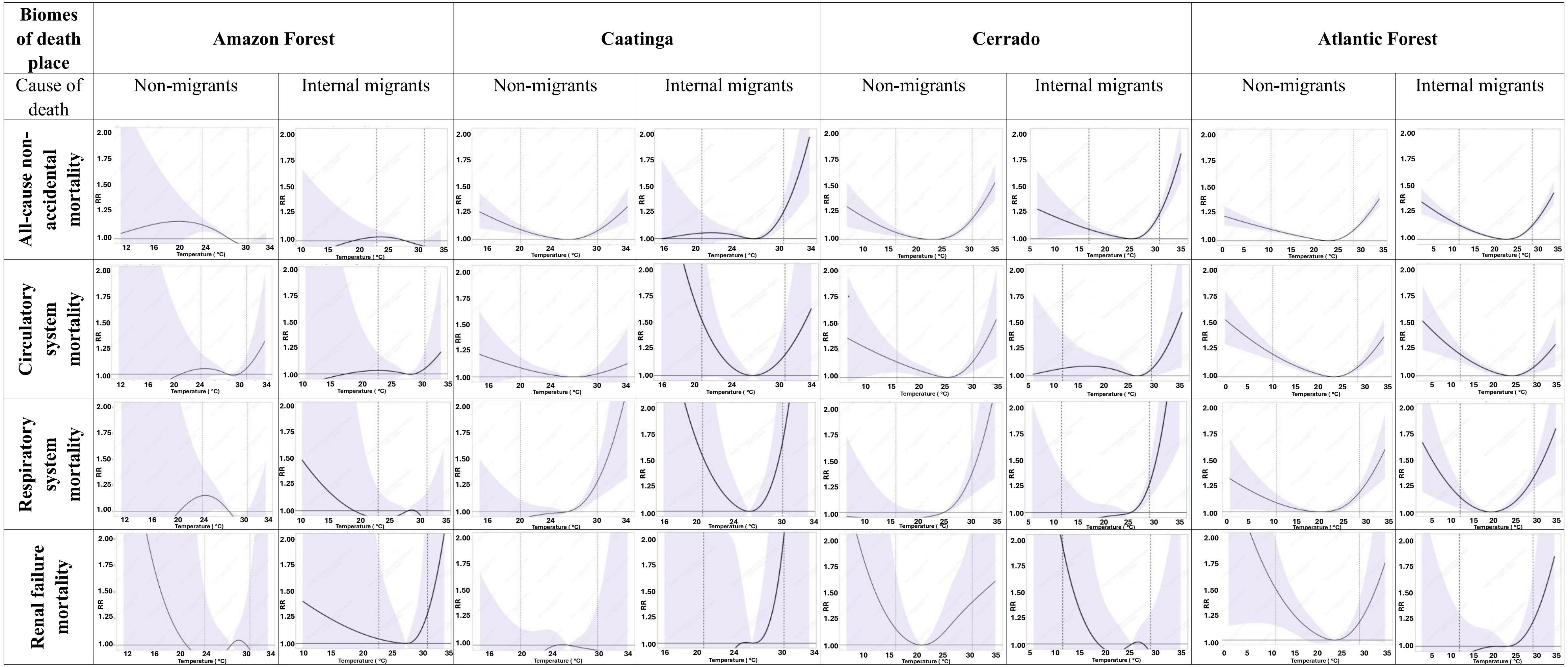

Supplementary Figure 14. Relative risks of **all-cause and cause-specific mortality** associated with extreme ambient temperature cumulated over 0-6-day lag periods by biome of the place of death. Dotted vertical lines show the 0.01 and 0.99 temperature percentiles.

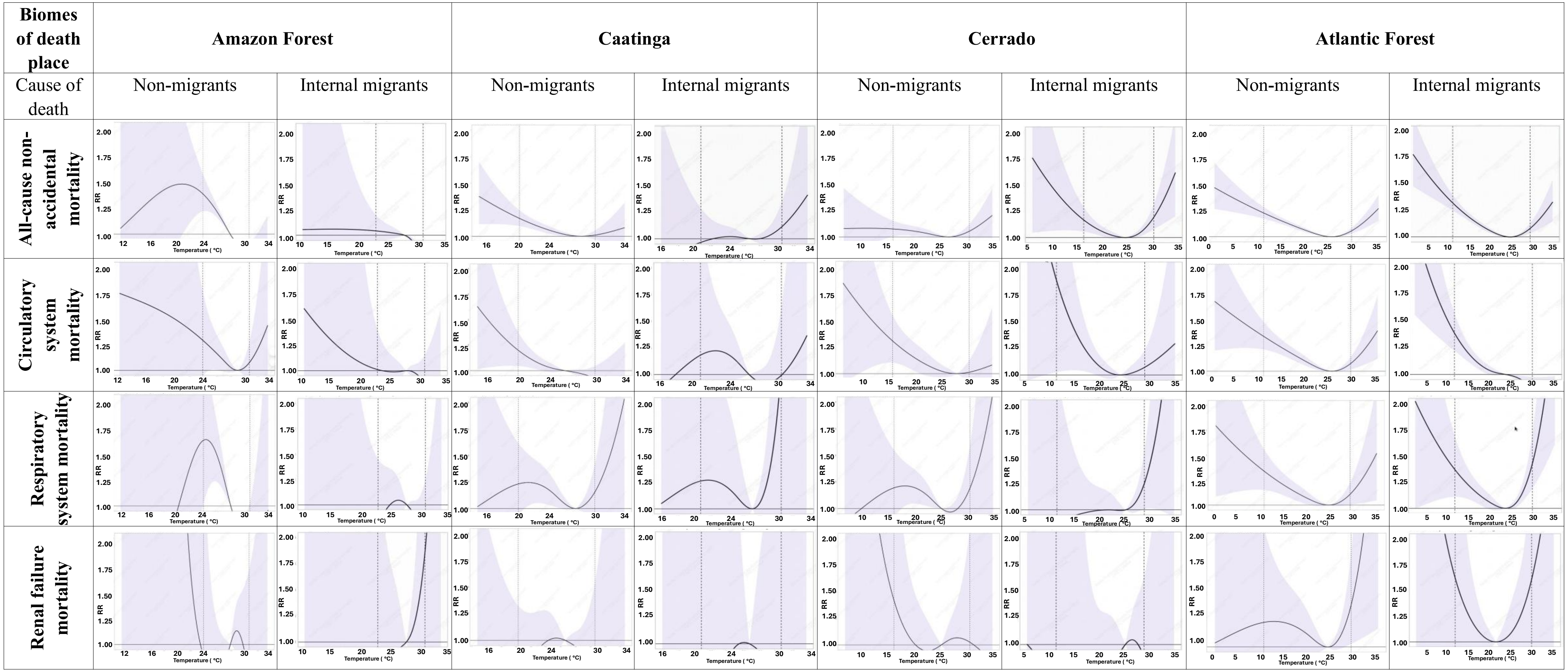

Supplementary Figure 15. Relative risks of **all-cause and cause-specific mortality** associated with extreme ambient temperature cumulated over 0-20-day lag periods by biome of the place of death. Dotted vertical lines show the 0.01 and 0.99 temperature percentiles.

Brazil comprises six major biomes: the Amazon Rainforest, Caatinga, Cerrado, Atlantic Forest, Pampas, and Pantanal. Because of the limited number of cases in the Pampas and Pantanal, the biome-stratified analyses were restricted to the first four biomes (Supplementary Table 1 & 2 and Supplementary Figure 14 & 15). The relatively small number of cases within each biome resulted in wide confidence intervals. Nevertheless, over the 0–6-day lag period, internal migrants tended to show a similar pattern of higher heat-related respiratory mortality risk than non-migrants, particularly in Cerrado (RR:1.54; 95% CI:1.24–1.93 vs R:1.38; 1.16–1.63) and the Atlantic Forest (RR:1.34; 1.22–1.47 vs R:1.19; 1.12–1.26).

For circulatory system mortality, extreme cold over 6 days had a stronger effect in all sub-groups, increasing the risk of mortality more than extreme heat. The risk of circulatory system mortality in extreme cold was higher in non-migrants than in internal migrants, particularly among older adults [IM:1.13(1.07-1.19) vs. NM:1.22(1.16-1.28)], literate individuals [IM:1.13(1.06-1.21) vs. NM:1.22(1.16-1.28)] (Supplementary Figure 16).

Stratified models over 0-6 days showed that illiterate migrants [1.52(95%CI:1.23-1.87)] and migrants living in brick-built houses [1.47(95%CI:1.30-1.65)] had higher risks of respiratory disease mortality than the corresponding groups of non-migrants (RR:1.23; 95% CI:1.07–1.40 and RR:1.30; 95% CI:1.21–1.40, respectively), after extreme heat (Supplementary Figure 16).

Extreme heat		Age between 18-59	Age 60 or older	Literate	Illiterate	Urban-based	Brick-built housing	Non-brick-built housing
All-cause non-accidental mortality	Internal-migrants	**1.01** (0.95 – 1.08)	**1.16** (1.13 – 1.19)	**1.11** (1.08 – 1.15)	**1.12** (1.07 – 1.17)	**1.15** (1.12 – 1.18)	**1.17** (1.14 – 1.20)	**0.99** (0.93 – 1.07)
	Non-migrants	**1.02** (1.00 – 1.05)	**1.14** (1.12 – 1.16)	**1.09** (1.07 – 1.11)	**1.07** (1.04 – 1.10)	**1.13** (1.11 – 1.15)	**1.14** (1.12 – 1.15)	**0.96** (0.93 – 0.99)
Circulatory system mortality	Internal-migrants	**1.02** (0.88 – 1.18)	**1.09** (1.02 – 1.17)	**1.11** (1.02 – 1.20)	**1.01** (0.89 – 1.14)	**1.12** (1.05 – 1.19)	**1.11** (1.03 – 1.19)	**0.95** (0.79 – 1.15)
	Non-migrants	**1.02** (1.00 – 1.09)	**1.13** (1.08 – 1.18)	**1.09** (1.04 – 1.14)	**1.06** (1.00 – 1.13)	**1.12** (1.07 – 1.16)	**1.13** (1.08 – 1.17)	**1.01** (0.92 – 1.10)
Respiratory system mortality	Internal-migrants	**1.28** (1.00 – 1.74)	**1.37** (1.23 – 1.54)	**1.33** (1.15 – 1.54)	**1.52** (1.23 – 1.87)	**1.35** (1.21 – 1.51)	**1.47** (1.30 – 1.65)	**0.96** (0.68 – 1.35)
	Non-migrants	**1.21** (1.06 – 1.37)	**1.29** (1.20 – 1.40)	**1.27** (1.16 – 1.39)	**1.23** (1.07 – 1.40)	**1.25** (1.17 – 1.34)	**1.30** (1.21 – 1.40)	**1.06** (0.88 – 1.29)

Extreme cold		Age between 18-59	Age 60 or older	Literate	Illiterate	Urban-based	Brick-built housing	Non-brick-built housing
All-cause non-accidental mortality	Internal-migrants	**1.10** (1.05 – 1.14)	**1.09** (1.07 – 1.12)	**1.07** (1.04 – 1.11)	**1.14** (1.10 – 1.18)	**1.10** (1.07 – 1.12)	**1.09** (1.07 – 1.11)	**1.09** (1.02 – 1.16)
	Non-migrants	**1.12** (1.08 – 1.15)	**1.08** (1.06 – 1.10)	**1.07** (1.05 – 1.10)	**1.11** (1.06 – 1.15)	**1.09** (1.07 – 1.11)	**1.09** (1.07 – 1.11)	**1.12** (1.08 – 1.17)
Circulatory system mortality	Internal-migrants	**1.24** (1.12 – 1.38)	**1.13** (1.07 – 1.19)	**1.13** (1.06 – 1.21)	**1.21** (1.09 – 1.33)	**1.15** (1.10 – 1.22)	**1.14** (1.08 – 1.20)	**1.21** (1.02 – 1.42)
	Non-migrants	**1.27** (1.18 – 1.37)	**1.22** (1.16 – 1.28)	**1.22** (1.16 – 1.28)	**1.23** (1.11 – 1.36)	**1.22** (1.17 – 1.27)	**1.22** (1.17 – 1.28)	**1.25** (1.12 – 1.38)
Respiratory system mortality	Internal-migrants	**1.10** (1.00 – 1.28)	**1.04** (1.00 – 1.11)	**1.10** (1.01 – 1.21)	**1.00** (0.85 – 1.17)	**1.06** (1.00 – 1.14)	**1.06** (0.98 – 1.14)	**1.07** (0.82 – 1.41)
	Non-migrants	**1.05** (0.94 – 1.17)	**1.06** (0.99 – 1.13)	**1.00** (0.97 – 1.04)	**1.02** (0.91 – 1.15)	**1.03** (0.98 – 1.09)	**1.02** (0.96 – 1.08)	**1.07** (0.95 – 1.21)

Supplementary Figure 16. Heat map of relative risks (RR) and 95% confidence intervals (CI) of all-cause and cause-specific mortality associated with extreme-heat and extreme-cold, cumulated over 0-6 days in stratified modelling for age (between 18 and 59, 60 or older), literacy (literate and illiterate), urbanicity and housing materials. Background shading is used to distinguish the three mortality outcomes (all-cause, circulatory, and respiratory mortality). Within each mortality outcome, bold text indicates the higher RR between internal migrants and non-migrants.

Supplementary Table 3. Summary of Origin and Destination Regions for Internal Migrants

	Regions	Origin	Destination
**Internal migration between birth and registry state**	North	36,161	130,221
	Northeast	523,738	173,982
	Southeast	325,387	429,767
	South	125,064	151,498
	Central-West	40,633	165,515
**Internal migration between registry and death state**	North	9,334	12,550
	Northeast	95,437	27,894
	Southeast	27,082	72,520
	South	10,691	11,457
	Central-West	6,109	24,232

At the regional level, the predominant routes were from the Northeast to the Southeast, within the Southeast, and within the Northeast (Supplementary Tables 3-5).

Supplementary Table 4. Heat map of the region matrix of origin-destination pairs for internal migrants. Cell shading reflects the magnitude of the number of internal migrants, with darker colours representing larger counts and lighter colours representing smaller counts.

Origin\Destination	North	Northeast	Southeast	South	Central-West	Total
**North**	25,296	5,388	4,410	1,007	9,394	45,495
**Northeast**	78,553	161,418	276,995	25,172	77,037	619,175
**Southeast**	21,067	29,646	171,194	67,887	62,675	352,469
**South**	7,985	2,402	38,783	66,837	19,748	135,755
**Central-West**	9,870	3,022	10,905	2,052	20,893	46,742
**Total**	142,771	201,876	502,287	162,955	189,747	1,199,636

Supplementary Table 5. Heat map of the region's matrix of origin–destination pairs across different migration routes. Cell shading reflects the magnitude of the number of internal migrants, with darker colours representing larger counts and lighter colours representing smaller counts.

Migration	Origin\Destination	North	Northeast	Southeast	South	Central-West	Total
**Internal migration between birth and registry state**	North	20,979	4,231	3,342	728	6,881	36,161
	Northeast	71,803	139,054	226,402	22,931	63,548	523,738
	Southeast	20,439	26,193	154,613	65,442	58,700	325,387
	South	7,762	2,075	35,882	60,693	18,652	125,064
	Central-West	9,238	2,429	9,528	1,704	17,734	40,633
**Internal migration between registry and death state**	North	4,317	1,157	1,068	279	2,513	9,334
	Northeast	6,750	22,364	50,593	2,241	13,489	95,437
	Southeast	628	3,453	16,581	2,445	3,975	27,082
	South	223	327	2,901	6,144	1,096	10,691
	Central-West	632	593	1,377	348	3,159	6,109

For population categories, the most frequent route was migration from areas with fewer than 200,000 inhabitants to similarly small areas (Supplementary Table 6).

Supplementary Table 6. Heat map of the population matrix of origin–destination pairs for internal migrants. Cell shading reflects the magnitude of the number of internal migrants, with darker colours representing larger counts and lighter colours representing smaller counts.

**Origin\Destination**	**<200,000**	**200,000 – 499,999**	**500,000 – 999,999**	**≥ 1 million**	**Total**
**Less than 200,000**	583,817	166,414	74,437	176,526	1,001,194
**200,000 – 499,999**	36,681	13,204	6,726	14,775	71,386
**500,000 – 999,999**	19,199	7,210	4,077	9,648	40,134
**1million & more**	42,413	15,683	9,386	19,440	86,922
**Total**	682,110	202,511	94,626	220,389	1,199,636

Supplementary Table 7. Heat map of the population matrix of origin–destination pairs across different migration time. Cell shading reflects the magnitude of the number of internal migrants, with darker colours representing larger counts and lighter colours representing smaller counts.

**Migration**	**Origin\Destination**	**<200,000**	**200,000 – 499,999**	**500,000 – 999,999**	**≥ 1 million**	**Total**
**Internal migration between birth and registry state**	**Less than 200,000**	530,325	143,450	61,230	141,504	876,509
	**200,000 – 499,999**	32,137	11,103	5,432	12,179	60,851
	**500,000 – 999,999**	16,861	6,311	3,509	8,477	35,158
	**1million & more**	38,361	13,985	8,463	17,656	78,465
**Internal migration between registry and death state**	**Less than 200,000**	53,492	22,964	13,207	35,022	124,685
	**200,000 – 499,999**	4,544	2,101	1,294	2,596	10,535
	**500,000 – 999,999**	2,338	899	568	1,171	4,976
	**1million & more**	4,052	1,698	923	1,784	8,457

We conducted sensitivity analysis by defining migration at the level of municipality. By this definition, there were 3,325,646 internal migrants and 1,887,932 non-migrants.

Supplementary Table 8. Relative risks and 95% confidence intervals of all-cause and cause-specific mortality associated with extreme ambient temperatures, cumulated over 0-6 days, across different migration subgroups.

Cause of death (Lag: 0-6 days)	Migration Status	MMT	Median	$RR_{Cold\ (1st\ percentile)}$ (95% CI)	$RR_{Heat\ (99th\ percentile)}$ (95% CI)
**All-cause, non-accidental mortality**	Internal migrants	23.9	25.2	1.09 (1.08-1.11)	1.17 (1.15-1.19)
	Non-migrants	25	25.4	1.10 (1.07-1.13)	1.05 (1.02-1.07)
**Circulatory system mortality**	Internal migrants	24.7	25.2	1.18 (1.15-1.22)	1.13 (1.09-1.17)
	Non-migrants	25	25.4	1.17 (1.09-1.25)	1.06 (1.00-1.12)
**Respiratory disease mortality**	Internal migrants	19.3	25.2	1.05 (1.01-1.08)	1.38 (1.30-1.47)
	Non-migrants	20.9	25.4	1.03 (0.94-1.14)	1.24 (1.12-1.38)
**Renal failure mortality**	Internal migrants	21.9	25.2	1.11 (0.95-1.30)	1.31 (1.11-1.56)
	Non-migrants	26	25.4	1.14 (0.79-1.66)	1.14 (0.87-1.50)

Supplementary Table 9. Relative risks and 95% confidence intervals of all-cause and cause-specific mortality associated with extreme ambient temperatures, cumulated over 0-20 days, across different migration subgroups.

Cause of death (Lag: 0-20 days)	Migration Status	MMT	Median	$RR_{Cold\ (1st\ percentile)}$ (95% CI)	$RR_{Heat\ (99th\ percentile)}$ (95% CI)
**All-cause, non-accidental mortality**	Internal migrants	25.7	25.2	1.22 (1.19-1.26)	1.05 (1.02-1.08)
	Non-migrants	35.7	25.4*	1.19 (1.13-1.26)	0.93 (0.89-0.97)
**Circulatory system mortality**	Internal migrants	28.4	25.2	1.34 (1.26-1.43)	1.00 (0.96-1.04)
	Non-migrants	28.6	25.4	1.33 (1.16-1.52)	1.00 (0.95-1.06)
**Respiratory disease mortality**	Internal migrants	24.5	25.2	1.19 (1.09-1.30)	1.20 (1.08-1.34)
	Non-migrants	29.2	25.4	1.26 (1.00-1.59)	1.00 (0.93-1.08)
**Renal failure mortality**	Internal migrants	22.3	25.2	1.19 (0.87-1.62)	1.36 (1.00-1.84)
	Non-migrants	35.7	25.4*	1.39 (0.70-2.76)	1.02 (0.64-1.63)

We conducted sensitivity analysis by using median of each group as the reference point (Supplementary Table 10 & 11).

Supplementary Table 10. Relative risks and 95% confidence intervals of all-cause and cause-specific mortality associated with extreme ambient temperatures, cumulated over 0-6 days, across different migration subgroups comparing to median.

**Cause of death (Lag: 0-6 days)**	**Migration Status**	**Median**	**$RR_{Cold\ (1st\ percentile)}$ (95% CI)**	**$RR_{Heat\ (99th\ percentile)}$ (95% CI)**
**All-cause, non-accidental mortality**	Internal migrants	25.4	1.10 (1.08-1.12)	1.14 (1.11-1.17)
	Non-migrants	25.2	1.10 (1.08-1.12)	1.12 (1.10-1.13)
**Circulatory system mortality**	Internal migrants	25.4	1.16 (1.10-1.22)	1.11 (1.05-1.18)
	Non-migrants	25.2	1.21 (1.16-1.26)	1.10 (1.07-1.14)
**Respiratory disease mortality**	Internal migrants	25.4	1.00 (0.91-1.07)	1.25 (1.13-1.38)
	Non-migrants	25.2	1.00 (0.95-1.07)	1.24 (1.17-1.31)
**Renal failure mortality**	Internal migrants	25.4	0.94 (0.70-1.27)	1.13 (0.85-1.51)
	Non-migrants	25.2	1.26 (1.03-1.54)	1.22 (1.03-1.43)

Supplementary Table 11. Relative risks and 95% confidence intervals of all-cause and cause-specific mortality associated with extreme ambient temperatures, cumulated over 0-20 days, across different migration subgroups comparing to median.

**Cause of death (Lag: 0-20 days)**	**Migration Status**	**Median**	**$RR_{Cold\ (1st\ percentile)}$ (95% CI)**	**$RR_{Heat\ (99th\ percentile)}$ (95% CI)**
**All-cause, non-accidental mortality**	Internal migrants	25.4	1.27 (1.22-1.32)	1.05 (1.01-1.10)
	Non-migrants	25.2	1.24 (1.20-1.28)	1.01 (0.98-1.03)
**Circulatory system mortality**	Internal migrants	25.4	1.32 (1.20-1.45)	0.93 (0.84-1.03)
	Non-migrants	25.2	1.39 (1.29-1.49)	1.03 (0.97-1.09)
**Respiratory disease mortality**	Internal migrants	25.4	1.26 (1.08-1.47)	1.28 (1.06-1.54)
	Non-migrants	25.2	1.28 (1.15-1.43)	1.05 (0.95-1.17)
**Renal failure mortality**	Internal migrants	25.4	1.15 (0.64-2.05)	1.27 (0.75-2.14)
	Non-migrants	25.2	1.12 (0.77-1.62)	1.19 (0.90-1.58)

We conducted sensitivity analyses for genitourinary mortality using both the minimum mortality temperature and the median temperature as reference points. Significant associations were observed only after extreme heat. Risks were generally comparable between internal migrants and non-migrants at longer lags, although non-migrants exhibited slightly higher risks than internal migrants at the shorter lag period (0-6 days) (Supplementary Table 12).

Supplementary Table 12. Relative risks and 95% confidence intervals of genitourinary system mortality associated with extreme ambient temperatures, cumulated over lag periods, comparing to both minimum mortality temperature and median.

**Lag periods**	**Migration Status**	**Median**	**$RR_{Cold\ (1st\ percentile)}$** (95% CI)	**$RR_{Heat\ (99th\ percentile)}$** (95% CI)
**Lag: 0-6 days**	Internal migrants	25.4	0.94 (0.78-1.13)	1.26 (1.05-1.52)
	Non-migrants	25.2	1.07 (0.93-1.23)	1.30 (1.17-1.45)
**Lag: 0-20 days**	Internal migrants	25.4	1.17 (0.82-1.68)	1.22 (0.86-1.72)
	Non-migrants	25.2	1.10 (0.85-1.42)	1.25 (1.02-1.52)
**Lag periods**	**Migration Status**	**MMT**	**$RR_{Cold\ (1st\ percentile)}$** (95% CI)	**$RR_{Heat\ (99th\ percentile)}$** (95% CI)
**Lag: 0-6 days**	Internal migrants	14.4	1.00 (0.96-1.04)	1.34 (1.06-1.68)
	Non-migrants	22.1	1.09 (0.96-1.25)	1.33 (1.19-1.48)
**Lag: 0-20 days**	Internal migrants	21.5	1.24 (0.86-1.78)	1.28 (0.91-1.81)
	Non-migrants	21.8	1.13 (0.87-1.46)	1.28 (1.05-1.55)

Sensitivity analyses were conducted using alternative specifications of knot placement (p10 and p90) in the temperature cross-basis.

Supplementary Table 13. Relative risks and 95% confidence intervals of all-cause and cause-specific mortality associated with extreme ambient temperatures, cumulated over 0-6 days, using a different knot placement (p10 and p90) in the temperature cross-basis.

Cause of death (Lag: 0-6 days)	Migration Status	MMT	Median	$RR_{Cold\ (1st\ percentile)}$ (95% CI)	$RR_{Heat\ (99th\ percentile)}$ (95% CI)
All-cause, non-accidental mortality	Internal migrants	23.7	25.4	1.11 (1.09-1.13)	1.14 (1.11-1.17)
	Non-migrants	23.7	25.2	1.10 (1.08-1.12)	1.12 (1.10-1.14)
Circulatory system mortality	Internal migrants	24.5	25.4	1.16 (1.11-1.22)	1.11 (1.05-1.17)
	Non-migrants	24.6	25.2	1.21 (1.16-1.26)	1.10 (1.06-1.13)
Respiratory disease mortality	Internal migrants	19.4	25.4	1.08 (1.01-1.16)	1.38 (1.25-1.52)
	Non-migrants	20.4	25.2	1.05 (0.99-1.10)	1.29 (1.21-1.37)
Renal failure mortality	Internal migrants	2.7	25.4*	0.94 (0.70-1.27)	1.13 (0.86-1.49)
	Non-migrants	23.1	25.2	1.28 (1.05-1.55)	1.23 (1.04-1.45)

Supplementary Table 14. Relative risks and 95% confidence intervals of all-cause and cause-specific mortality associated with extreme ambient temperatures, cumulated over 0-20 days, using a different knot placement (p10 and p90) in the temperature cross-basis.

Cause of death (Lag: 0-20 days)	Migration Status	MMT	Median	$RR_{Cold\ (1st\ percentile)}$ (95% CI)	$RR_{Heat\ (99th\ percentile)}$ (95% CI)
All-cause, non-accidental mortality	Internal migrants	25.5	25.4	1.27 (1.21-1.32)	1.06 (1.01-1.10)
	Non-migrants	27.2	25.2	1.24 (1.20-1.28)	1.01 (0.99-1.03)
Circulatory system mortality	Internal migrants	35.7	25.4*	1.33 (1.21-1.46)	0.93 (0.84-1.03)
	Non-migrants	27.1	25.2	1.40 (1.30-1.51)	1.03 (0.99-1.08)
Respiratory disease mortality	Internal migrants	22.8	25.4	1.28 (1.10-1.50)	1.34 (1.12-1.61)
	Non-migrants	26.0	25.2	1.28 (1.15-1.43)	1.05 (0.95-1.15)
Renal failure mortality	Internal migrants	20.2	25.4	1.35 (0.75-2.40)	1.41 (0.83-2.37)
	Non-migrants	23.3	25.2	1.12 (0.77-1.62)	1.21 (0.91-1.62)

We conducted a sensitivity analysis to assess effect modification by migration status by including interaction terms between the temperature cross-basis and migration status. The results showed patterns consistent with the main analysis, with higher extreme heat-related respiratory mortality among internal migrants compared with non-migrants, over both short and long lags.

Supplementary Table 15. Relative risks and 95% confidence intervals of all-cause and cause-specific mortality associated with extreme ambient temperatures, cumulated over 0-6 days, by migration status, using interaction terms between temperature cross-basis and migration status

Cause of death (Lag: 0-6 days)	Migration Status	MMT	Median	$RR_{Cold\ (1st\ percentile)}$ (95% CI)	$RR_{Heat\ (99th\ percentile)}$ (95% CI)	Likelihood ratio test
All-cause, non-accidental mortality	Non-migrant	23.3	25.2	1.06 (1.05-1.08)	1.12 (1.10-1.13)	P = 0.08
	Internal migrants	22.6	25.2	1.05 (1.03 – 1.07)	1.14 (1.12-1.16)	
Circulatory system mortality	Non-migrant	24.6	25.2	1.14 (1.10 – 1.17)	1.09 (1.06 – 1.12)	P = 0.65
	Internal migrants	24.1	25.2	1.09 (1.04 – 1.14)	1.09 (1.04 – 1.14)	
Respiratory disease mortality	Non-migrant	17.6	25.2	1.02 (0.98 – 1.05)	1.30 (1.23 – 1.37)	P = 0.69
	Internal migrants	17.1	25.2	1.03 (0.98 – 1.08)	1.37 (1.26 – 1.48)	
Renal failure mortality	Non-migrant	21.5	25.2	1.14 (0.98 – 1.33)	1.20 (1.04 – 1.39)	P = 0.65
	Internal migrants	35.7*	25.2	0.98 (0.75 – 1.28)	0.98 (0.78 – 1.23)	

Supplementary Table 16. Relative risks and 95% confidence intervals of all-cause and cause-specific mortality associated with extreme ambient temperatures, cumulated over 0-20 days, by migration status, using interaction terms between temperature cross-basis and migration status.

**Cause of death (Lag: 0-20 days)**	**Migration Status**	**MMT**	**Median**	**$RR_{Cold\ (1st\ percentile)}$ (95% CI)**	**$RR_{Heat\ (99th\ percentile)}$ (95% CI)**	**Likelihood ratio test**
**All-cause, non-accidental mortality**	Non-migrant	24.9	25.2	1.18 (1.15 – 1.20)	1.08 (1.06 – 1.10)	P = 0.15
	Internal migrants	24.5	25.2	1.17 (1.13 – 1.21)	1.10 (1.06 – 1.13)	
**Circulatory system mortality**	Non-migrant	25.5	25.2	1.34 (1.27 – 1.42)	1.10 (1.06 – 1.15)	P = 0.89
	Internal migrants	25.5	25.2	1.31 (1.21 – 1.41)	1.09 (1.01 – 1.16)	
**Respiratory disease mortality**	Non-migrant	23.1	25.2	1.17 (1.08 – 1.27)	1.20 (1.11 – 1.30)	P = 0.33
	Internal migrants	20.8	25.2	1.12 (1.00 – 1.26)	1.42 (1.26 – 1.60)	
**Renal failure mortality**	Non-migrant	23.3	25.2	1.12 (0.85 – 1.48)	1.23 (0.98 – 1.54)	P = 0.64
	Internal migrants	0.8*	25.2	0.95 (0.61 – 1.48)	1.16 (0.80 – 1.67)	
